# Eye Tracking—An Innovative Tool in Medical Parasitology

**DOI:** 10.3390/jcm10132989

**Published:** 2021-07-04

**Authors:** Przemysław Kołodziej, Wioletta Tuszyńska-Bogucka, Mariusz Dzieńkowski, Jacek Bogucki, Janusz Kocki, Marek Milosz, Marcin Kocki, Patrycja Reszka, Wojciech Kocki, Anna Bogucka-Kocka

**Affiliations:** 1 Chair and Department of Biology and Genetics, Medical University of Lublin, 20-093 Lublin, Poland; anna.kocka@umlub.pl; 2 Department of Human Sciences, University of Economics and Innovation in Lublin, 20-209 Lublin, Poland; wioletta.tuszynska.bogucka@wp.pl; 3 Department of Computer Science, Lublin University of Technology, 20-618 Lublin, Poland; m.dzienkowski@pollub.pl (M.D.); m.milosz@pollub.pl (M.M.); 4 Department of Organic Chemistry, Medical University of Lublin, 20-093 Lublin, Poland; jacek.bogucki@umlub.pl; 5 Department of Clinical Genetics, Medical University of Lublin, 20-080 Lublin, Poland; janusz.kocki@umlub.pl; 6 Scientific Circle at Department of Clinical Genetics, Medical University of Lublin, 20-080 Lublin, Poland; 53574@student.umlub.pl (M.K.); 55644@student.umlub.pl (P.R.); 7 Department of Architecture and Urban Planning, Lublin University of Technology, 20-618 Lublin, Poland; w.kocki@pollub.pl

**Keywords:** medical parasitology, parasitological diagnostics, e-parasitology, eye tracking, eye movement modelling examples, medical education, virtual microscopy

## Abstract

The innovative Eye Movement Modelling Examples (EMMEs) method can be used in medicine as an educational training tool for the assessment and verification of students and professionals. Our work was intended to analyse the possibility of using eye tracking tools to verify the skills and training of people engaged in laboratory medicine on the example of parasitological diagnostics. Professionally active laboratory diagnosticians working in a multi-profile laboratory (non-parasitological) (*n* = 16), laboratory diagnosticians no longer working in this profession (*n* = 10), and medical analyst students (*n* = 56), participated in the study. The studied group analysed microscopic images of parasitological preparations made with the cellSens Dimension Software (Olympus) system. Eye activity parameters were obtained using a stationary, video-based eye tracker Tobii TX300 which has a 3-ms temporal resolution. Eye movement activity parameters were analysed along with time parameters. The results of our studies have shown that the eye tracking method is a valuable tool for the analysis of parasitological preparations. Detailed quantitative and qualitative analysis confirmed that the EMMEs method may facilitate learning of the correct microscopic image scanning path. The analysis of the results of our studies allows us to conclude that the EMMEs method may be a valuable tool in the preparation of teaching materials in virtual microscopy. These teaching materials generated with the use of eye tracking, prepared by experienced professionals in the field of laboratory medicine, can be used during various training, simulations and courses in medical parasitology and contribute to the verification of education results, professional skills, and elimination of errors in parasitological diagnostics.

## 1. Introduction

A significant increase in interest in eye tracking methods in medicine has been observed recently. The study of eye movement activity conducted with the use of state-of-the-art eye tracking technology can be a valuable tool in preparing teaching materials for parasitology education and a tool that can be used to evaluate and verify the work of students, graduates, and specialists in all health professions [1,2,3]. The eye tracking method can also serve as a valuable instrument in helping to determine the probable sources of errors in the interpretation of a medical image. So far, eye tracking has been used mostly in radiology, breast pathology, general surgery, neurology, emergency medicine, anaesthesiology, ophthalmology, histopathology, and cardiology [4,5,6,7,8,9]. Among others, eye tracking studies are used in clinical education, comparison of professional skills of young physicians and specialists (e.g., in radiology), the analysis of databases as a tool to identify priority information important in clinical decision-making, and the assessment of surgical skills [10,11,12,13,14,15].

Development and improvement of new forms of parasitology education and ongoing COVID-19 pandemic require the use of modern teaching methods and techniques, among which eye tracking, MultiMatch, SubsMatch 2.0 (Eberhart Karls University Tubingen, Tubingen, Germany) deserves special attention. The acquisition of knowledge and improvement of skills by students and young physicians based on Eye Movement Modelling Examples has proved to be both promising and effective [1,4,16,17,18,19,20,21].

Microscopic methods are an important diagnostic tool in various fields of laboratory medicine and still constitute the so-called gold standard in, among others, haematological, parasitological, microbiological, mycological, and cytomorphological diagnostics and genetics; therefore, it is also worth applying EMMEs in microscopic diagnostics. To the best of our knowledge, the literature to date does not provide any information on the use of the described method in medical parasitology. Our research focused on the use of eye tracking technology in parasitological diagnostics.

Our work aims to explore the possibility of using eye tracking tools in virtual education in the parasitology and verification of skills of students and people engaged in laboratory medicine. We used the “find-recognize” model. The specific objectives of our work include: (1) assessment of the ability to detect the parasitic form (find), (2) assessment of the ability to recognise and identify the parasitic form (recognize). For the first objective, we look for errors and irregularities in the preparation analysis (e.g., too quick completion of the search or failure to search the entire preparation field). The second objective is to assess the correct species identification of the parasite (i.e., selecting the correct answer from among the five options given).

## 2. Materials and Methods

### 2.1. Participants

Originally, there were 83 participants in the study. However, data on one of the participants was discarded due to a too low value of the tracking ratio (<30%), which was caused by the person’s astigmatism. The remaining 82 participants in the experiment, who were adults with normal or corrected-to-normal vision, were classified into three groups. The first group (Expert A) included 16 professionally active experts working in a multi-profile laboratory (non-parasitological). The second group (Expert B) consisted of 10 experts with academic background in biology and laboratory diagnostics, but not currently working in laboratories. The last group encompassed 56 medical analytics students (Students), who were either doing, or just finishing, a parasitological analysis course. The age average was: Experts A, 35.62, Experts B, 38.50, and Students, 20.12. Women constituted a great majority of the subjects: Experts A, 93.75%, Experts B, 100%, and Students, 80.36%.

### 2.2. Apparatus

**Figure 1:**
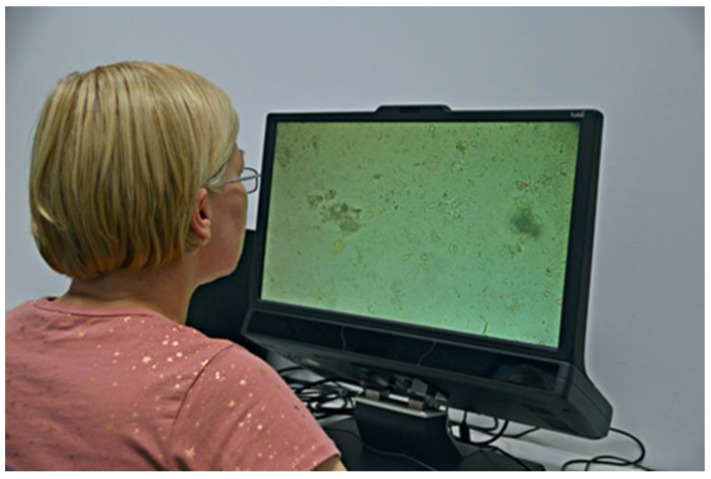
Eye tracking study with an eye tracker (Tobii TX300)—person controlling a digital image of a parasitological preparation, participating in the study.

Eye activity parameters were obtained using a stationary, binocular video-based eye tracker Tobii TX300 (Tobii AB, Stockholm, Sweden), which has a 3-ms temporal resolution. Slides of microscopic preparations were displayed on a 23-inch TFT LCD (a variant of a Transistor Liquid Crystal Display (LCD) that uses Thin Film Transistor Liquid (TFT) technology, Tobii AB, Stockholm, Sweden), a 1920 × 1080 pixels widescreen monitor integrated with the eye tracker unit. The experiment was run on an Asus G750JX-T4191H laptop (Intel Core i7-4700HQ, 8GB RAM, Windows 10, ASUS, Taipei, Taiwan) and conducted by means of Tobii Studio 3.3.2 software (Tobii AB, Stockholm, Sweden) and a specially developed application, which was used for gathering subjects’ metric data for presentation of the visual stimuli and for saving the participants’ answers concerning what they recognised on subsequent preparations. The measurement of the distance between the device and the tested person is performed automatically by the hardware and software of the device. The distance between the screen and the participants ranged from 59 to 65 cm. This is the distance from the eye of a tested person (Figure 1) [22].

The eye tracker records, among others, two types of information: fixations (mainly related to information processing)—points where the respondent’s eyesight has been stopped (focused); and saccades (information retrieval)—eye movements between fixation points. Focusing one’s fixation on a certain area means that it is more visible than other areas. One of the most commonly used measures of eye tracking is the duration of fixation (also called fixation time). The number of fixations on a given element tells us, among other things, about the efficiency of searching the examined object, its importance and visibility in the process of visual scanning [23,24,25]. Saccade movements can be defined as quickly reaching speeds of up to 500–700°/s—a vector quantity describing the rotational movement of the eyeball is the angular velocity vector value, short-lasting and precise movements performed in order to locate the image on the central fovea of the retina [23,26,27,28,29]. Saccades are triggered in response to the need to immediately change the direction of looking. This makes the observed object more visible. The number of saccades is closely related to the spatial organisation of information in the stimuli. The brain directs the eyes to focus on subsequent areas of interest, e.g., individual elements of a complex visual scene [30,31]. Thus, eye movements are necessary for the perception of scenes that spread over an area greater than a few degrees in the subject’s field of view.

The analysis of the manner in which the experts and the students scanned the microscopic images showing various forms of parasites was based on the qualitative and quantitative scan-paths (patterns of fixations and saccades).

In recent years, many novel and advanced algorithms for comparing and classifying scan-paths have been developed of which at least a few approaches deserve special attention: a geometric vector-based approach (MultiMatch, Eberhart Karls University Tubingen, Tubingen, Germany) [18], random ferns in combination with saccade angle successions [19], and classification based on subsequence frequencies (SubsMatch 2.0, Eberhart Karls University Tubingen, Tubingen, Germany) [20]. Another innovative approach to scan path classification is via a combination of unsupervised feature learning and convolutional neural networks in which an Emoji space representation as feature space is used as an amusement factor [21]. The authors of this work intended to conduct analyses in a manner comprehensible to doctors, clinicians and psychologists, i.e., professionals who do not use advanced data analysis such as machine learning algorithms and neural networks.

### 2.3. Material

**Figure 2:**
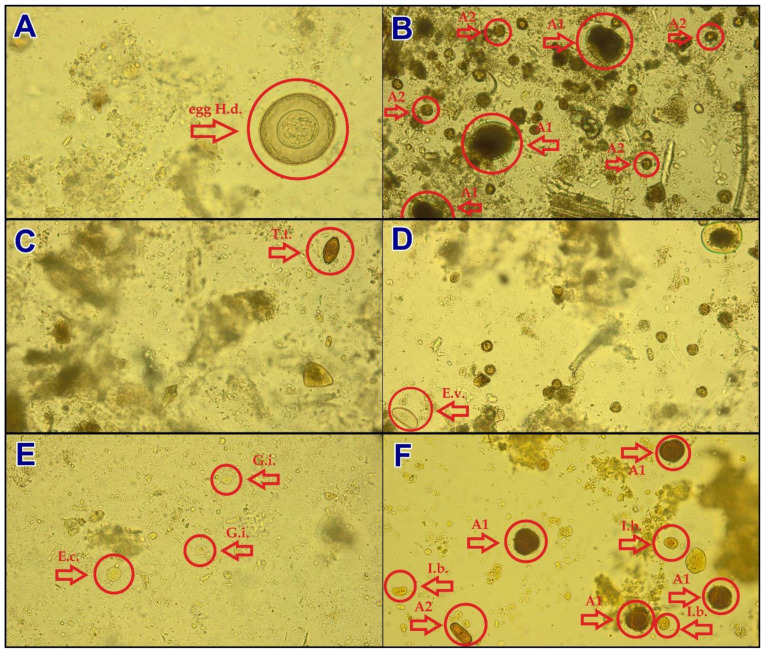
Photographs of microscopic preparations used in the study (diagnostic elements have been marked with a red arrow and a circle). (**A**). *Hymenolepis diminuta* (H.d.), embryonated egg in faeces (400× magnification); (**B**). Artefacts (A1, A2), pseudo-parasites (200× magnification); (**C**). *Trichuris trichiura* (T.t.), unembryonated egg in faeces (200× magnification); (**D**). *Enterobius vermicularis* (E.v.), egg in faeces (200× magnification); (**E**). *Giardia intestinalis* (G.i.) and *Entamoeba* sp. (E.c.), cysts in faeces (400× magnification); (**F**). *Iodamoeba bütschlii* (I.b.), cysts and artefacts (A1, A2) in faeces (400× magnification).

The material used for testing included faeces samples subjected to appropriate preparation. The preparations were concentrated with the sedimentation method, collected on a slide, coloured with Lugol liquid, and covered with a lid [32]. They were inspected under an optical microscope at magnifications of 200× and 400× (Olympus). Then, using the cellSens Dimension Software (Olympus, Tokyo, Japan) designed to analyse the microscopic images, we prepared photographs showing various diagnostic forms of human intestinal parasites. For our study, we selected six photographs of preparations prepared for diagnostics, which included: A. *Hymenolepis diminuta* (H.d.)—embryonated egg in faeces (400× magnification); B. Artefacts (A1, A2)—pseudo-parasites, undigested remnants in faeces (200× magnification); C. *Trichuris trichiura* (T.t.)—unembryonated egg in faeces (200× magnification); D. *Enterobius vermicularis* (E.v.)—egg in faeces (200× magnification); E. *Giardia intestinalis* (G.i.) and *Entamoeba* sp. (E.c.)—cysts in faeces (400× magnification); F. *Iodamoeba bütschlii* (I.b.)—cysts and artefacts (A1, A2) in faeces (400× magnification) (Figure 2).

### 2.4. Procedure

At the beginning of the experiment, each participant signed a consent form and was asked to sit in an upright chair, in a quiet testing room with artificial lighting. The participants were instructed to minimise body movements and to keep their gaze directed toward the screen during experimental tasks. A calibration procedure was conducted using a nine-point calibration process.

Before starting the study, each participant had the operation of the apparatus thoroughly explained to them and was acquainted with (1) the purpose of the research, (2) the research procedure, and (3) the number of presented preparations [22,33].

The research procedure consisted of:

Trial analysis: Analysis of one trial preparation (containing plant pollen—an artefact, without a parasitic form), which familiarised the participants with the course of the study. The result of the preparation analysis was not included in the result of the proper examination.

Proper analysis:

(1) The study participant analysed analyse six parasitological preparations (*Hymenolepis diminuta*, artefacts, *Trichuris trichiura*, *Enterobius vermicularis*, *Giardia intestinalis* and *Entamoeba* sp., *Iodamoeba bütschlii* and artefacts.
(2) The slides were displayed in a predetermined order, each for 60 s; however, the participants could make the decision to stop watching the slides before the specified time deadline.
(3) After watching each slide, the participant had to decide what he/she saw on the presented preparation, and in the questionnaire he/she had to tick one or more checkbox.
(4) The multiple-choice questions encompassed five options: three parasites, artefacts and an “I don’t know” option.
(5) It is worth adding that all the participants of the study had valid medical qualifications, enabling them to study medical faculties and practice the profession, which eliminates the possibility of distorting the test results by possible vision defects. The presented preparations did not contain any content that was not discussed during the didactic classes in which the surveyed students participated.

### 2.5. Statistics

The χ^2^ Pearson test was used to compare the distribution of the answers (true and false diagnosis) given by the study participants. The Mann-Whitney U test was used to compare the values of selected eye tracker parameters. All analyses were performed using the Statistica v.13.3 software (Tibco Corporation, Palo Alto, CA, USA).

## 3. Results

The results obtained during the experiments were subjected to a thorough quantitative and qualitative analysis in order to check the eye tracking method as an effective tool for analysing microscopic images of parasitological preparations.

### 3.1. Quantitative Analysis

Quantitative analysis carried out in three groups included a classical analysis of the significance of differences in the basic parameters of eye movement activity (number of fixations, mean fixation duration, number of saccades, mean saccade duration, mean amplitude of saccade, number of horizontal saccades, number of vertical saccades and number of diagonal saccades) and time parameters (duration of visual stimulus display, duration of questionnaire display involving participant decision-making time). The second stage of the quantitative analysis included a comparison of eye movement activity parameters for groups of people (regardless of the initial division into groups) with the largest and smallest number of correct diagnoses. The study results were as follows:

(1) *Hymenolepis diminuta*. The analysis showed statistically significant differences in the distribution of diagnoses across respondents from the examined groups. Experts B (100%) and Students (85.71%) provided the highest number of incorrect diagnoses, compared to 43.75% by Experts A. The difference was statistically significant.
(2) Artefacts. The analysis did not show statistically significant differences in the distribution of diagnoses across respondents from the examined groups. Experts B (70%) and Experts A (56.25%) made the most incorrect diagnoses, with Students (64.29%) providing the highest percentage of correct diagnoses. However, the difference was not statistically significant.
(3) *Trichuris trichiura*. The analysis showed statistically significant differences in the distribution of diagnoses across respondents from the examined groups. Experts B (80%) provided for the highest number of incorrect diagnoses, whereas Experts A (100%) and Students (94.64%) made most of the correct ones. The difference was statistically significant.
(4) *Enterobius vermicularis*. The analysis showed statistically significant differences in the distribution of diagnoses across respondents from the examined groups. Experts B (90%) provided the highest number of incorrect diagnoses, whereas Students (76.79%) and Experts A (62.50%) made most of the correct ones. The difference was statistically significant.
(5) *Giardia intestinalis* and *Entamoeba* sp. The analysis showed statistically significant differences in the distribution of diagnoses across respondents from the examined groups. Experts B (100%) and Students (96%) provided the highest number of incorrect diagnoses, compared to 25% by Experts A. The difference was statistically significant.
(6) *Iodamoeba bütschlii* and artefacts. The analysis did not show any significant differences in the distribution of correct diagnoses in respondents from the examined groups—incorrect diagnoses in all groups exceeded 80% (Supplementary Figure S1, Table 1).

We also analysed the parameters of eye movement activity of those people (regardless of the initial group assignment) who obtained the worse (Group I, *n* = 18, less than 16.7% correct answers) and the best (Group II, *n* = 11, more than 66.7% correct answers) results in the number of correct diagnoses of the presented preparations. The cut-off point was calculated on the basis of the percentile analysis (the group with the lowest scores were those below the 10th percentile, and those with the highest scores were above the 90th percentile). Eye movement activity parameters were analysed: number of fixations, mean fixation duration, number of saccades, mean saccade duration, mean amplitude of saccade, number of horizontal saccades, number of vertical saccades, number of diagonal saccades. We also analysed time parameters: duration of visual stimulus display, duration of questionnaire display involving the participant decision-making time. The analysis showed a statistically significant difference in the number of horizontal saccades during the analysis of preparations (U = 40.500, Z = −1.998, *p* = 0.045). The subjects with the highest number of correct parasitological diagnoses (Group II) performed significantly more horizontal saccades during the analysis compared to those with the lowest number of correct diagnoses. Detailed data can be found in the “Supplementary Materials” section, Supplementary Table S1, Figures S2–S12.

The eye tracking method may in the future turn out to be a tool used to verify the work of laboratory diagnosticians and to prevent errors. The analysis of a preparation on which we placed two species of parasites can serve as such an example. The analysis showed that in expert group A (professionally active experts), only 25% of the respondents made a correct diagnosis. This proves that even experienced experts make mistakes and find only one characteristic diagnostic form. The results obtained can serve as a useful example in the education of medical analyst students and professionally active experts.

The eye tracking tool can contribute to the creation of web-based, life-long learning courses and verification of skills. Eye tracking use in the analysis of the visual space of preparations indicated the causes of diagnostic errors.

### 3.2. Qualitative Analysis

The qualitative analysis included scanning paths and heatmaps. Heatmaps represent the image as seen by the subject with areas of different sizes and colours of different intensity, which are directly proportional to the time and frequency of focus on specific areas. The warmer the colours (red), the more time spent and the greater the frequency of glances. The “Hot Areas” are therefore the areas that have attracted the most attention of the subjects. Scanning paths are a visualised representation of the way a diagnostic preparation is inspected by the eye, which makes it possible, for example, to determine the trend in the direction of saccades or the number of fixations.

**Figure 3:**
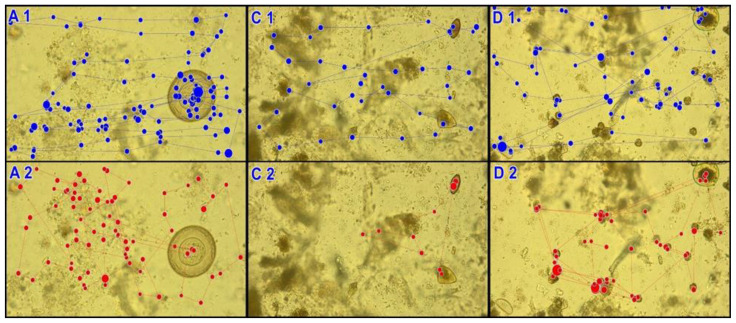
Example scanning paths based on the analysis of preparation A, *Hymenolepis diminuta*, C, *Trichuris trichiura*, and D, *Enterobius vermicularis* (1, correct diagnosis, 2, incorrect diagnosis. Dot-fixation (eye hold); dot diameter is directly proportional to the time the eye is held. Line-saccade (quick shift of eyesight from one point to another).

To supplement the statistical data, we performed a qualitative analysis of the results collected with the use of an eye tracker, which represented the participants’ method of visual preparation analysis. The qualitative analysis (Figure 3, Supplementary Video S1–S4) shows examples of activities, mainly based on horizontal meandering (parts 1) typical for people who made accurate diagnoses. In individual images, it can be seen that the scanning path covers the entire surface of the preparation. Part 2 exhibits chaotic scanning paths characterised by short and uneven saccades. As a result, the entire preparation area is not analysed and incorrect diagnosis is made. Detailed data can be found in the “Supplementary Materials” section, Supplementary Figures S13–S18.

**Figure 4:**
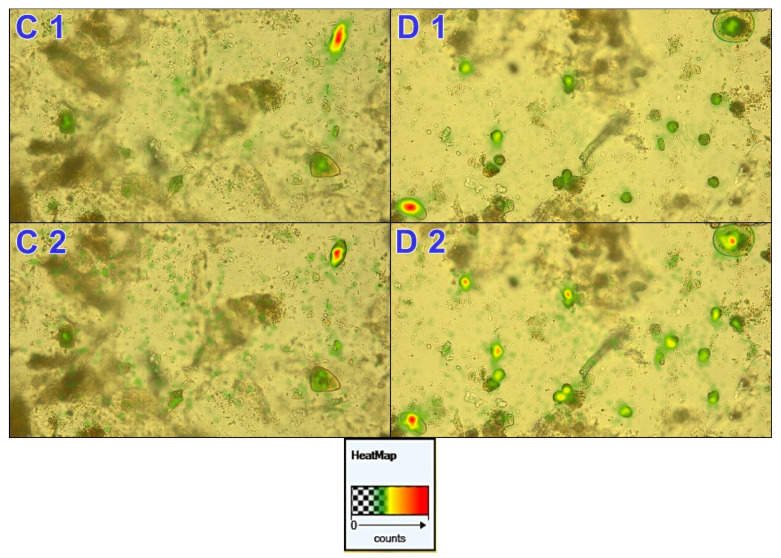
Example heatmap based on the analysis of preparation C, *Trichuris trichiura*, and D, *Enterobius vermicularis* (1, correct diagnosis of all participants, 2, incorrect diagnosis of all participants). A heatmap is a type of information showing the number of gazes (fixations), i.e., how many times the respondents looked at a given area.

Thermal maps indicate that most participants focused mainly on the diagnostic forms of parasites present in the preparation. The subjects from the group that correctly recognised the preparation looked at the parasitic form more often. The more glances the respondents needed to get acquainted with a given area, the redder the area (Figure 4). Detailed data can be found in the “Supplementary Materials” section, Supplementary Figures S19–S22.

**Figure 5:**
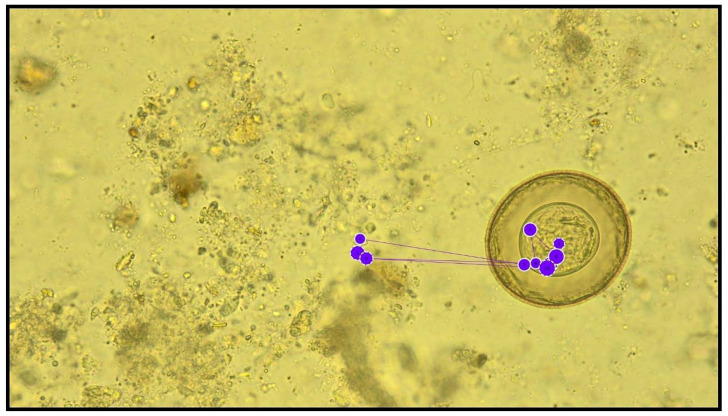
Example of incorrect, too narrow analysis of a preparation. Dot-fixation (eye hold); dot diameter is directly proportional to the time the eye is held. Line-saccade (quick shift of eyesight from one point to another).

A considerable observation, as shown in Figure 5, was made during the analysis. The subject, while analysing the preparation, only made a few short saccades (in a minority of horizontal cases), the object was found and marked, and the subject gave up further search of the preparation area. This is indicative of routine at work, which may result in an incorrect diagnosis. Finding one diagnostic form does not exclude the presence of other forms in the examined material.

The use of eye tracking as a tool for the analysis and evaluation of laboratory diagnostician’s work enables the verification of his or her professional competence and becomes a useful tool for training.

The results of the qualitative analysis show that the way the preparation is searched could possibly be related to a correct diagnosis. The subjects with the highest scores more often than the subjects with the lowest scores reported search patterns based on horizontal saccade activity. It seems that a meandering horizontal search (based on horizontal saccades) is an effective search pattern (Supplementary Table S1, Figure S10—quantitative analysis and Figures S13–S18—qualitative analysis). However, the trend detected in our study requires verification in studies on larger groups.

Teaching materials prepared by experts and experienced specialists, generated with the use of the eye tracking device, illustrating the correct way of scanning and analysing microscopic specimens, may become an interesting tool to teach practical skills in medical parasitology. This will allow students, medics and people who, after graduation, have not practiced their profession to acquire proper microscopic image analysing skills. Additionally, with the use of suitable IT tools, it is possible to develop appropriate software or applications that would further increase the possibilities of using the EMMEs method in parasitological diagnosis.

## 4. Discussion

The development of new technologies in a computerised society has created a need for major changes in medical education worldwide, including in parasitology curricula. For years, the demand for the use of modern computer technologies, telemedicine and tele-microbiology in medical education and clinical practice has been increasing. The COVID-19 pandemic has substantially increased demand for broadly understood telemedicine [16,17,34,35,36,37,38]. The main task faced by medical universities is the education of practical skills in all medical courses. This task is difficult; therefore, it requires the use of modern computer, IT and telecommunication technologies. There is an urgent need to search for new, effective teaching methods in medical education, including parasitology [16,34,35,36,37,38]. All over the world, specialists in the field of parasitology exchange their experience and insights on the virtual teaching of parasitology [16,37,39,40,41,42,43,44]. The use of appropriate methods in the field of telemedicine, telemicrobiology, and tele-microscopy can be extremely useful in the course of teaching parasitology, but can also be important in diagnostics and clinical practice. Worldwide telediagnosis of parasitic diseases is offered by The Centers for Disease Control and Prevention’s Division of Parasitic Diseases and Malaria Diagnostic Assistance Service (DPDx) [37,44,45].

The EMMEs method can be a useful model that constitutes an innovative element in teaching microscopic image analysis, e.g., in parasitological diagnostics. Implementation of eye tracking in medical education may facilitate the learning of correct pathways in searching microscopic images (correct eye movement), which in the future will facilitate the work of laboratory diagnosticians and reduce the risk of incorrect and incomplete diagnoses. After analysing the data collected with the eye tracking method, we can see that search errors could possibly be related to a correct or incorrect diagnosis. However, the trend detected in our study requires verification in studies on larger groups. According to Brunyé, recognition errors occur when the eyes fix on an object, but the object was not properly recognised or was not recognised as relevant or valuable for the search task. A search error occurs when the eyes fail to determine the critical area in a visual scene, leading to failure to detect the object. These may also be called scan errors because the critical function was not on the scanning path [1]. Our results show that the participants had problems focusing on the critical visual information in the presented image. This is probably related to the inefficient methods of searching the visual preparation space, e.g., to chaotic eye movement activity. We have shown that a meandering horizontal search (based on horizontal saccades) is an effective search pattern as confirmed by the results of qualitative analysis. The so-called meander method allowed for the obtaining of correct results in microscopic haematological analysis of preparations. It is used to determine the composition of leukocytes in blood smear [46]. The results obtained in our research show that the above-mentioned search pattern is used in the analysis of parasitological preparations.

Using statistical analysis, we have shown that the factor that significantly differentiates the search patterns among persons with the highest and lowest correct diagnosis ratio is the number of horizontal saccades, which was statistically significantly higher in persons who achieved better results. A saccade is a movement of the eyeballs that shifts attention to a new area more than two degrees away. Saccades are thus sight shifts that normally place the line of sight on the desired target with a single smooth movement [47]. In other words, a saccade is a fast, stepwise shift of the line of sight to those points of the visual environment where the information needed for the current cognitive task can be found. Saccade movements are activated in response to the need to immediately change the sight direction. Saccade eyeball movements are an important behavioural component during exploration and spatial organisation of the sensory environment [48]. Our research has shown that horizontal saccades discriminated the subject with different results.

Although research indicates that frequent transitions are an indicator of inefficient scanning accompanied by extensive search, the effect of changing the meaning of this phenomenon by certain processes, e.g., in the form of expert knowledge, may occur here [23,49,50]. It seems that, in our research, there was an effect comparable to the phenomenon observed in the research, where the bottom-up effect, resulting from the specific properties of the object, of eye control was different when the subjects viewed images from their specialist field [49]. Importantly, this is indirectly related to the results of the research, which found that questions that require “searching” for answers in long-term memory, that is, require its high involvement, lead to high results of the oculomotor index measured by the number of saccades per second [51]. Oculomotor activity in this respect is associated with the activation of additional cognitive resources in order to search long-term memory to find the appropriate information (equivalent). Spatial saccades simultaneously reduce excessive cognitive load, which may explain the greater accuracy of diagnoses (saccades are then a kind of “key” to the storehouse of knowledge about parasites) [52].

The results of our studies fit into the global discussion on parasitology education. Eye tracking allows the examined person to accurately trace the microscopic image scanning path. The EMMEs method can be a useful model that constitutes an innovative element in teaching microscopic image analysis. Implementation of eye tracking in parasitology education may facilitate the learning of correct pathways in searching microscopic images (correct eye movement), which, in the future, will facilitate the work of medical and laboratory diagnosticians and reduce the risk of incorrect and incomplete diagnoses. Our studies allowed us to conclude that the EMMEs method can be used for assessing how a specimen has been analysed. They have shown that eye tracking allows for the assessment and verification of the work of students and professional laboratory diagnosticians. The results of our studies have also shown that the EMMEs method can be widely used in virtual microscopy. Students, young inexperienced employees and people who have not worked in their profession can learn by analysing and tracking saccadic eye movements of specialists, experts and experienced employees generated in images and videos obtained through eye tracking [15].

The results of our research indicate that practice is highly important in medical parasitology. Therefore, students on various medical courses, during their education using virtual methods in which microscopic specimens are used, should be able to acquire proper skills in analysing microscopic specimens. The use of teaching materials prepared by specialists and experts using the eye tracking method during virtual classes will significantly increase the chance of developing the appropriate microscopy skills.

Incorrectly performed parasitological diagnostics can be the cause of misdiagnosis and thus can have a direct impact on the health or even life of the patient. The results of our research are also indicative of the necessity of life-long learning and education intended not only for students, graduates, and people who have had a professional break, but also for professionally active experts.

In the future, we will consider conducting research in other methodological models, e.g., in an experimental model with two or more comparative groups, and the use of advanced data analysis methods based on machine learning and even artificial intelligence algorithms [18,19,20,21].

This is the very idea behind lifelong learning: “all learning activity is undertaken throughout life, to improve knowledge, skills and competences within a personal, civic, social and/or employment-related perspective” [53]. The eye tracking tool can also be used to create virtual online training courses.

It seems that the virtual exchange between specialists and experts of teaching materials obtained through eye tracking will allow for the exchange of experiences in various medical centres.

## 5. Conclusions

Education materials prepared with the use of eye tracking technology by specialists and experts can be used to teach practical skills in microscopic image analysis. The eye tracking method would allow students, young medics and people who did not work in their profession after their studies to learn the correct manner of scanning and analysing microscopic specimens. The analysis of teaching materials (images and videos) generated by the use of eyetracking, prepared by experienced professionals in the field of parasitology, can be used during various training, simulations and courses in medical parasitology, including virtual training. The EMMEs method can be used in the parasitology education of undergraduate, graduate and postgraduate students. The creation of appropriate computer programs will additionally expand the possibilities of using eye tracking technology in parasitology diagnostics. The eye tracking method would allow for the elimination of errors in microscopic diagnostics in the future.

## Figures and Tables

**Table 1:** Significance of the differences between the number of correct/incorrect parasitological diagnoses (the χ^2^ Pearson test was used to compare the distribution of the answers given by the study participants).

Slides	Experts A		Experts B		Students		χ^2^ Pearson	*p*
	COR	INCOR	COR	INCOR	COR	INCOR		
*Hymenolepis diminuta*	43.75	56.25	0	100	14.29	85.71	9.777 *	0.007 *
Artefacts	43.75	56.25	30	70	64.29	35.71	5.280	0.071
*Trichuris trichiura*	100	0	20	80	94.64	5.36	43.780 *	0.0000 *
*Enterobius vermicularis*	62.5	37.5	10	90	76.79	23.21	16.929 *	0.0002 *
*Giardia intestinalis* and *Entamoeba* sp.	25	75	0	100	3.57	66.43	9.325 *	0.0094 *
*Iodamoeba bütschlii* and artefacts	18.75	81.25	20	80	7.14	92.86	2.665	0.263

COR—% of correct, INCOR—% of incorrect, * statistically significant at *p* < 0.05.

## Data Availability

The data that support the findings of this study are available from the corresponding author upon reasonable request.

## Supplementary Materials

The following are available online at https://www.mdpi.com/article/10.3390/jcm10132989/s1, Video S1: An example scanning paths on the analysis of preparation C—correct diagnosis, Video S2: An example scanning paths on the analysis of preparation C—incorrect diagnosis, Video S3: An example scanning paths on the analysis of preparation D—correct diagnosis, Video S4: An example scanning paths on the analysis of preparation D—incorrect diagnosis, Table S1: The results of the analysis of the significance of differences in the values of the analyzed parameters in the study groups with extreme survey results (U Mann-Whitney test), Figure S1: Distribution of correct and incorrect diagnoses in groups, Figure S2: Descriptive statistics of the “x Ratio” variable in groups with extreme test results (Low—lowest scores, High—highest scores), Figure S3: Descriptive statistics of the “Time of display” variable in groups with extreme test results (Low—lowest scores, High- highest scores), Figure S4: Descriptive statistics of the “Time of questionnaire display” variable in groups with extreme test results (Low—lowest scores, High—highest scores), Figure S5: Descriptive statistics of the “Number of fixation” variable in groups with extreme test results (Low—lowest scores, High—highest scores), Figure S6: Descriptive statistics of the “Mean time of fixation” variable in groups with extreme test results (Low—lowest scores, High—highest scores), Figure S7: Descriptive statistics of the “Number of sacades” variable in groups with extreme test results (Low—lowest scores, High—highest scores), Figure S8: Descriptive statistics of the “Mean time of sacades” variable in groups with extreme test results (Low—lowest scores, High—highest scores), Figure S9: Descriptive statistics of the “Amplitude of sacades” variable in groups with extreme test results (Low—lowest scores, High—highest scores), Figure S10: Descriptive statistics of the “Horizontal sacades” variable in groups with extreme test results (Low—lowest scores, High—highest scores), Figure S11: Descriptive statistics of the “Vertical sacades” variable in groups with extreme test results (Low—lowest scores, High—highest scores), Figure S12: Descriptive statistics of the “Diagonal sacades” variable in groups with extreme test results (Low—lowest scores, High—highest scores), Figure S13: Scanning paths based on the analysis of preparation C—*Trichuris trichiura* for participants who obtained the highest scores, Figure S14: Scanning paths based on the analysis of preparation C—*Trichuris trichiura* for participants who obtained the average scores, Figure S15: Scanning paths based on the analysis of preparation C—*Trichuris trichiura* for participants who obtained the lowest scores, Figure S16: Scanning paths based on the analysis of preparation D—*Enterobius vermicularis* for participants who obtained the highest scores, Figure S17: Scanning paths based on the analysis of preparation D—*Enterobius vermicularis* for participants who obtained the average scores, Figure S18: Scanning paths based on the analysis of preparation D—*Enterobius vermicularis* for participants who obtained the lowest scores, Figure S19: Heat map based on the analysis of preparation A—*Hymenolepis diminuta*, Figure S20: Heat map based on the analysis of preparation B, Figure S21: Heat map based on the analysis of preparation E—*Entamoeba* sp. and Giardia intestinalis, Figure S22: Heat map based on the analysis of preparation F—*Iodamoeba bütschlii* and artefacts.

## References

[1] Brunyé T.T., Drew T., Weaver D.L., Elmore J.G. (2019). A review of eye tracking for understanding and improving diagnostic interpretation. Cogn. Res. Princ. Implic..

[2] Jarodzka H., Holmqvist K., Gruber H. (2017). Eye tracking in Educational Science: Theoretical frameworks and research agendas. J. Eye Mov. Res..

[3] Kok E.M., Jarodzka H. (2017). Before your very eyes: The value and limitations of eye tracking in medical education. Med. Educ..

[4] Ashraf H., Sodergren M.H., Merali N., Mylonas G., Singh H., Darzi A. (2018). Eye-tracking technology in medical education: A systematic review. Med. Teach..

[5] Jaarsma T., Jarodzka H., Nap M., Van Merriénboer J.J.G., Boshuizen H.P.A. (2014). Expertise under the microscope: Processing histopathological slides. Med. Educ..

[6] Nielsona J.A., Mamidala R.N., Khan J. (2013). In-situ eye-tracking of emergency physician result review. Stud. Health Technol. Inform..

[7] Ahmidi N., Hager G.D., Ishii L., Fichtinger G., Gallia G.L., Ishii M. (2010). Surgical task and skill classification from eye tracking and tool motion in minimally invasive surgery. MICCAI 2010: Medical Image Computing and Computer-Assisted Intervention, Proceedings of the International Conference on Medical Image Computing and Computer-Assisted Intervention, Beijing, China, 20–24 September 2010.

[8] Law B., Atkins M.S., Kirkpatrick A.E., Lomax A.J. (2004). Eye gaze patterns differentiate novice and experts in a virtual laparoscopic surgery training environment. Proceedings of the 2004 Eye Tracking Research and Applications Symposium.

[9] Tien G., Atkins M.S., Zheng B., Swindells C. Measuring situation awareness of surgeons in laparoscopic training. Proceedings of the Eye Tracking Research and Applications ETRA.

[10] Zheng B., Tien G., Atkins S.M., Swindells C., Tanin H., Meneghetti A., Qayumi K.A., Panton O.N.M. (2011). Surgeon’s vigilance in the operating room. Am. J. Surg..

[11] Forsman J., Anani N., Eghdam A., Falkenhav M., Koch S. (2013). Integrated information visualization to support decision making for use of antibiotics in intensive care: Design and usability evaluation. Inform. Health Soc. Care.

[12] King A.J., Hochheiser H., Visweswaran S., Clermont G., Cooper G.F. (2017). Eye-tracking for clinical decision support: A method to capture automatically what physicians are viewing in the EMR. AMIA Jt. Summits Transl. Sci. Proc..

[13] King A.J., Cooper G.F., Clermont G., Hochheiser H., Hauskrecht M., Sittig D.F., Visweswaran S. (2020). Leveraging Eye Tracking to Prioritize Relevant Medical Record Data: Comparative Machine Learning Study. J. Med. Internet Res..

[14] Jarodzka H., Scheiter K., Gerjets P., van Gog T. (2010). In the eyes of the beholder: How experts and novices interpret dynamic stimuli. Learn. Instr..

[15] Jarodzka H., van Gog T., Dorr M., Scheiter K., Gerjets P. (2013). Learning to see: Guiding students’ attention via a Model’s eye movements fosters learning. Learn. Instr..

[16] Jabbar A., Gauci C.G., Anstead C.A. (2021). Parasitology Education Before and After the COVID-19 Pandemic. Trends Parasitol..

[17] Ahmed H., Allaf M., Elghazaly H. (2020). COVID-19 and medical education. Lancet Infect. Dis..

[18] Dewhurst R., Nyström M., Jarodzka H., Foulsham T., Johansson R., Holmqvist K. (2012). It depends on how you look at it: Scanpath comparison in multiple dimensions with MultiMatch, a vector-based approach. Behav. Res. Methods.

[19] Fuhl W., Castner N., Kübler T., Lotz A., Rosenstiel W., Kasneci E. Ferns for area of interest free scanpath classification. Proceedings of the 11th ACM Symposium on Eye Tracking Research & Applications, ETRA ’19.

[20] Kübler T.C., Rothe C., Schiefer U., Rosenstiel W., Kasneci E. (2017). SubsMatch 2.0: Scanpath comparison and classification based on subsequence frequencies. Behav. Res. Methods.

[21] Fuhl W., Bozkir E., Hosp B., Castner N., Geisler D., Santini T.C., Kasneci E. Encodji. Proceedings of the 11th ACM Symposium on Eye Tracking Research & Applications, ETRA ’19.

[22] Tobii A.B. Tobii Studio User’s Manual. Version 3.4.5. https://www.tobiipro.com/siteassets/tobii-pro/user-manuals/tobii-pro-studio-user-manual.pdf.

[23] Goldberg J.H., Kotval X.P. (1999). Computer interface evaluation using eye movements: Methods and constructs. Int. J. Ind. Ergon..

[24] Poole A., Ball L.J., Ghaoui C. (2006). Eye Tracking in HCI and Usability Research. Encyclopedia of Human Computer Interaction.

[25] Poole A., Ball L.J., Phillips P., Fincher S., Markopolous P., Moore D., Ruddle R. (2004). In search of salience: A response time and eye movement analysis of bookmark recognition. People and Computers XVIII-Design for Life: Proceedings of HCI.

[26] Leigh R.J., Zee D.S. (1999). The Neurology of Eye Movements.

[27] Krauzlis R.J. (2008). Eye Movements. Fundamental Neuroscience.

[28] Wang R., Chen L., Ayesh A., Shell J., Solheim I. Gaze-Based Assessment of Dyslexic Students’ Motivation within an E-Learning Environment. Proceedings of the 2019 IEEE SmartWorld, Ubiquitous Intelligence & Computing, Advanced & Trusted Computing, Scalable Computing & Communications, Cloud & Big Data Computing, Internet of People and Smart City Innovation (SmartWorld/SCALCOM/UIC/ATC/CBDCom/IOP/SCI).

[29] Hofstetter H., Griffin J.R., Berman M.S., Everson R.W. (2000). Dictionary of Visual Science and Related Clinical Terms.

[30] Noton D., Stark L.W. (1971). Scan paths in eye movements during pattern perception. Science.

[31] Noton D., Stark L.W. (1971). Eye movements and visual perception. Sci. Am..

[32] Myjak P., Głowniak C., Gołąb E., Jaborowska-Jarmoluk M., Kosik-Bogacka D., Matowicka-Karna J., Nowak P., Pietkiewicz H., Szostakowska B., Wnukowska N. (2011). Standards in the range of laboratory activities in medical parasitology, estimation of their quality and diagnostics value, as well as interpretation and authorization of the tests results (proposals). J. Lab. Diagn..

[33] Tuszyńska-Bogucka W., Kwiatkowski B., Chmielewska M., Dzieńkowski M., Kocki W., Pełka J., Przesmycka N., Bogucki J., Galkowski D. (2020). The effects of interior design on wellness—Eye tracking analysis in determining emotional experience of architectural space. A survey on a group of volunteers from the Lublin Region, Eastern Poland. Ann. Agric. Environ. Med..

[34] Wayne D.B., Green M., Neilson E.G. (2020). Medical education in the time of COVID-19. Sci. Adv..

[35] Jumreornvong O., Yang E., Race J., Appel J. (2020). Telemedicine and Medical Education in the Age of COVID-19. Acad. Med..

[36] Caruso M.C. (2021). Virtual Microscopy and Other Technologies for Teaching Histology during Covid-19. Anat. Sci. Educ..

[37] Rhoads D.D., Mathison B.A., Bishop H.S., Da Silva A.J., Pantanowitz L. (2016). Review of Telemicrobiology. Arch. Pathol. Lab. Med..

[38] Ekeland A.G., Bowes A., Flottorp S. (2010). Effectiveness of telemedicine: A systematic review of reviews. Int. J. Med. Inform..

[39] Pfeiffer C.N., Jabbar A. (2019). Adaptive e-Learning: Emerging Digital Tools for Teaching Parasitology. Trends Parasitol..

[40] Jabbar A., Gasser R.B., Lodge J. (2016). Can New Digital Technologies Support Parasitology Teaching and Learning?. Trends Parasitol..

[41] Jabbar A., Gasser R.B. (2018). Special issue—Learning and teaching of veterinary parasitology. Vet. Parasitol..

[42] Peña-Fernández A., Acosta L., Fenoy S., Magnet A., Izquierdo F., Bornay F.J., Ollero M.D., Hurtado C., Del Aguila C. (2020). Evaluation of a novel digital environment for learning medical parasitology. High. Educ. Pedagog..

[43] Meléndez R.D. (2003). Trends in teaching parasitology: Where to complain?. Trends Parasitol..

[44] Mathison B.A. What Can the CDC Do for You? Telediagnosis and Molecular Technologies for Diagnosing Parasitic Diseases. Proceedings of the 114th ASM General Meeting.

[45] Mathison B.A., Bishop H., Eberhard M.L., Johnston S.P., Long E.K., da Silva A.J. Usefulness of telediagnosis in the identification of tissue parasites: An evaluation based on two years (from 2006–2008) of telediagnosis submissions to the CDC DPDx Project. Proceedings of the 57th American Society of Tropical Medicine and Hygiene Annual Meeting.

[46] Rodak B.F., Fritsma G.A., Doig K. (2007). Hematology—Clinical Principles and Applications.

[47] Keller E.L., Lee B.-T., Lee K.-M. (2008). Frontal eye field signals that may trigger the brainstem saccade generator. Prog. Brain Res..

[48] Wong A.M.F. (2014). Eye Movements; Saccades, Encyclopedia of the Neurological Sciences.

[49] Humphrey K., Underwood G. (2009). Domain knowledge moderates the influence of visual saliency in scene recognition. Br. J. Psychol..

[50] Tatler B.W., Baddeley R.J., Vincent B. (2006). The long and the short of it: Spatial statistics at fixation vary with saccade amplitude and task. Vis. Res..

[51] Micic D., Ehrlichman H., Chen R. (2010). Why do we move our eyes while trying to remember? The relationship between non-visual gaze patterns and memory. Brain Cogn..

[52] Płużyczka M., Warszawski U. (2016). Przestrzenne ruchy sakadowe a pamięć długotrwała [Spatial saccadic movements and long-term memory]. Lingwist. Stosow..

[53] Commision of European Communities (2014). Making a European Area of Lifelong Learning a Reality.

