# Supplementary material for: Eye Tracking—An Innovative Tool in Medical Parasitology
Source: J Clin Med. 2021 Jul 4;10(13):2989. doi: 10.3390/jcm10132989 (PMC8268455; doi:10.3390/jcm10132989)
Supplement: Supplementary file 1 [file jcm-10-02989-s001.zip › Supplementary materials - Qualitative analysis_C.pdf]

Journal of Clinical Medicine

MDPI

Supplementary Materials: Eye tracking – an innovative tool in medical parasitology

Przemysław Kołodziej [1,*], Wioletta Tuszyńska-Bogucka [2], Mariusz Dzieńkowski [3], Jacek Bogucki [4], Janusz Kocki [5], Marek Milosz [3], Marcin Kocki [6], Patrycja Reszka [6], Wojciech Kocki [7] and Anna Bogucka-Kocka [1]

Qualitative analysis - preparation C

Scanning paths based on the analysis of preparation C - *Trichuris trichiura* for participants who obtained the highest scores.

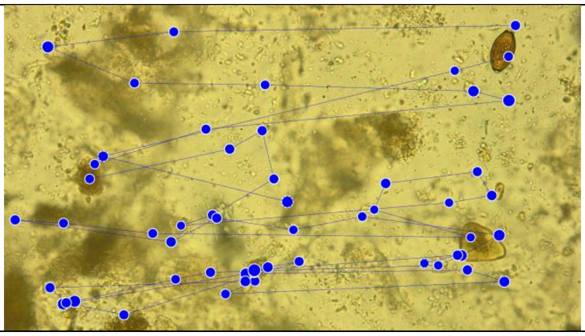	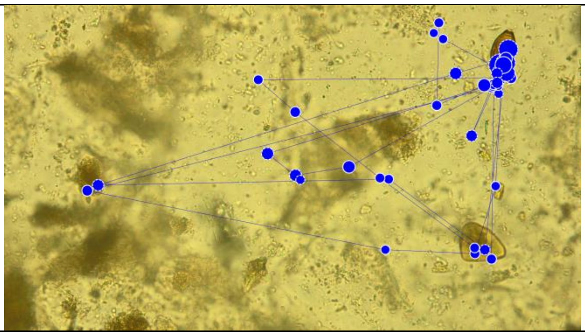
P14	P27
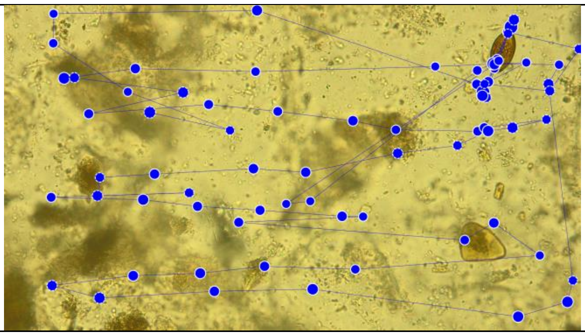	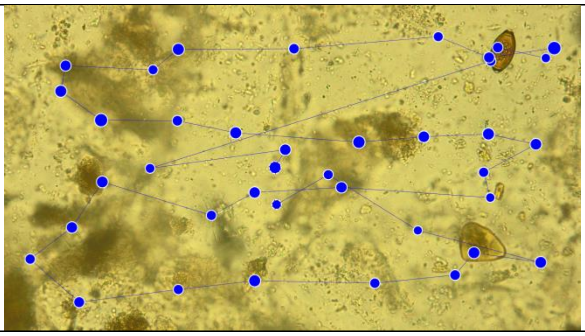
P40	P41

Journal of
Clinical Medicine
MDPI

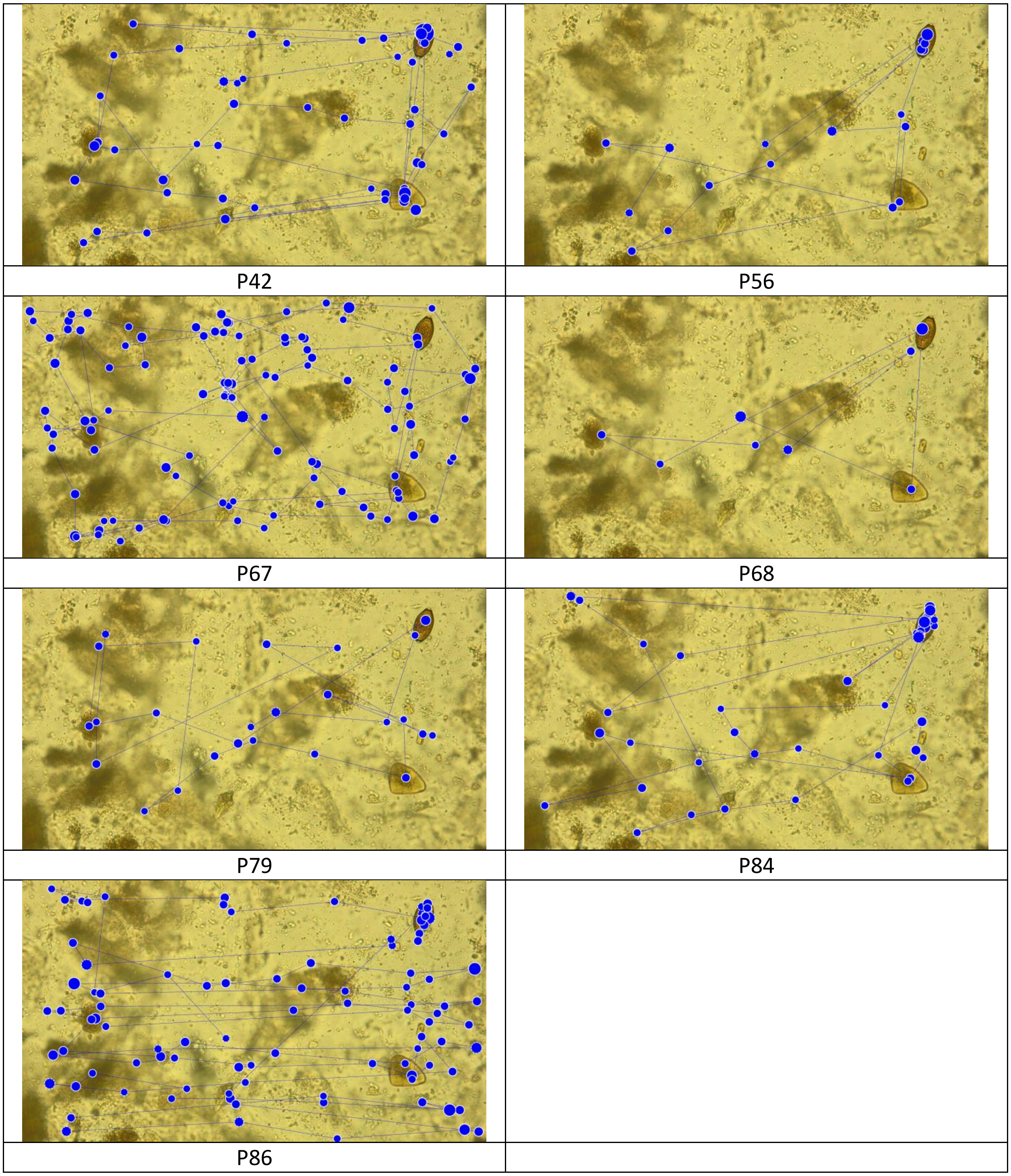

Figure S13. Scanning paths based on the analysis of preparation C - *Trichuris trichiura* for participants who obtained the highest scores. Blue scanning paths - correct diagnosis, red scanning paths – incorrect diagnosis; dot - fixation (eye hold); dot diameter is directly proportional to the time the eye is held; line - saccade (quick shift of eyesight from one point to another).

Journal of
Clinical Medicine

MDPI

Scanning paths based on the analysis of preparation C - *Trichuris trichiura* for participants who obtained the average scores.

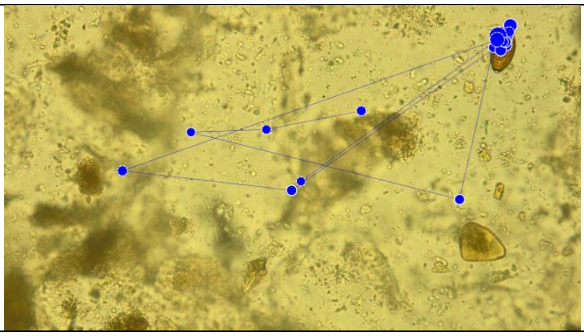	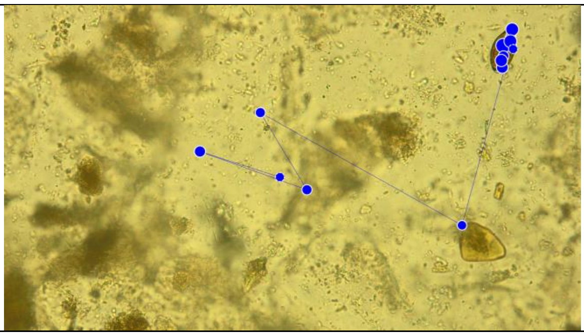
P02	P04
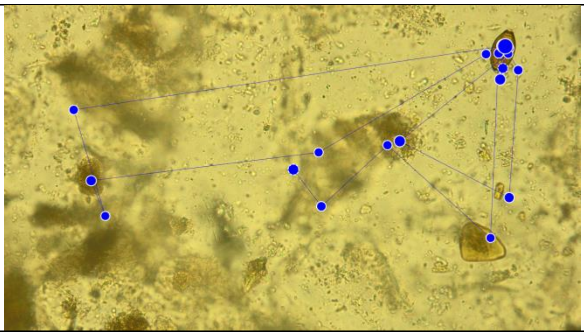	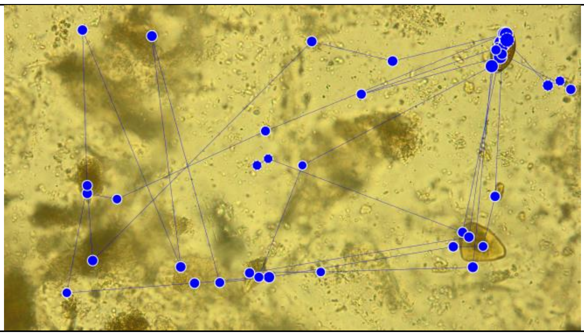
P05	P06
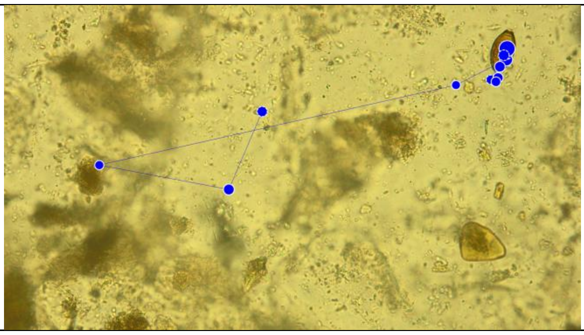	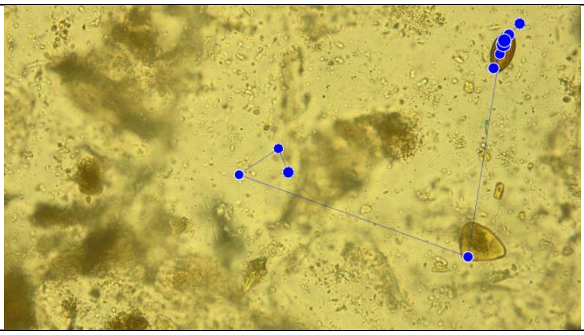
P07	P08
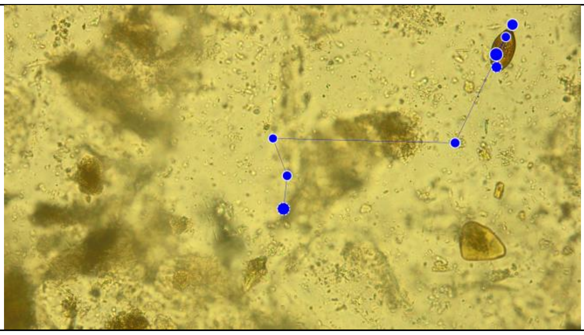	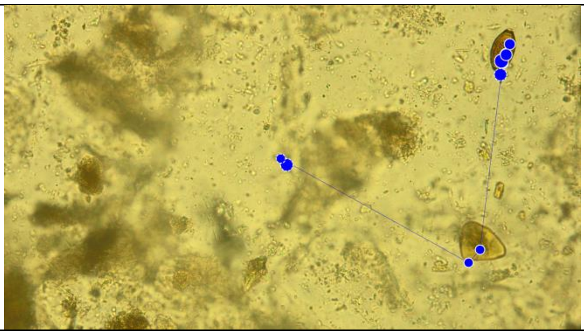
P10	P11

Journal of
Clinical Medicine

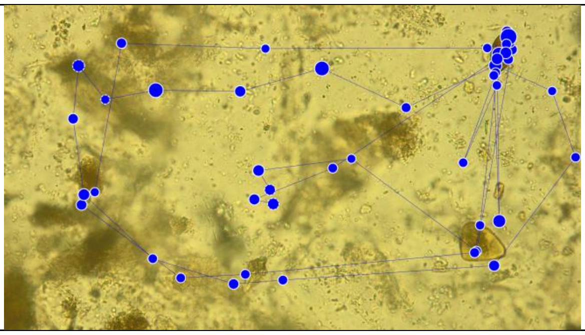

P12

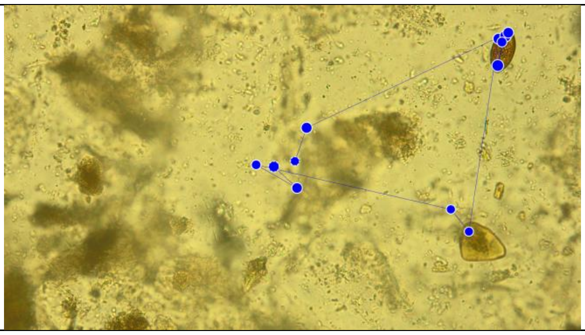

P13

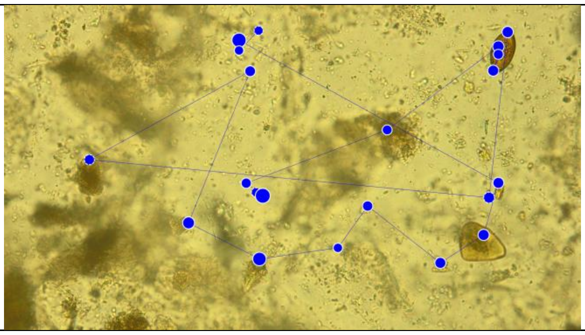

P15

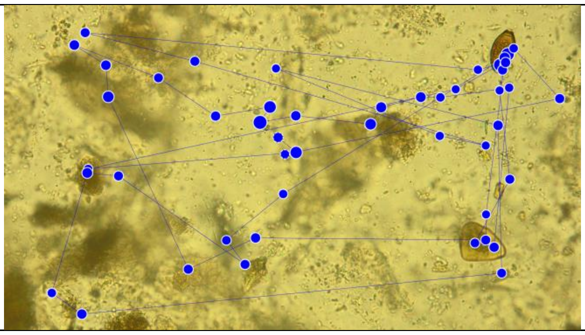

P16

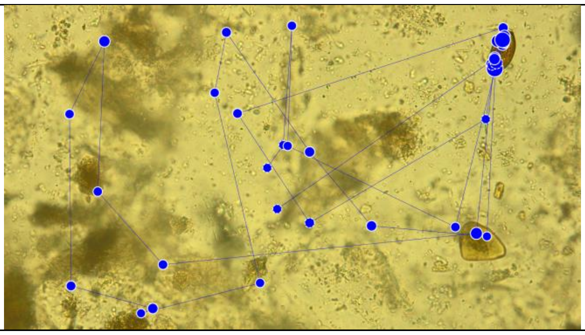

P17

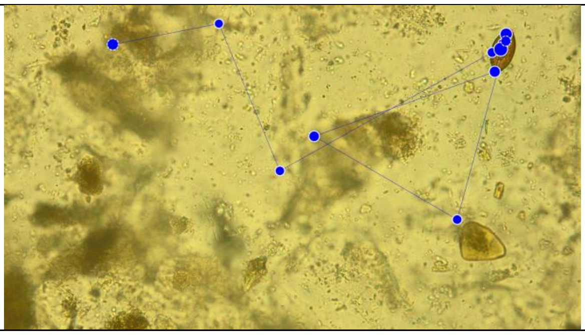

P18

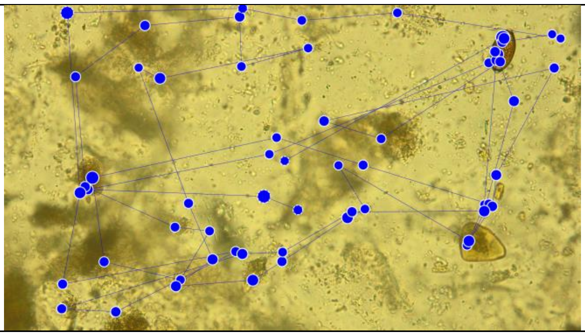

P20

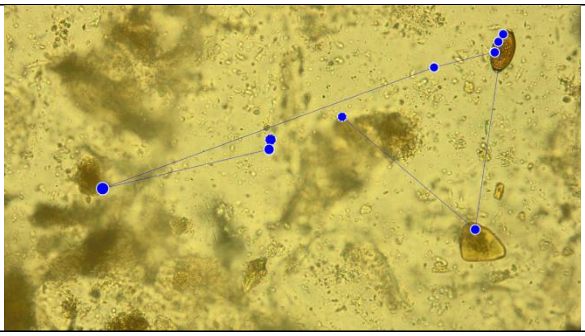

P21

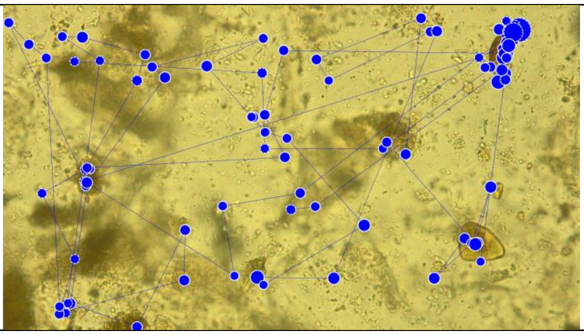
P22

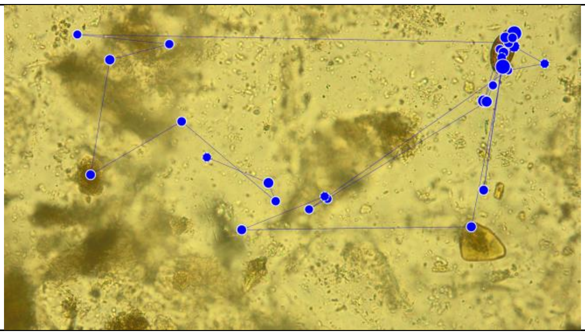
P23

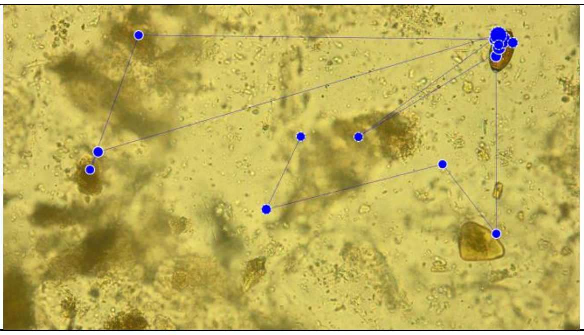
P24

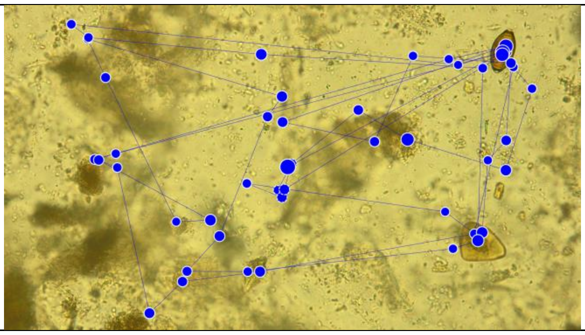
P25

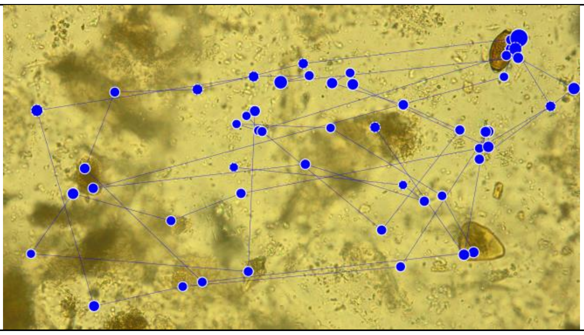
P26

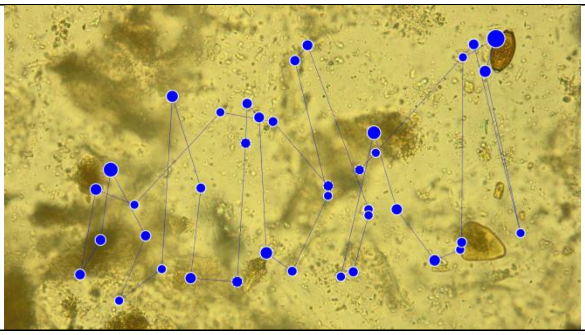
P28

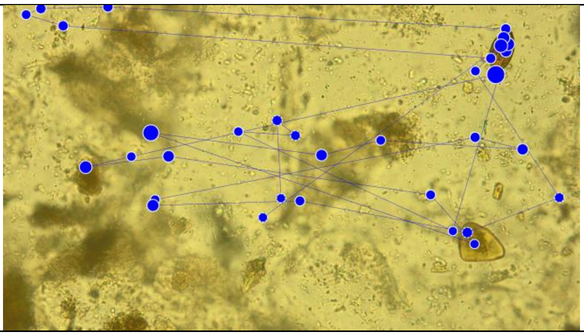
P29

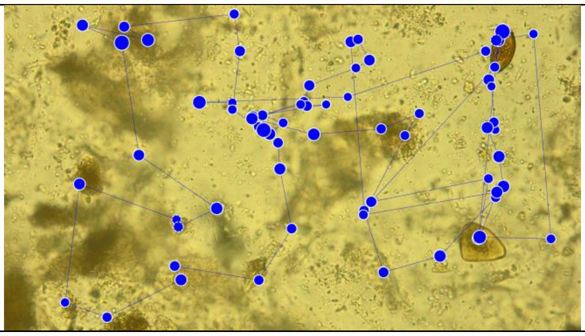
P31

Journal of
Clinical Medicine

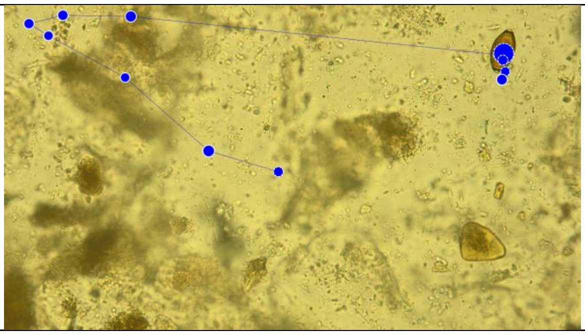	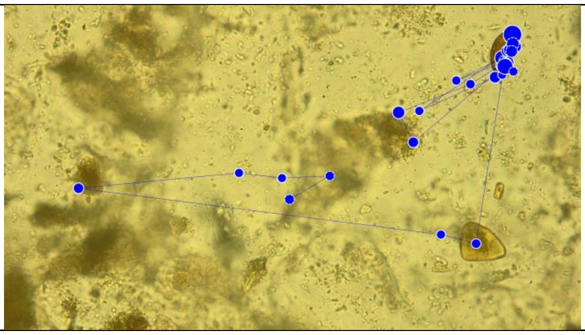
P32	P33
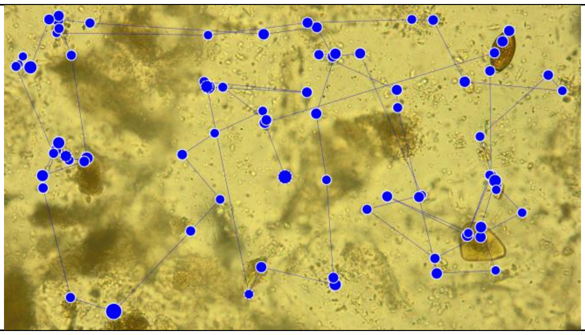	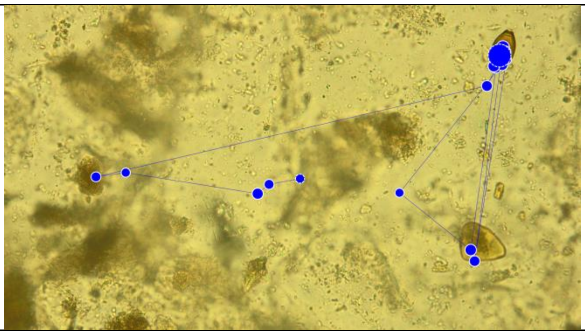
P34	P35
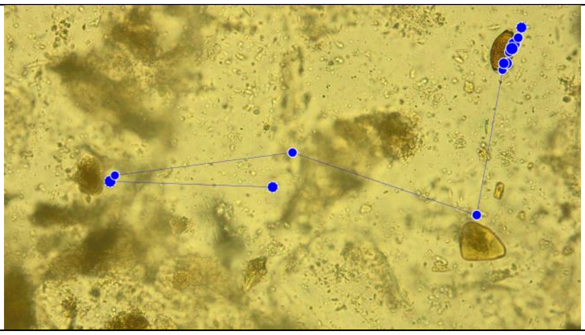	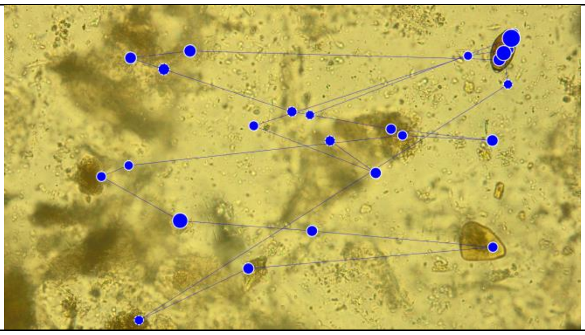
P36	P38
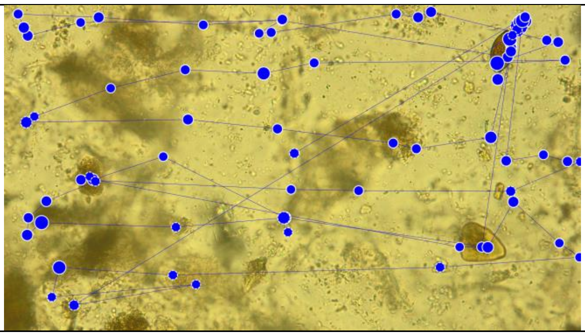	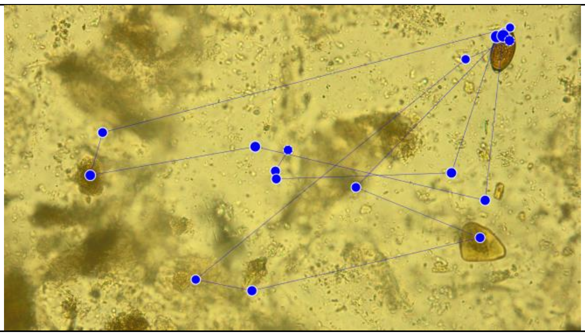
P39	P44

Journal of
Clinical Medicine

MDPI

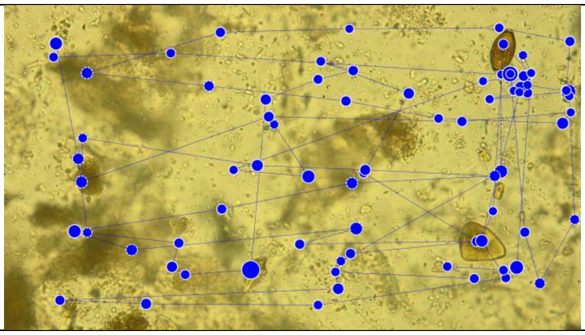	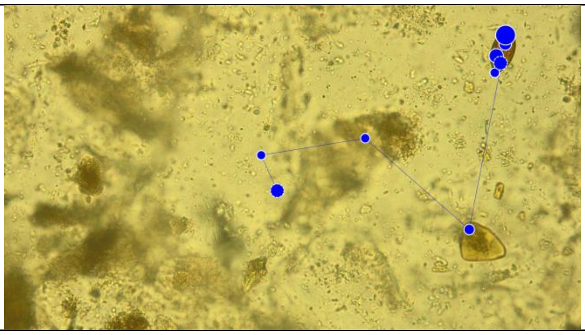
P45	P47
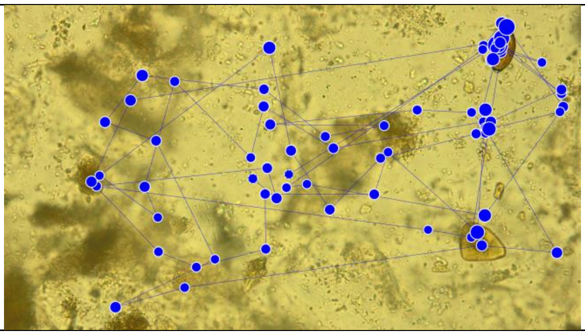	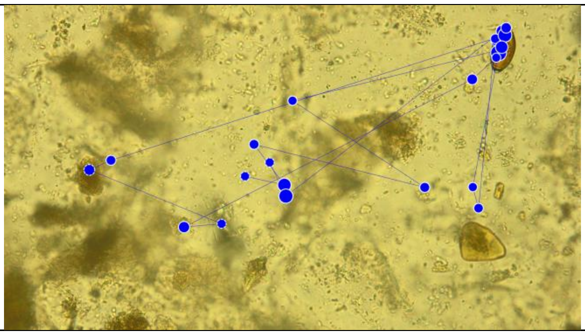
P48	P49
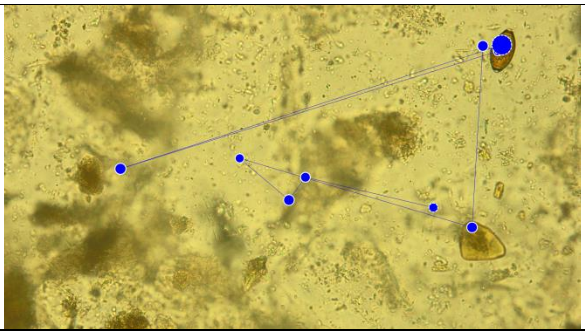	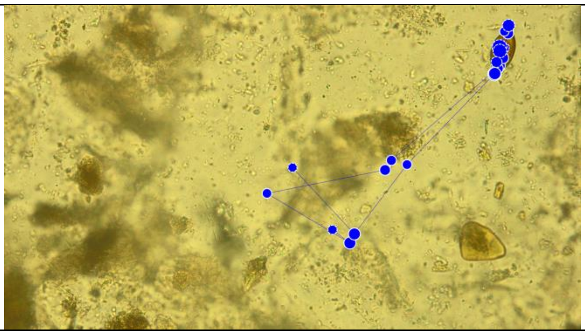
P53	P54
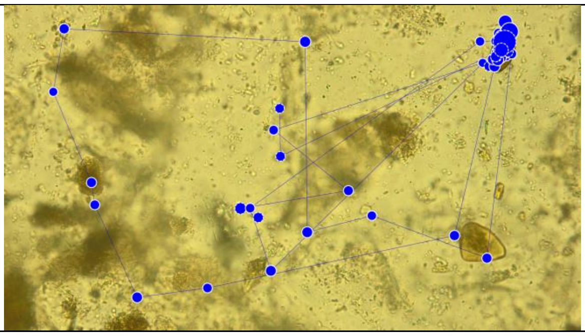	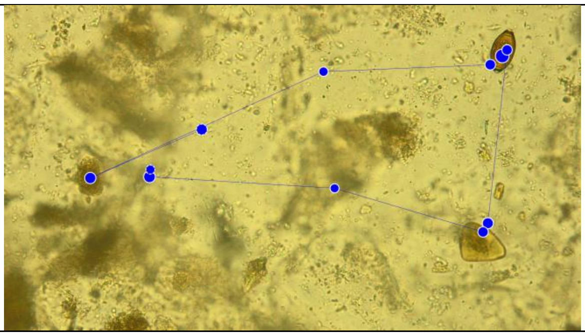
P57	P58

Journal of
Clinical Medicine

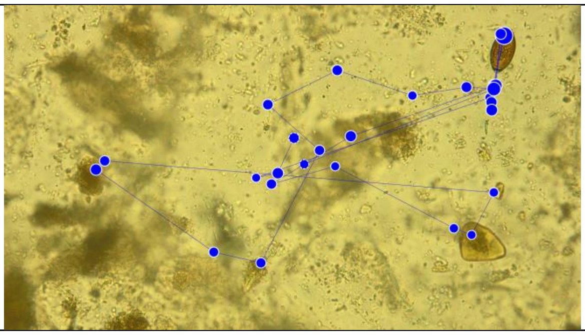
P64

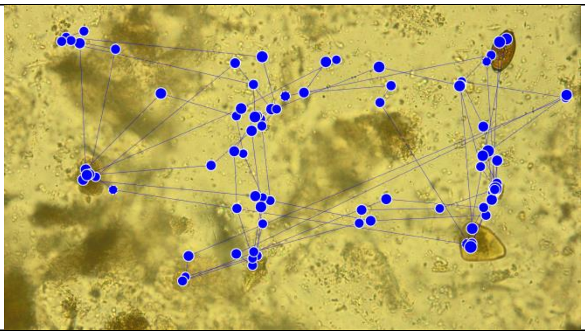
P66

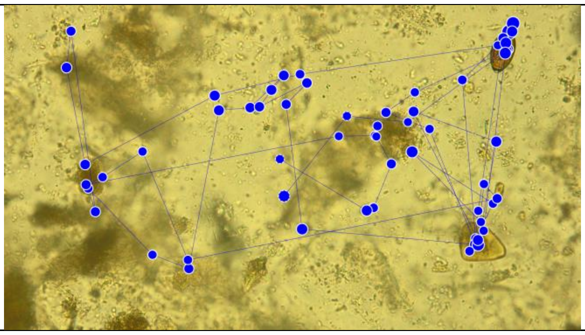
P73

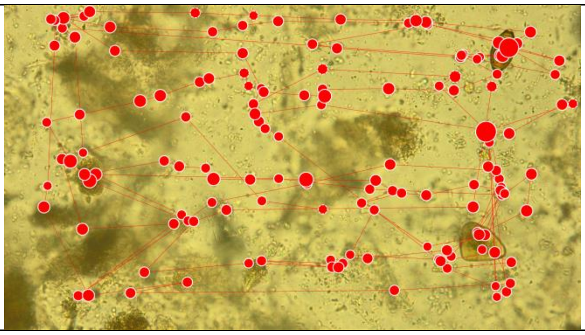
P74

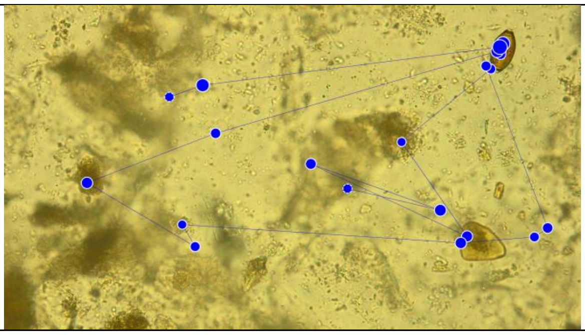
P75

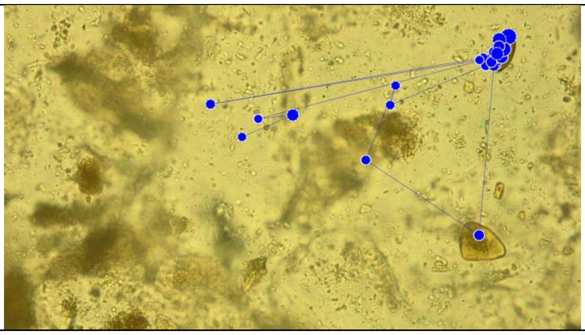
P76

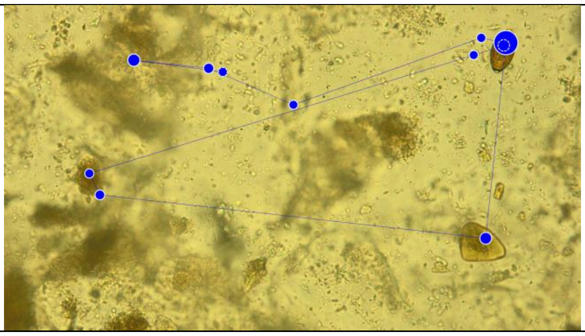
P77

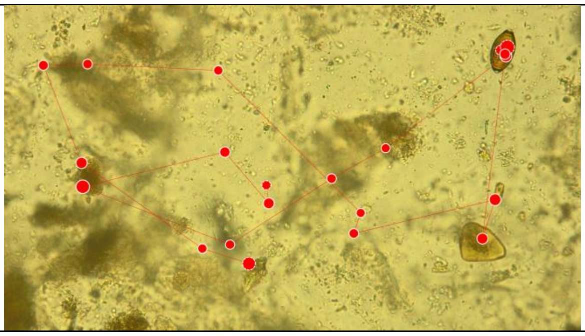
P85

Journal of
Clinical Medicine
MDPI

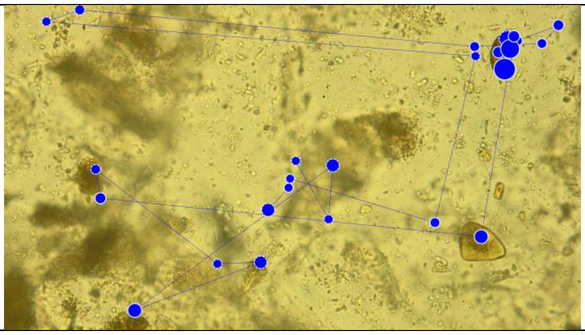	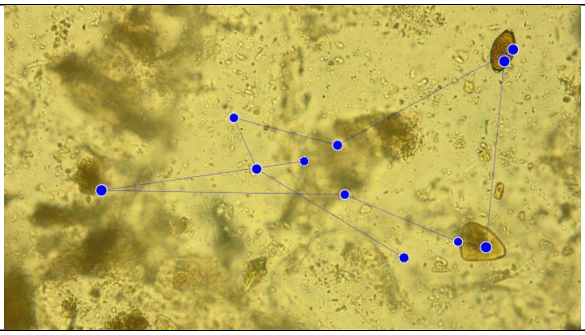
P87	P89
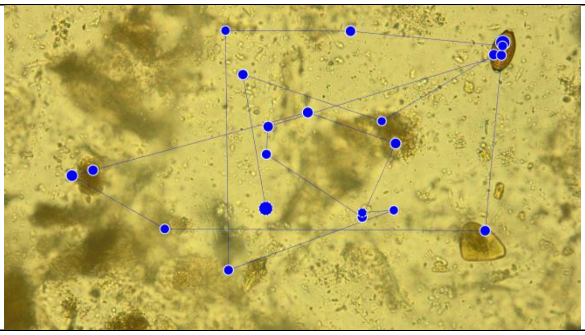	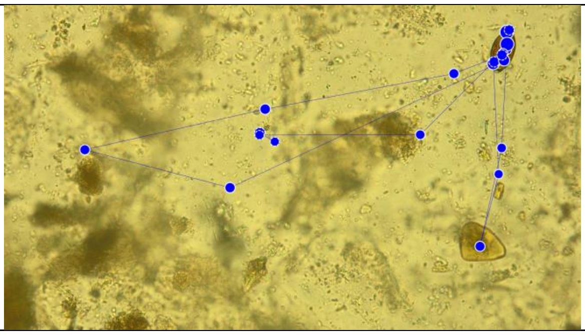
P90	P91

Figure S14. Scanning paths based on the analysis of preparation C - *Trichuris trichiura* for participants who obtained the average scores. Blue scanning paths - correct diagnosis, red scanning paths – incorrect diagnosis; dot - fixation (eye hold); dot diameter is directly proportional to the time the eye is held; line - saccade (quick shift of eyesight from one point to another).

In the included in the supplement - the collection of scan paths, for individual participants who took part in the research, there is no path for participant P1. Despite the fact that all the eye tracking data and the participant's responses were correctly registered, the software did not generate the scan paths due to an unidentified system error.

Scanning paths based on the analysis of preparation C - *Trichuris trichiura* for participants who obtained the lowest scores.

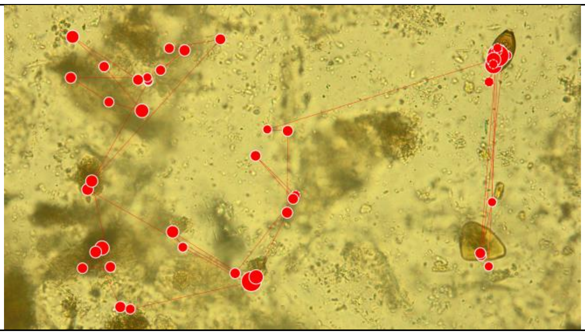	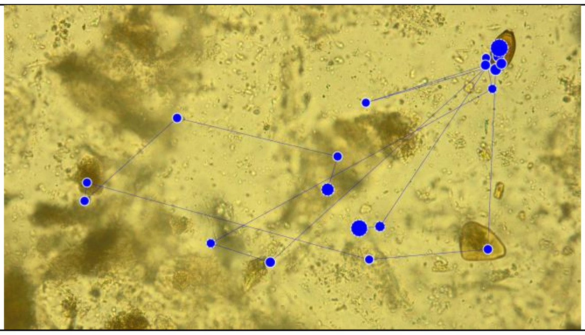
P03	P09

Journal of
Clinical Medicine

MDPI

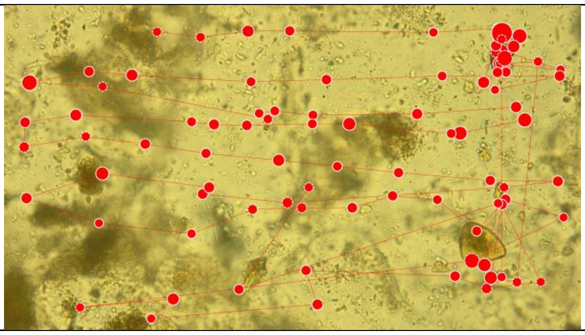

P30

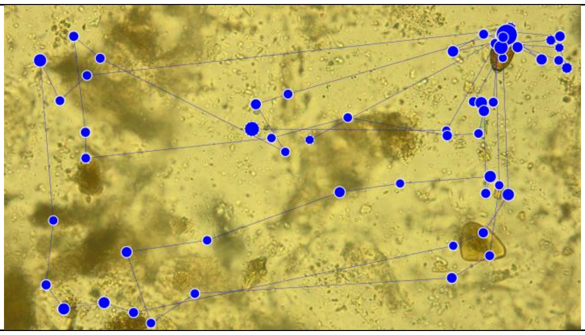

P37

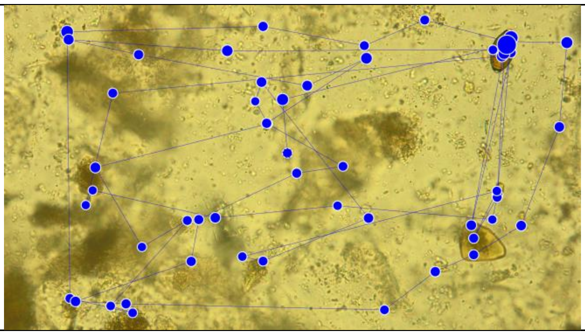

P43

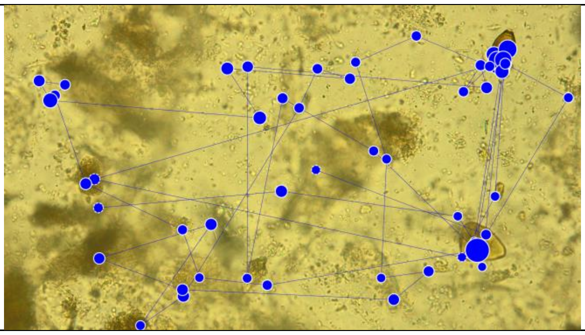

P46

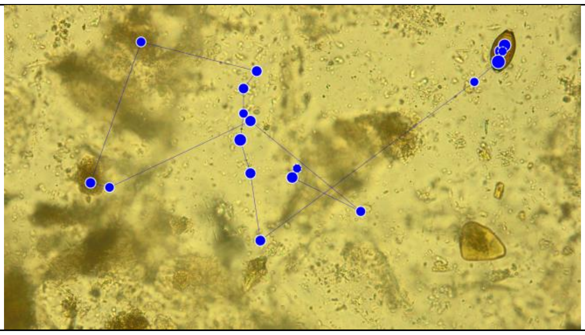

P52

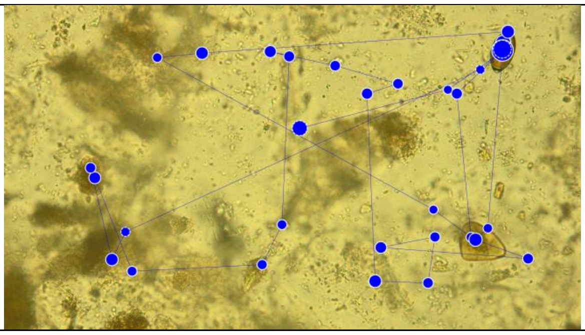

P55

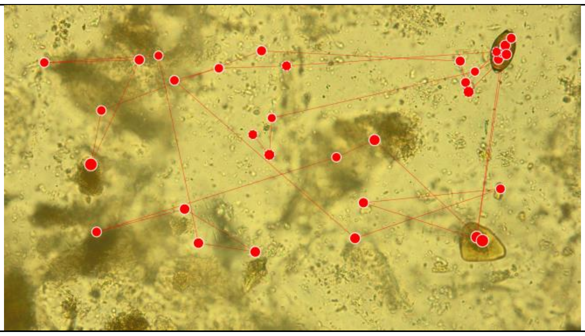

P59

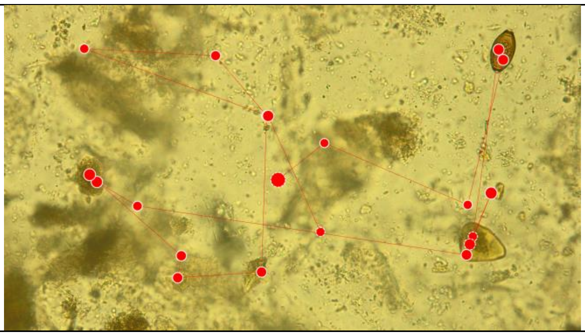

P60

Journal of Clinical Medicine

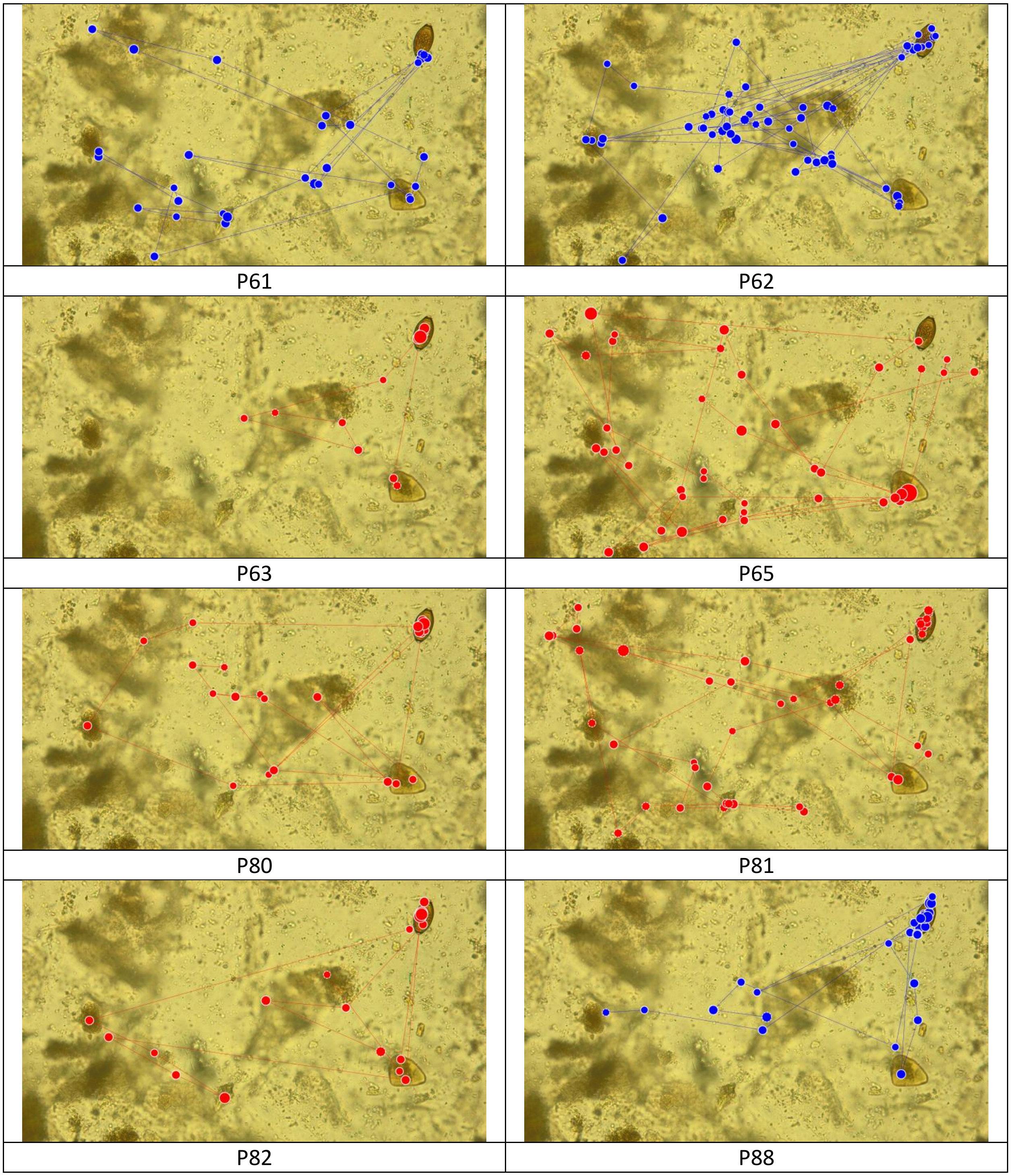

Figure S15. Scanning paths based on the analysis of preparation C - *Trichuris trichiura* for participants who obtained the lowest scores. Blue scanning paths - correct diagnosis, red scanning paths – incorrect diagnosis; dot - fixation (eye hold); dot diameter is directly proportional to the time the eye is held; line - saccade (quick shift of eyesight from one point to another).
