# Supplementary material for: Eye Tracking—An Innovative Tool in Medical Parasitology
Source: J Clin Med. 2021 Jul 4;10(13):2989. doi: 10.3390/jcm10132989 (PMC8268455; doi:10.3390/jcm10132989)
Supplement: Supplementary file 1 [file jcm-10-02989-s001.zip › Supplementary materials - Qualitative analysis_D.pdf]

Journal of
Clinical Medicine

MDPI

Supplementary Materials: Eye tracking – an innovative tool in medical parasitology

Przemysław Kołodziej [1,*], Wioletta Tuszyńska-Bogucka [2], Mariusz Dzieńkowski [3], Jacek Bogucki [4], Janusz Kocki [5], Marek Milosz [3], Marcin Kocki [6], Patrycja Reszka [6], Wojciech Kocki [7] and Anna Bogucka-Kocka [1]

Qualitative analysis - preparation D

Scanning paths based on the analysis of preparation D - *Enterobius vermicularis* for participants who obtained the highest scores

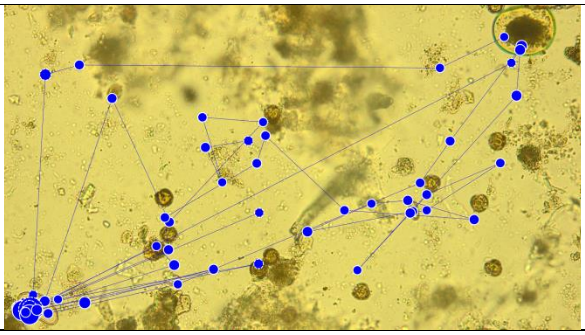	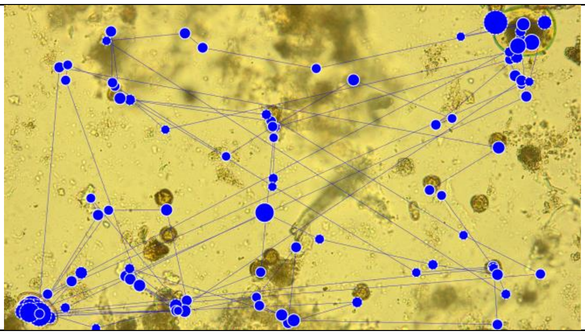
P14	P27
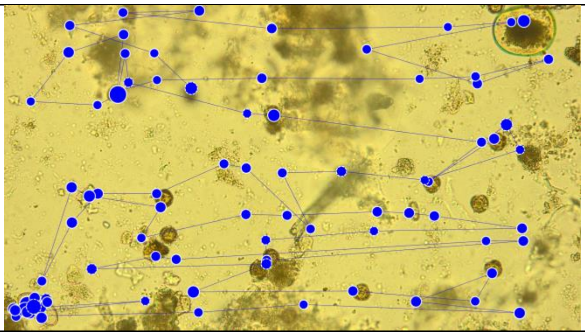	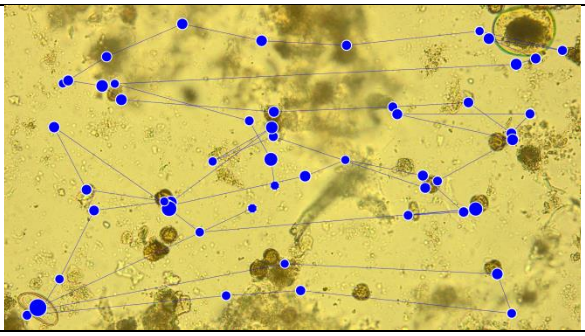
P40	P41

Journal of
Clinical Medicine

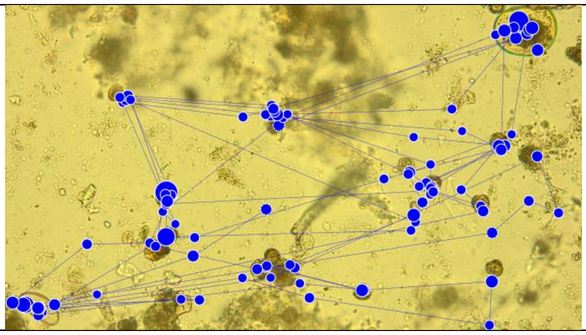	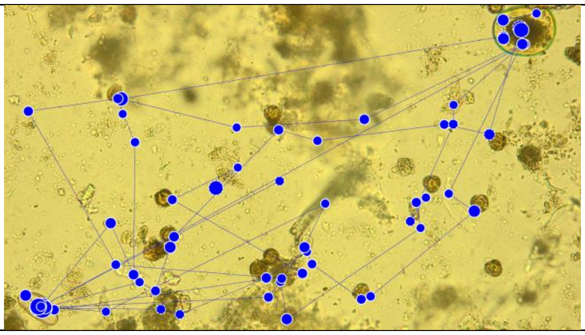
P42	P56
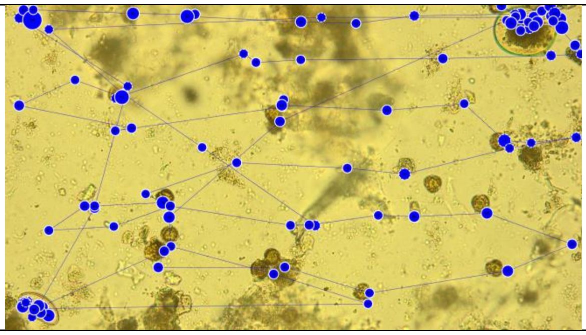	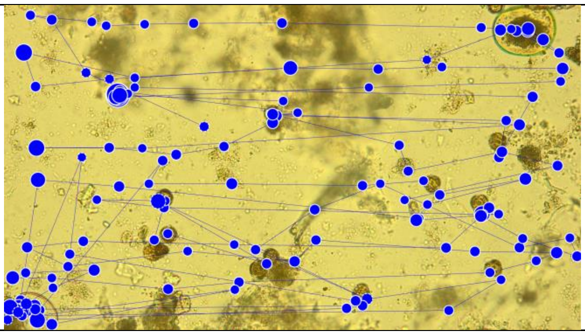
P67	P68
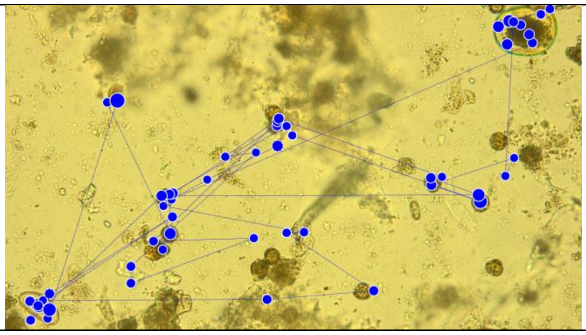	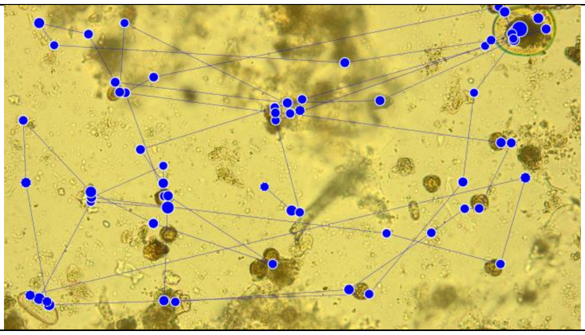
P79	P84
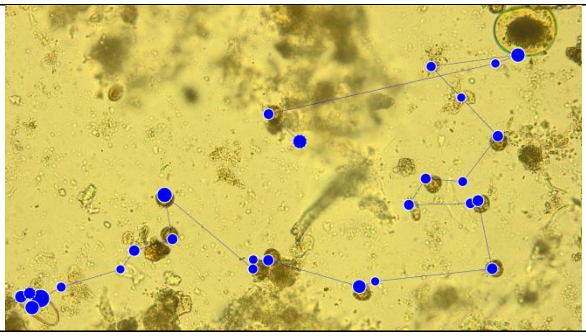	
P86	

Figure S16. Scanning paths based on the analysis of preparation D - *Enterobius vermicularis* for participants who obtained the highest scores. Blue scanning paths - correct diagnosis, red scanning paths – incorrect diagnosis; dot - fixation (eye hold); dot diameter is directly proportional to the time the eye is held; line - saccade (quick shift of eyesight from one point to another).

Journal of
Clinical Medicine

MDPI

Scanning paths based on the analysis of preparation D - Enterobius vermicularis for participants who obtained the average scores.

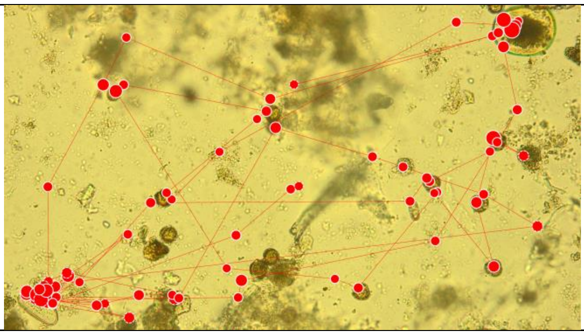	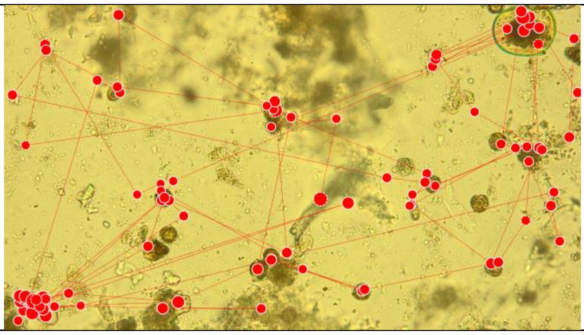
P2	P04
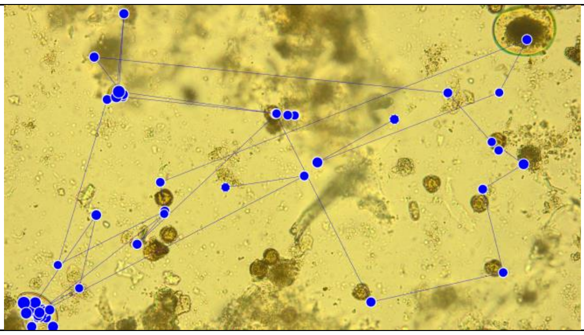	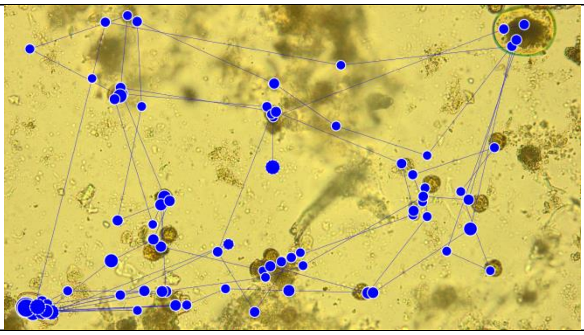
P05	P06
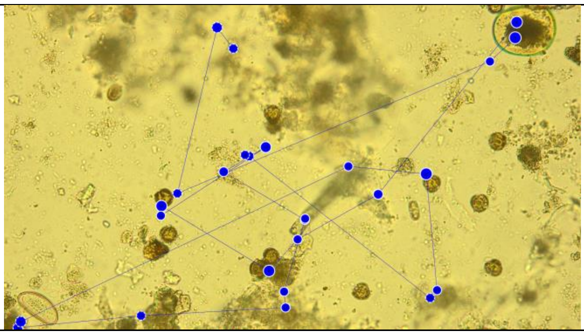	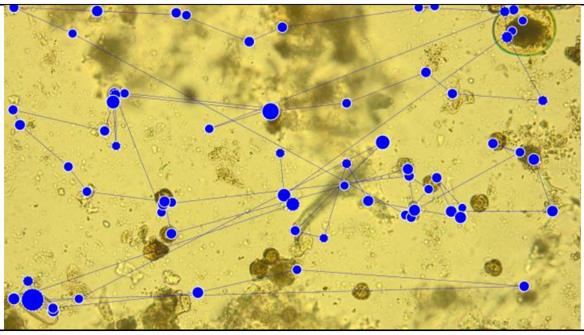
P07	P08
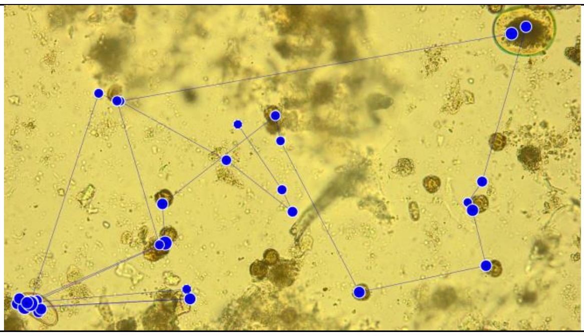	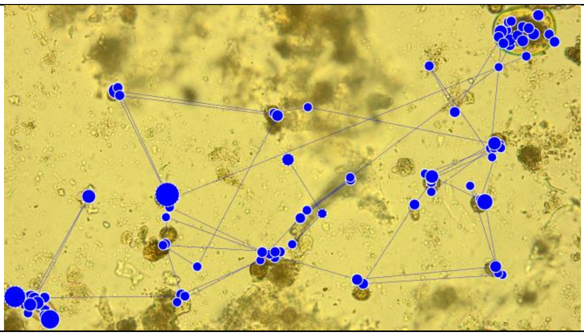
P10	P11

Journal of
Clinical Medicine

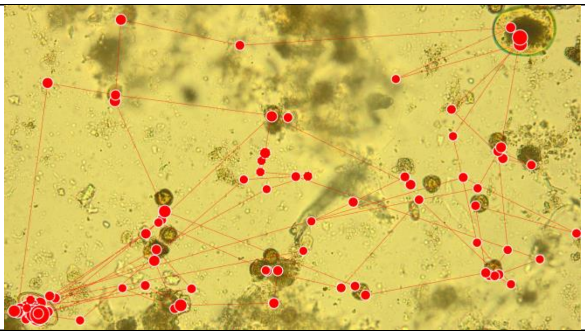	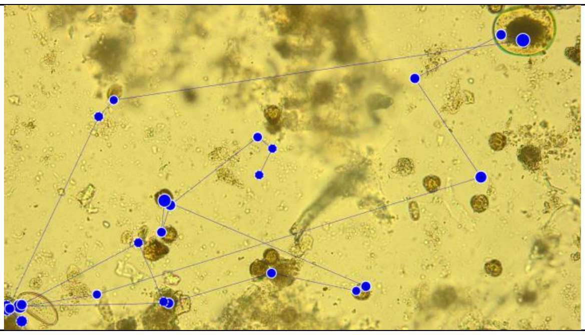
P12	P13
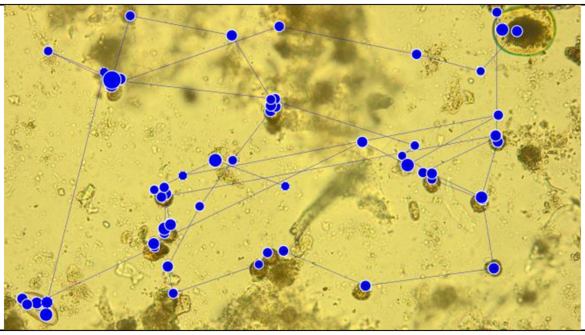	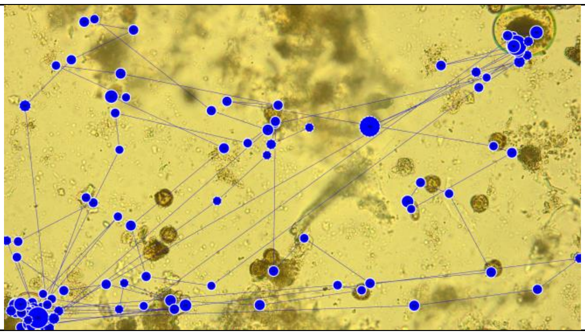
P15	P16
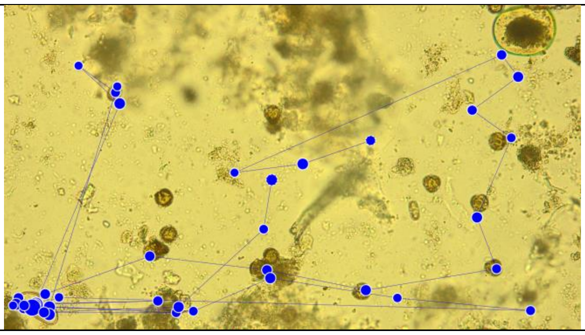	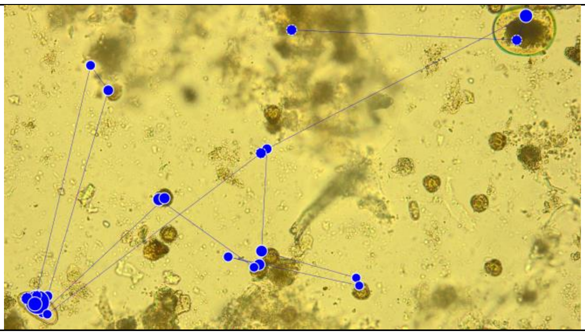
P17	P18
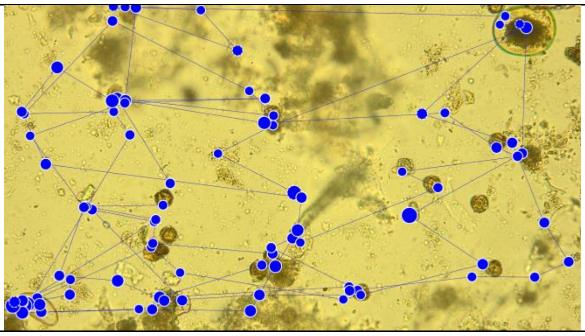	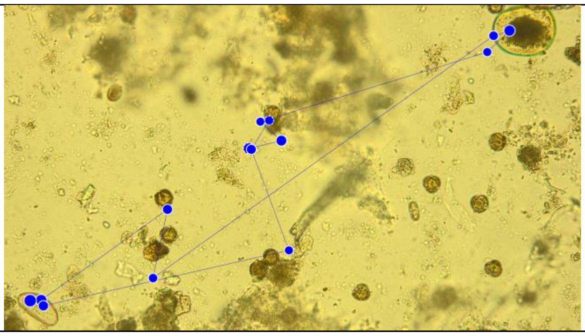
P20	P21

Journal of
Clinical Medicine

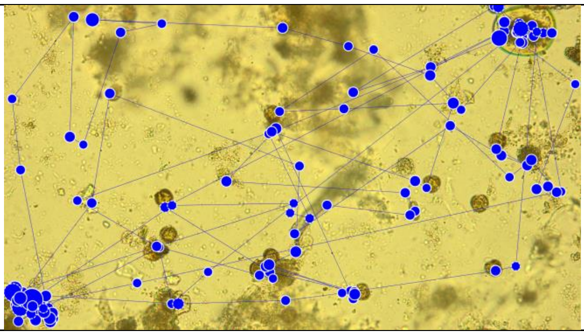	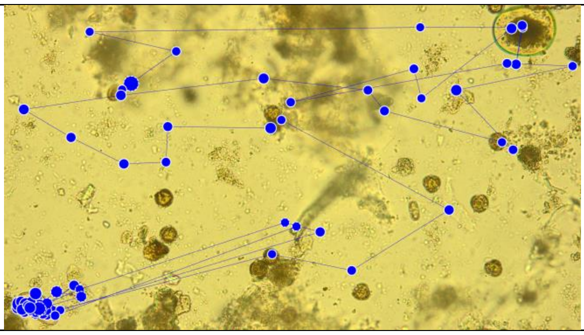
P22	P23
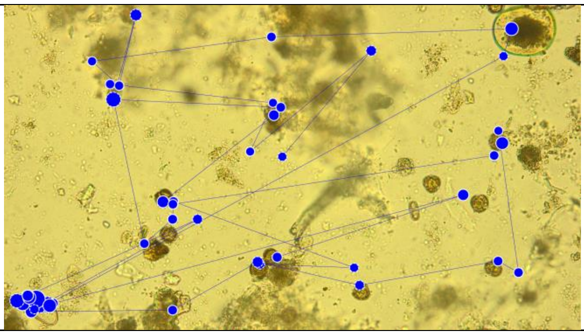	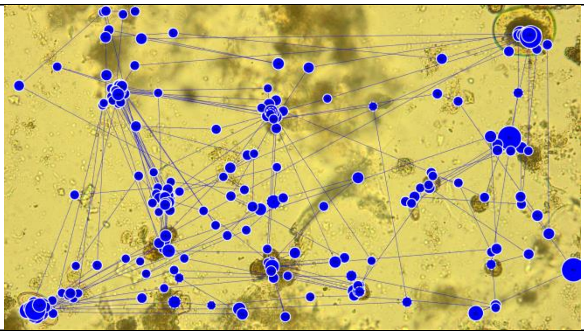
P24	P25
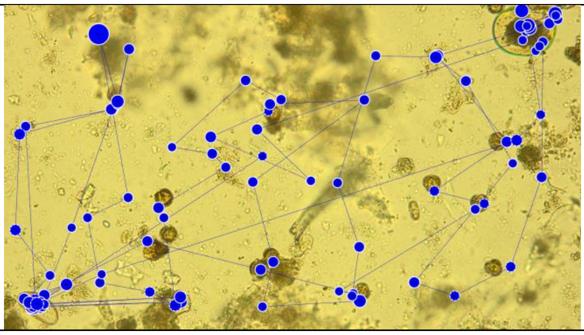	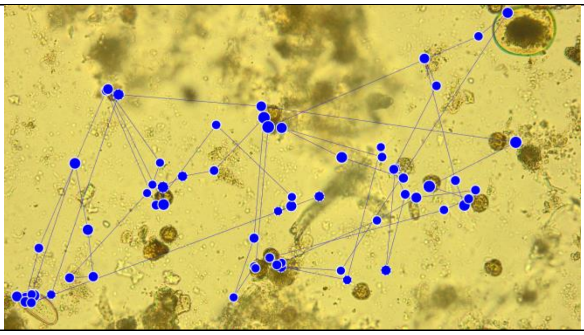
P26	P28
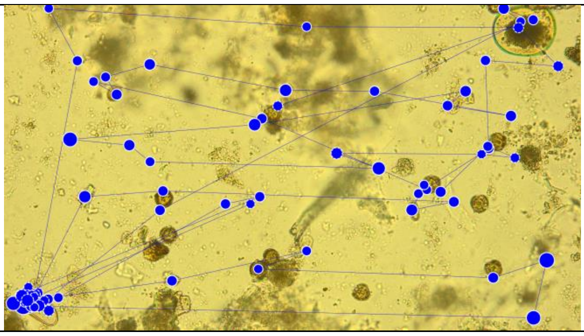	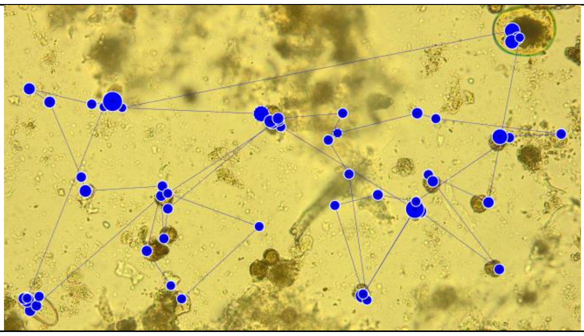
P29	P31

Journal of
Clinical Medicine
MDPI

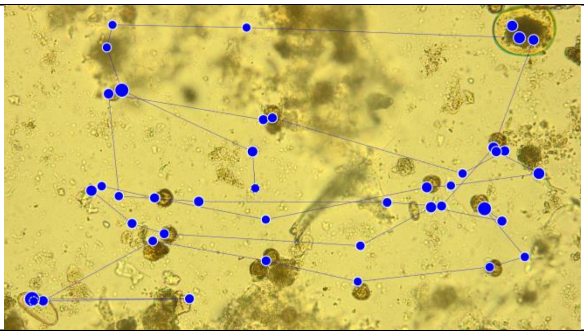

P32

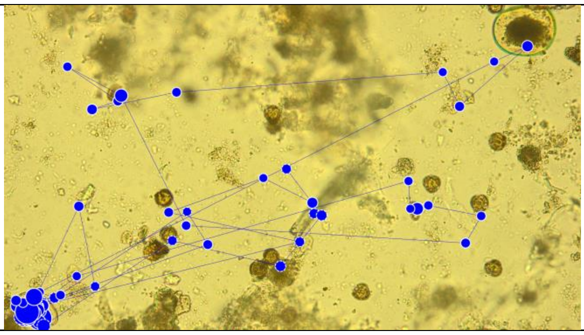

P33

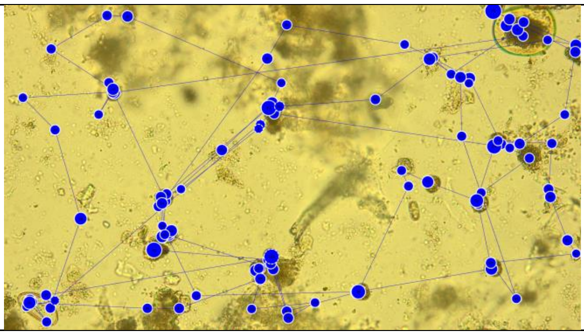

P34

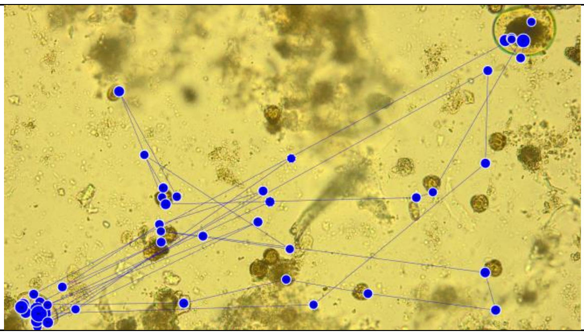

P35

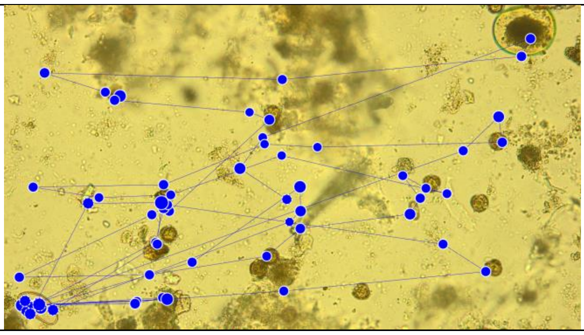

P36

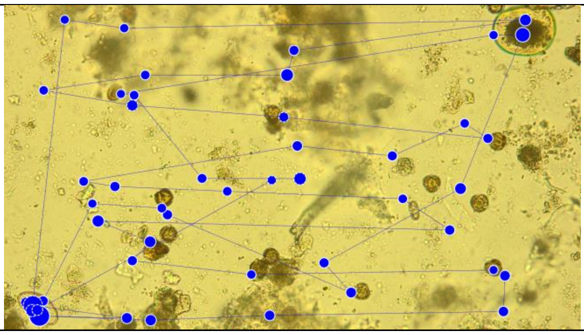

P38

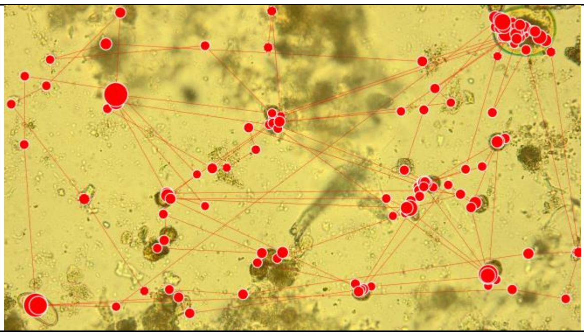

P39

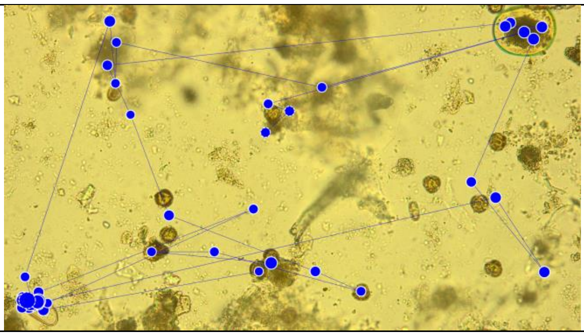

P44

Journal of
Clinical Medicine

MDPI

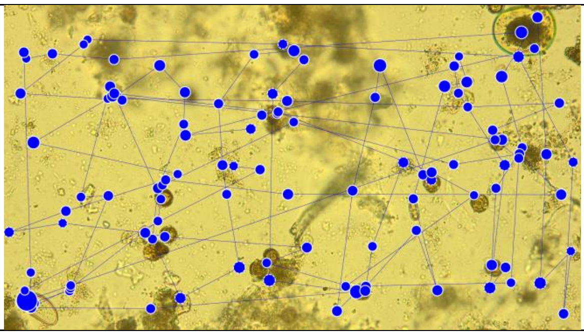

P45

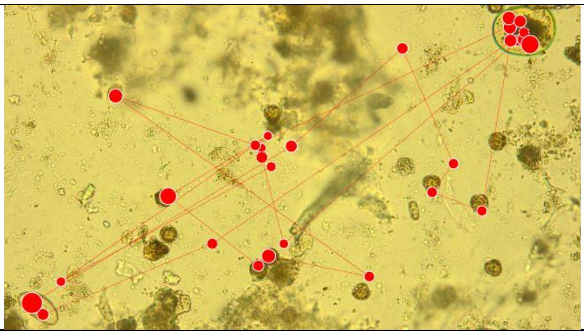

P47

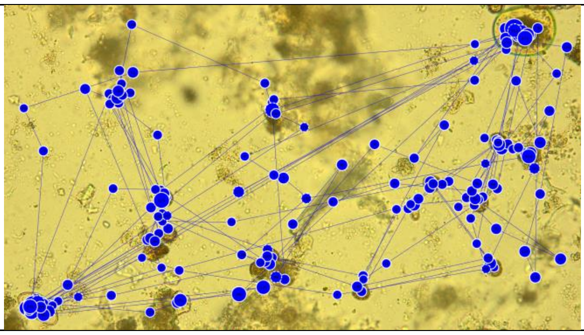

P48

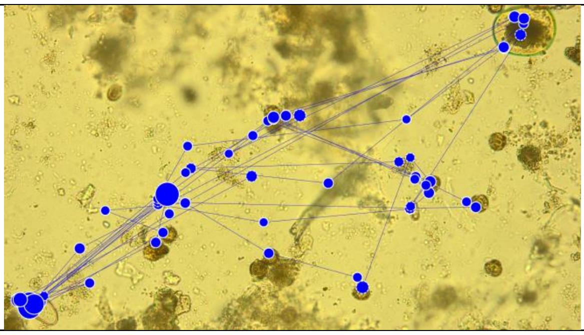

P49

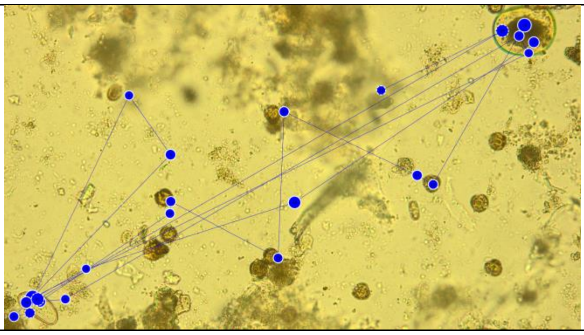

P53

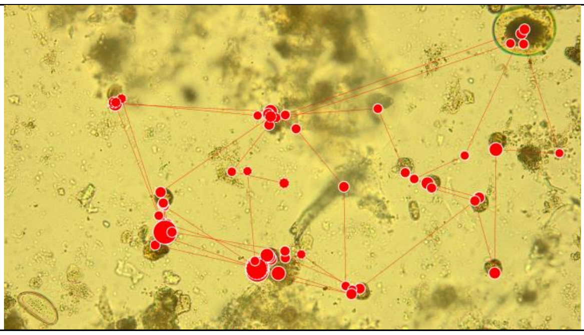

P54

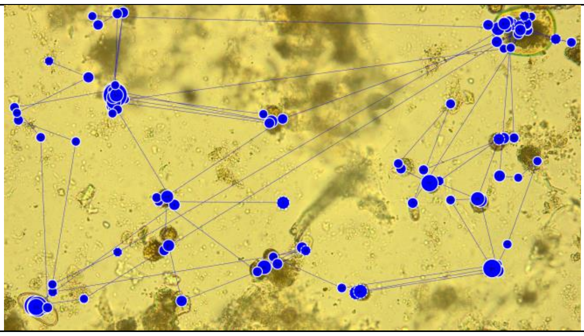

P57

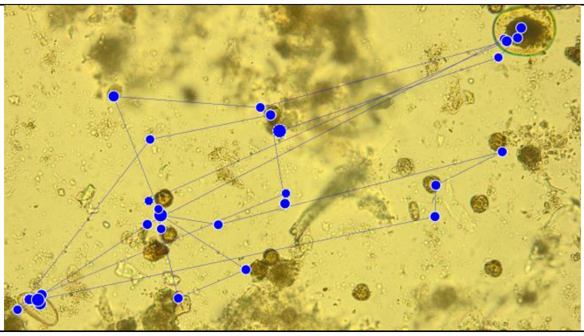

P58

Journal of
Clinical Medicine

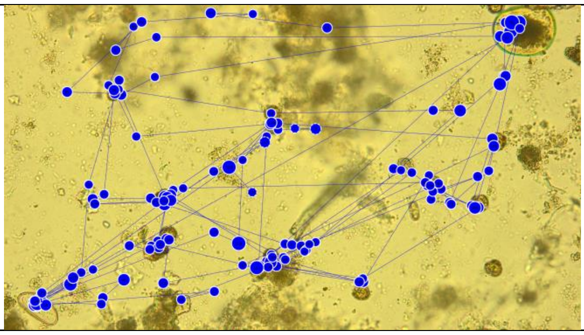	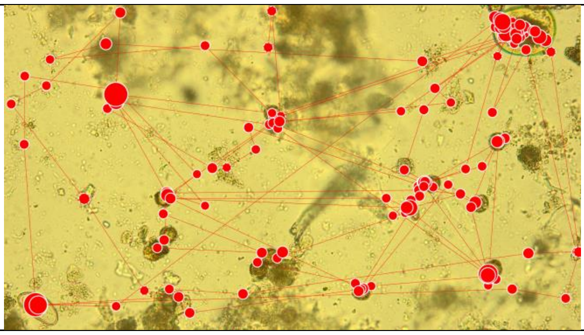
P64	P66
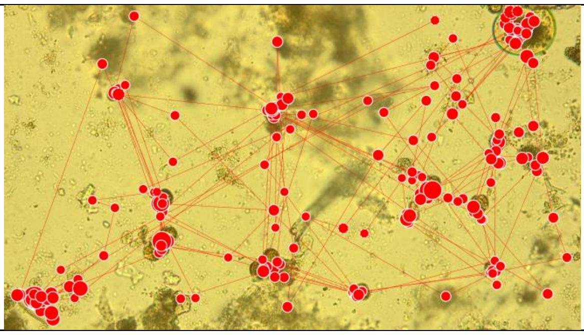	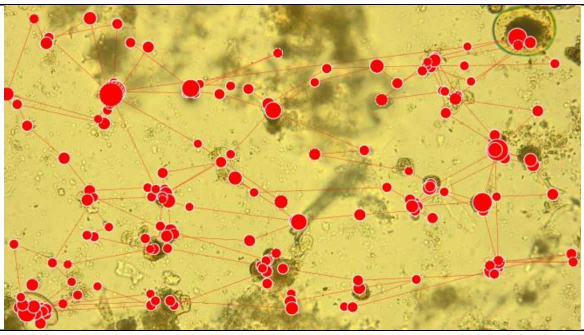
P73	P74
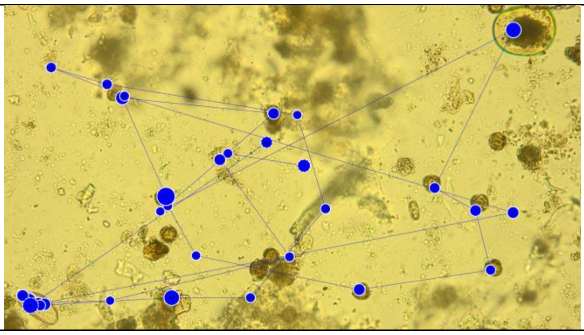	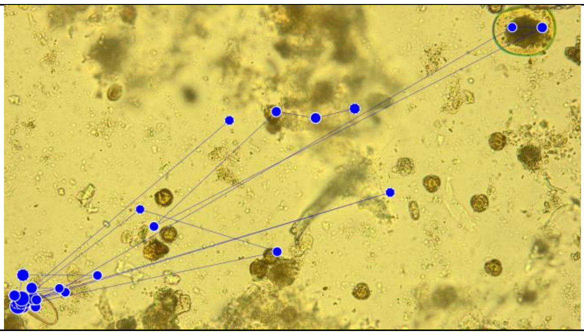
P75	P76
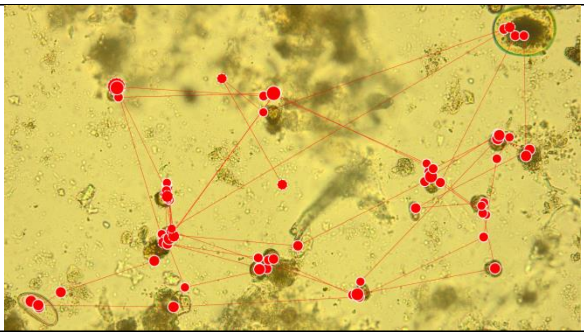	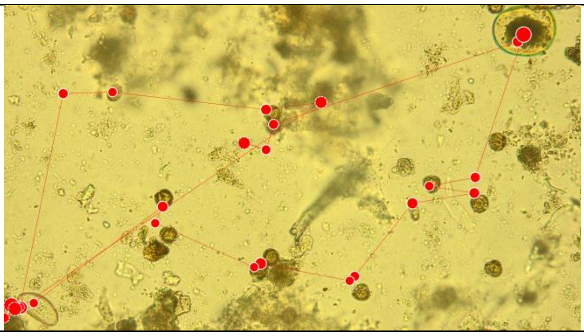
P77	P85

Journal of
Clinical Medicine

MDPI

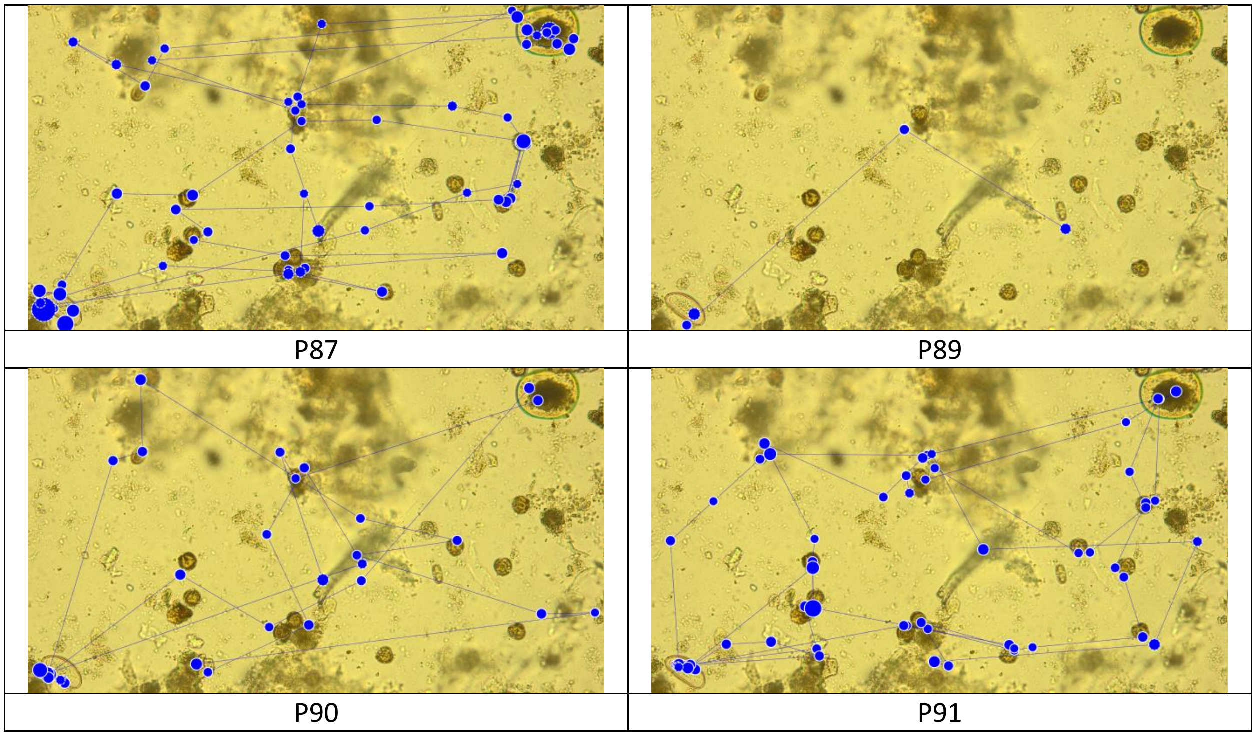

Figure S17. Scanning paths based on the analysis of preparation D - *Enterobius vermicularis* for participants who obtained the average scores. Blue scanning paths - correct diagnosis, red scanning paths – incorrect diagnosis; dot - fixation (eye hold); dot diameter is directly proportional to the time the eye is held; line - saccade (quick shift of eyesight from one point to another).

In the included in the supplement - the collection of scan paths, for individual participants who took part in the research, there is no path for participant P1. Despite the fact that all the eye tracking data and the participant's responses were correctly registered, the software did not generate the scan paths due to an unidentified system error.

Scanning paths based on the analysis of preparation D - *Enterobius vermicularis* for participants who obtained the lowest scores.

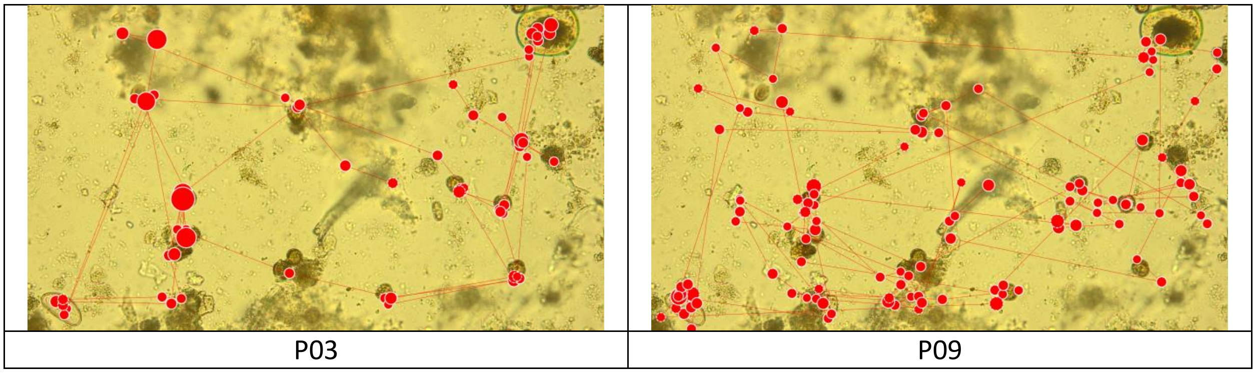

Journal of
Clinical Medicine

MDPI

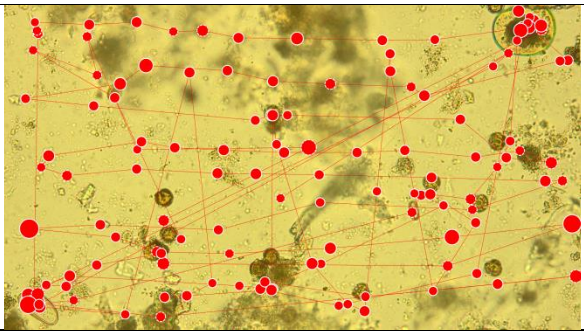

P30

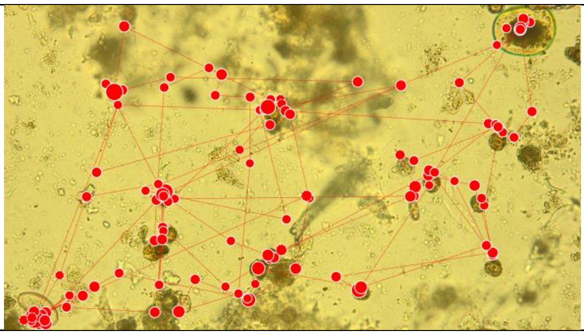

P37

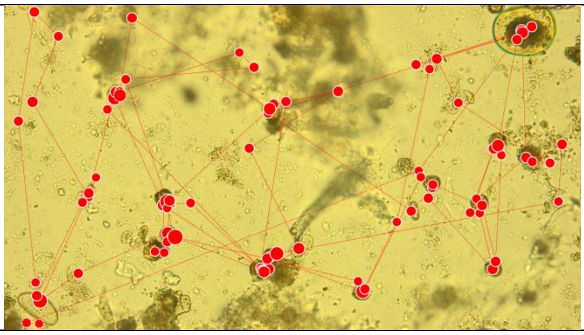

P43

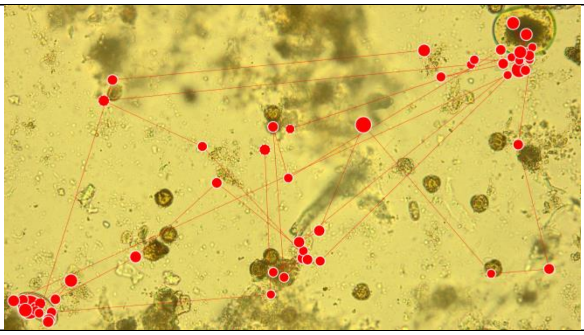

P46

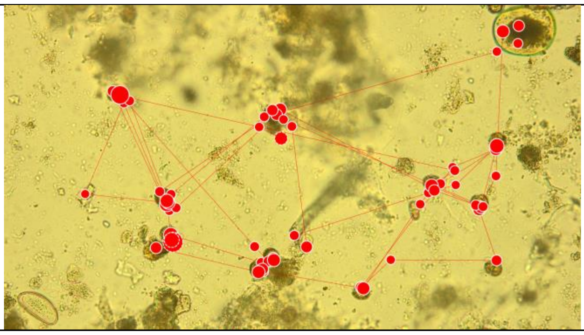

P52

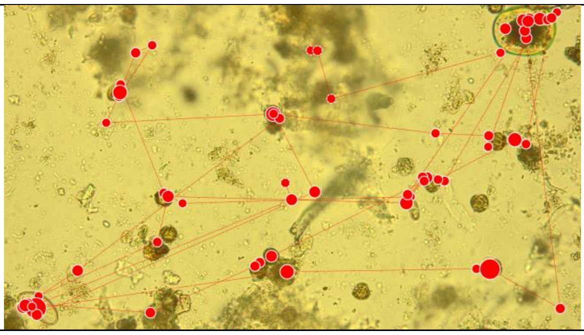

P55

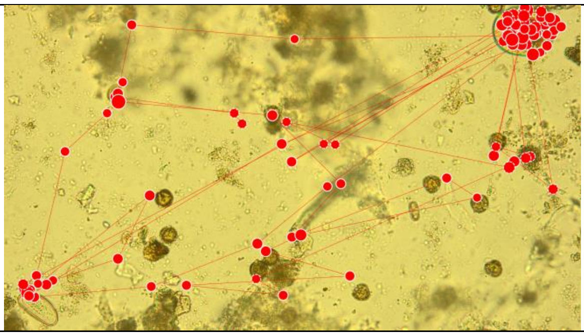

P59

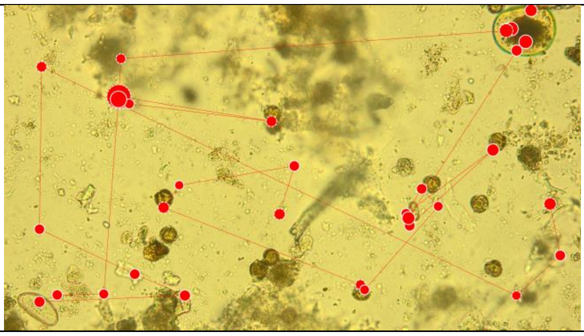

P60

Journal of
Clinical Medicine

MDPI

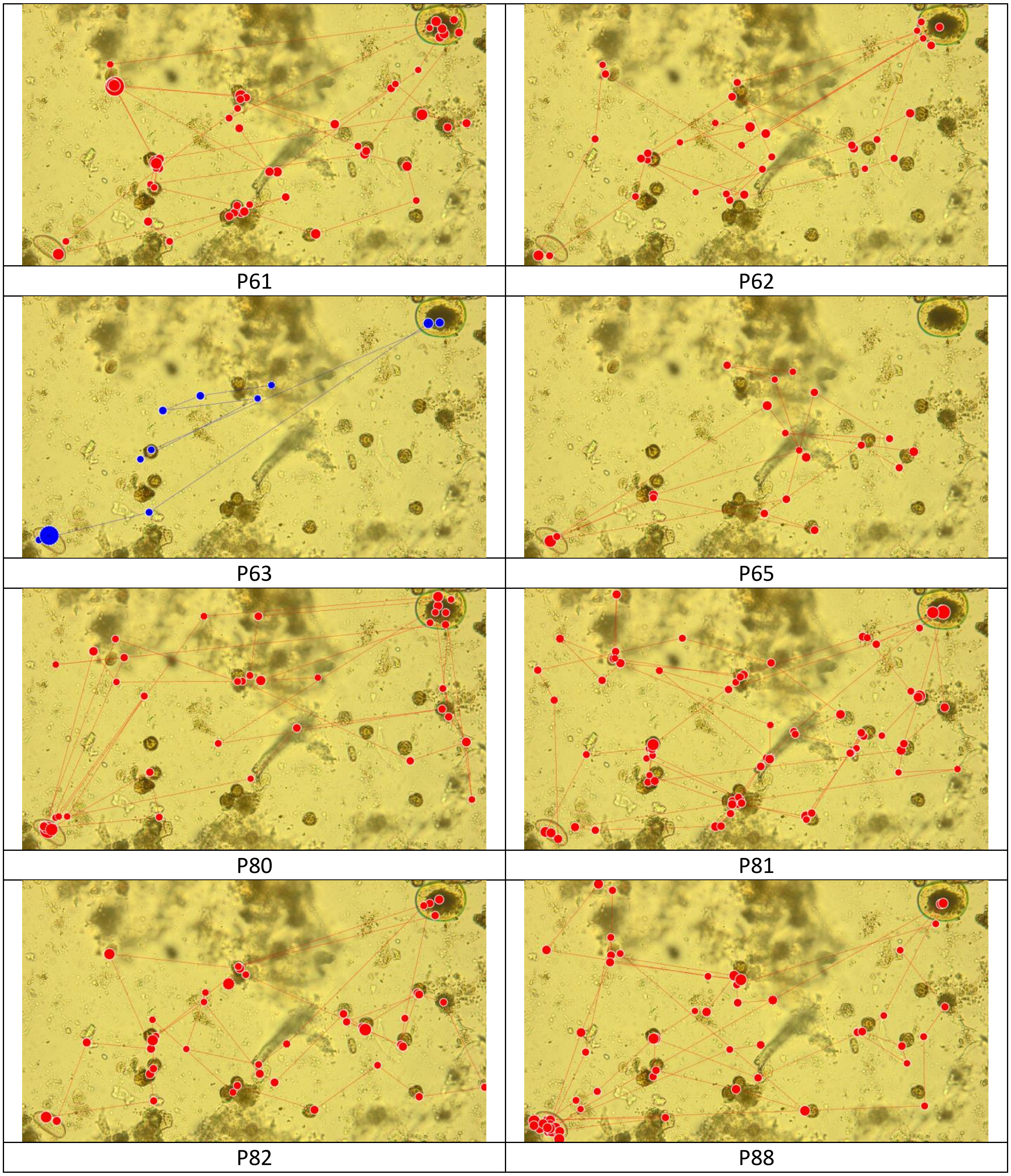

Figure S18. Scanning paths based on the analysis of preparation D - *Enterobius vermicularis* for participants who obtained the lowest scores. Blue scanning paths - correct diagnosis, red scanning paths – incorrect diagnosis; dot - fixation (eye hold); dot diameter is directly proportional to the time the eye is held; line - saccade (quick shift of eyesight from one point to another).
