# Supplementary material for: Eye Tracking—An Innovative Tool in Medical Parasitology
Source: J Clin Med. 2021 Jul 4;10(13):2989. doi: 10.3390/jcm10132989 (PMC8268455; doi:10.3390/jcm10132989)
Supplement: Supplementary file 1 [file jcm-10-02989-s001.zip › Supplementary materials - Qualitative analysis_heatmap.pdf]

Journal of
Clinical Medicine

MDPI

Supplementary Materials: Eye tracking – an innovative tool in medical parasitology

Przemysław Kołodziej [1,*], Wioletta Tuszyńska-Bogucka [2], Mariusz Dzieńkowski [3], Jacek Bogucki [4], Janusz Kocki [5], Marek Milosz [3], Marcin Kocki [6], Patrycja Reszka [6], Wojciech Kocki [7] and Anna Bogucka-Kocka [1]

Qualitative analysis- heat maps

Journal of Clinical Medicine

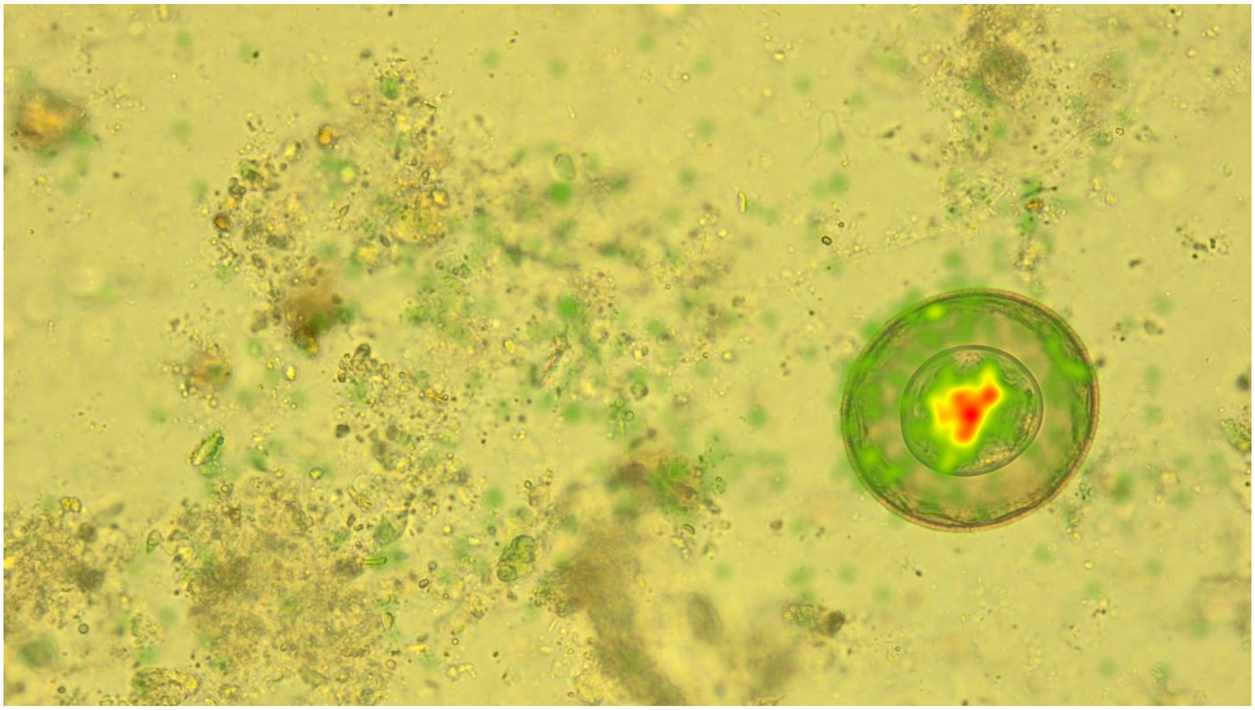

(Figure 19S, A)

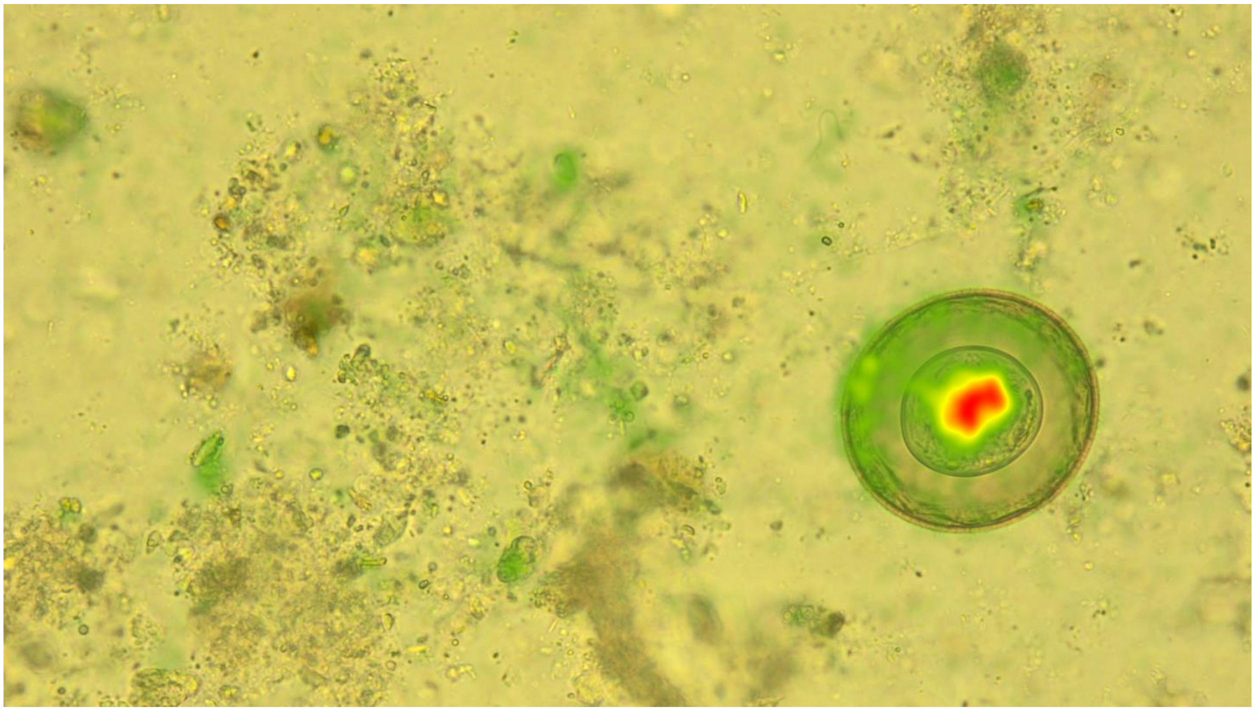

(Figure 19S, B)

Figure S19. Heat map based on the analysis of preparation A - *Hymenolepis diminuta* (A - correct diagnosis of all participants, B - incorrect diagnosis of all participants. A heat map is a type of information presented, showing the number of gazes (fixations), i.e. how many times the respondents looked at a given area.

Journal of
Clinical Medicine

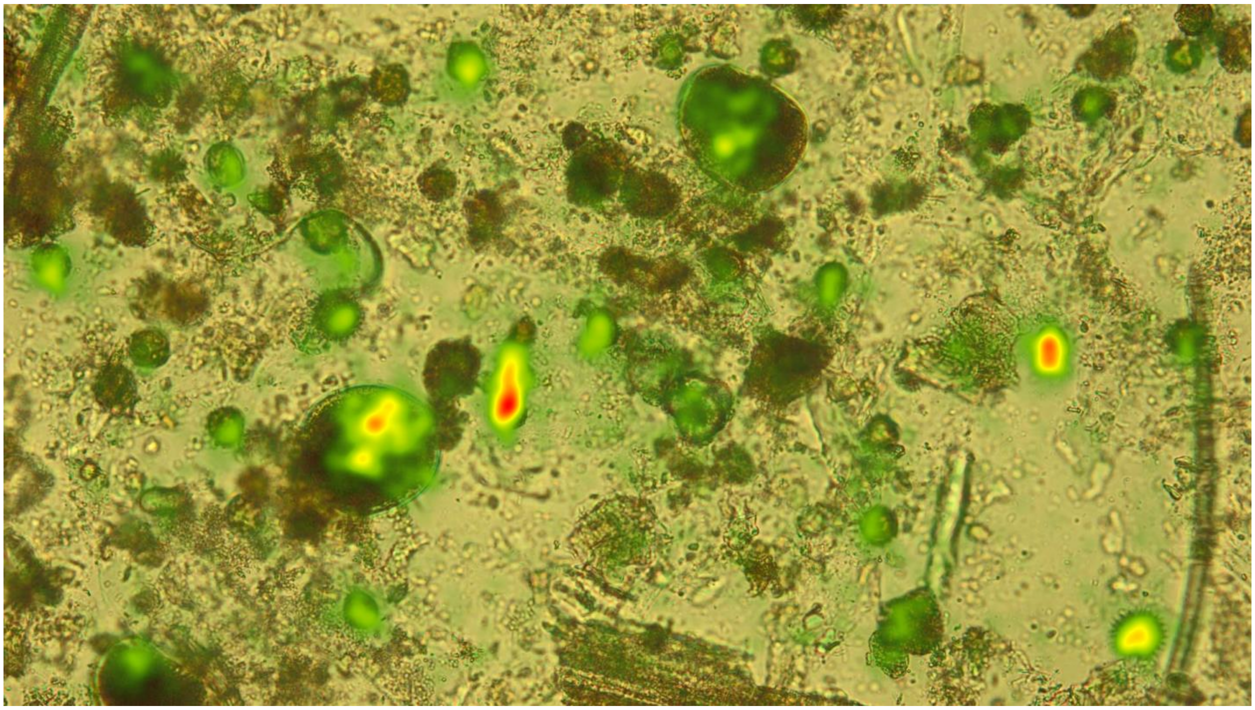

(Figure 20S, A)

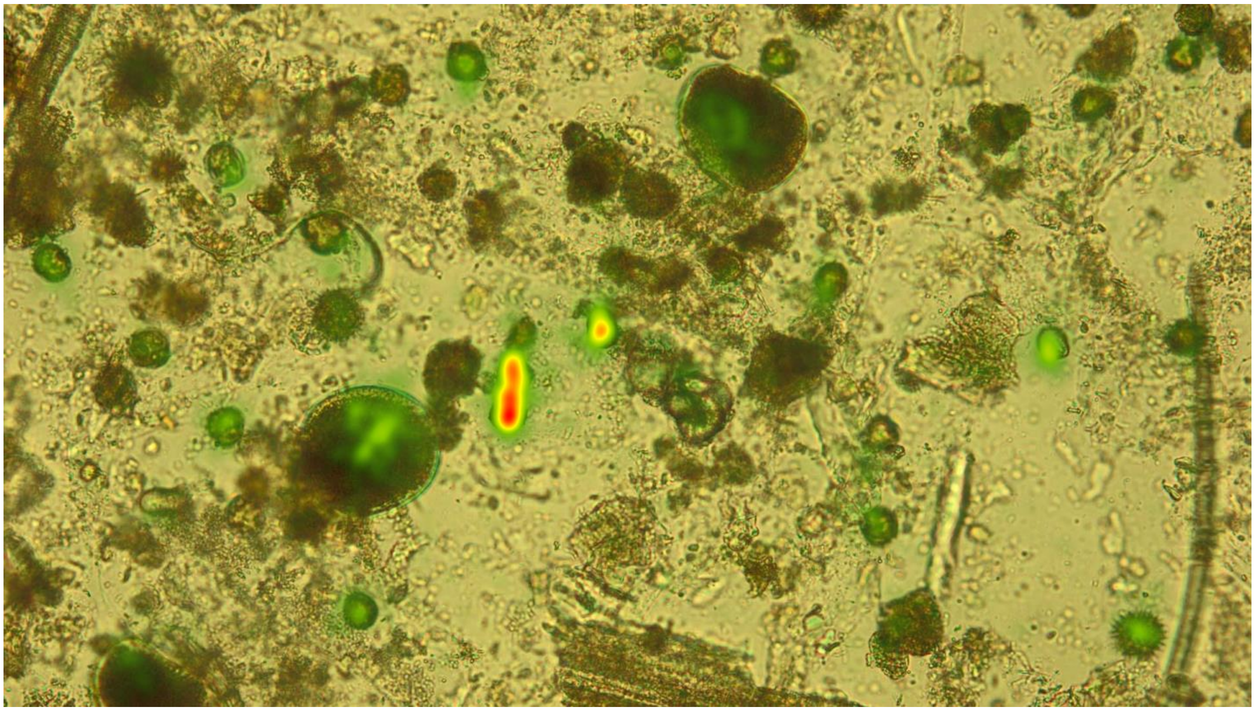

(Figure 20S, B)

Figure S20. Heat map based on the analysis of preparation B - Artefacts (A - correct diagnosis of all participants, B - incorrect diagnosis of all participants. A heat map is a type of information presented, showing the number of gazes (fixations), i.e. how many times the respondents looked at a given area.

Journal of
Clinical Medicine

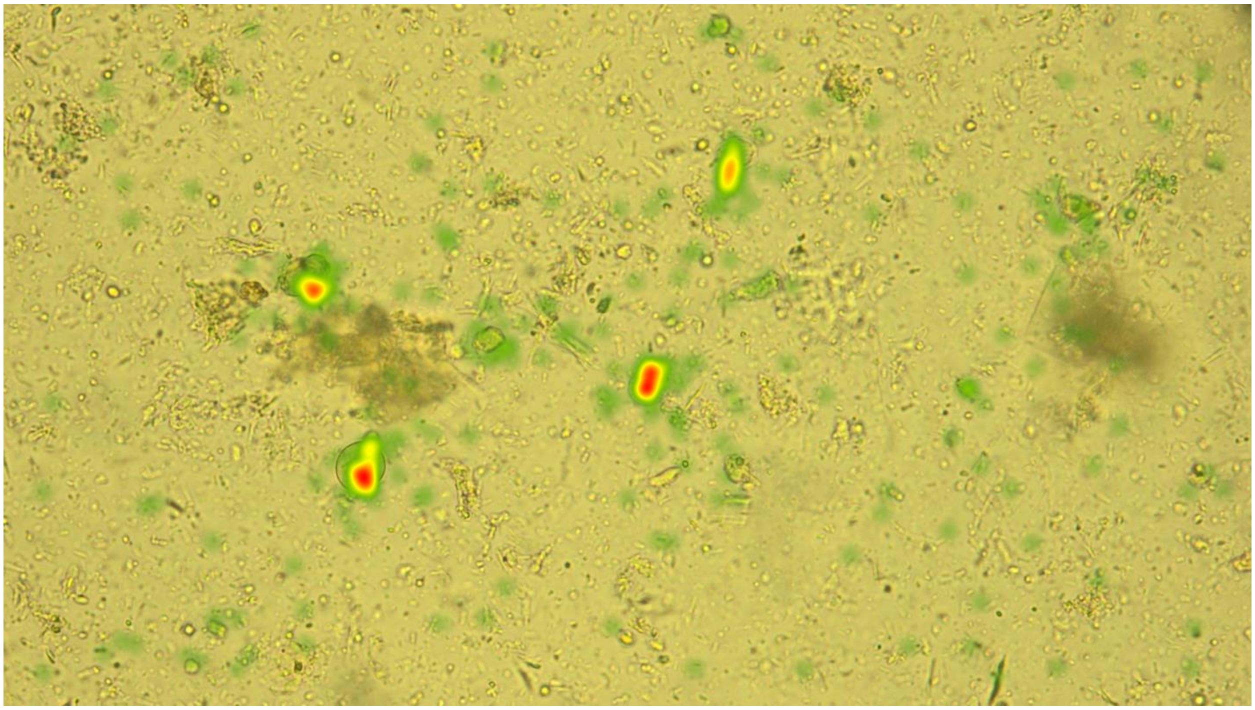

(Figure 21S, A)

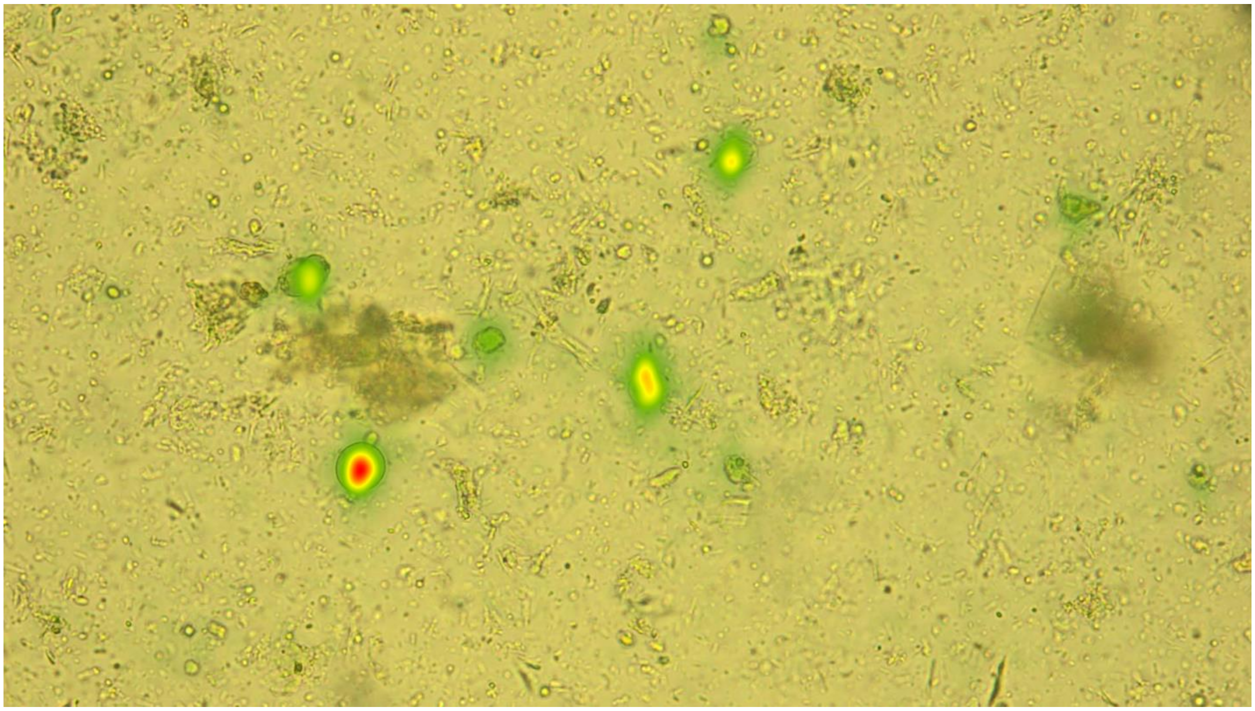

(Figure 21S, B)

Figure S21. Heat map based on the analysis of preparation E - *Entamoeba sp*. and *Giardia intestinalis* (A - correct diagnosis of all participants, B - incorrect diagnosis of all participants. A heat map is a type of information presented, showing the number of gazes (fixations), i.e. how many times the respondents looked at a given area.

Journal of
Clinical Medicine

MDPI

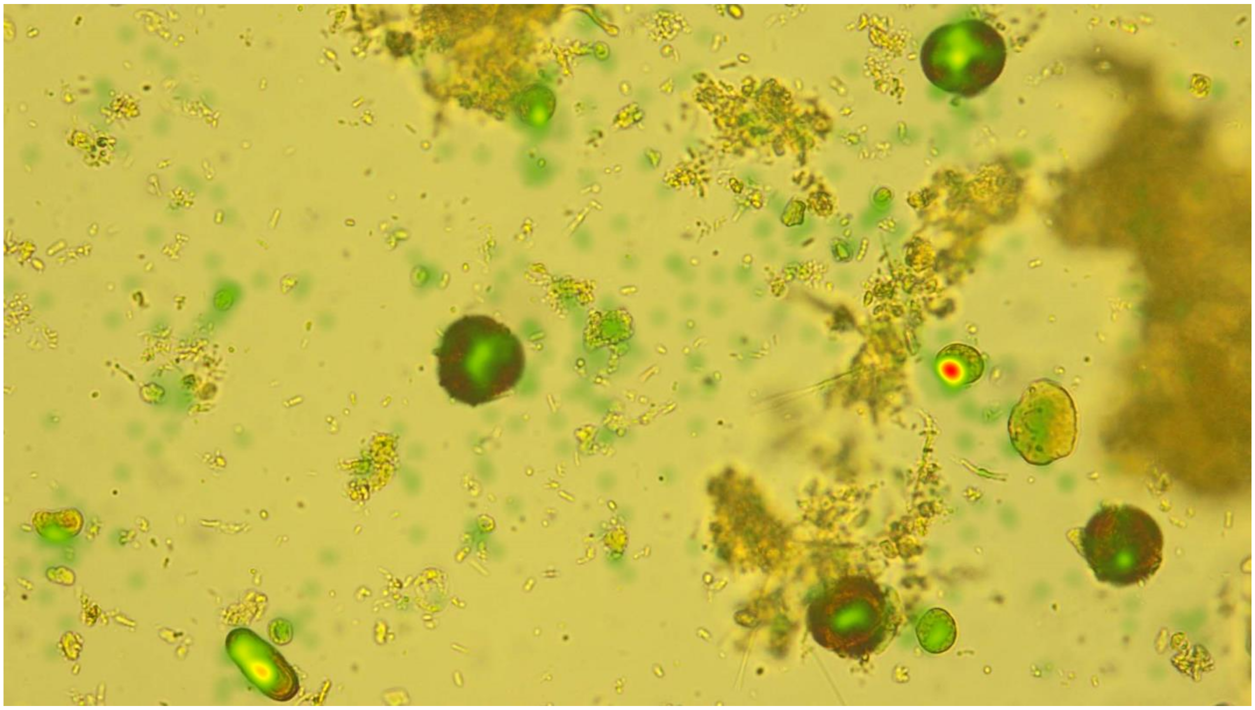

(Figure 22S, A)

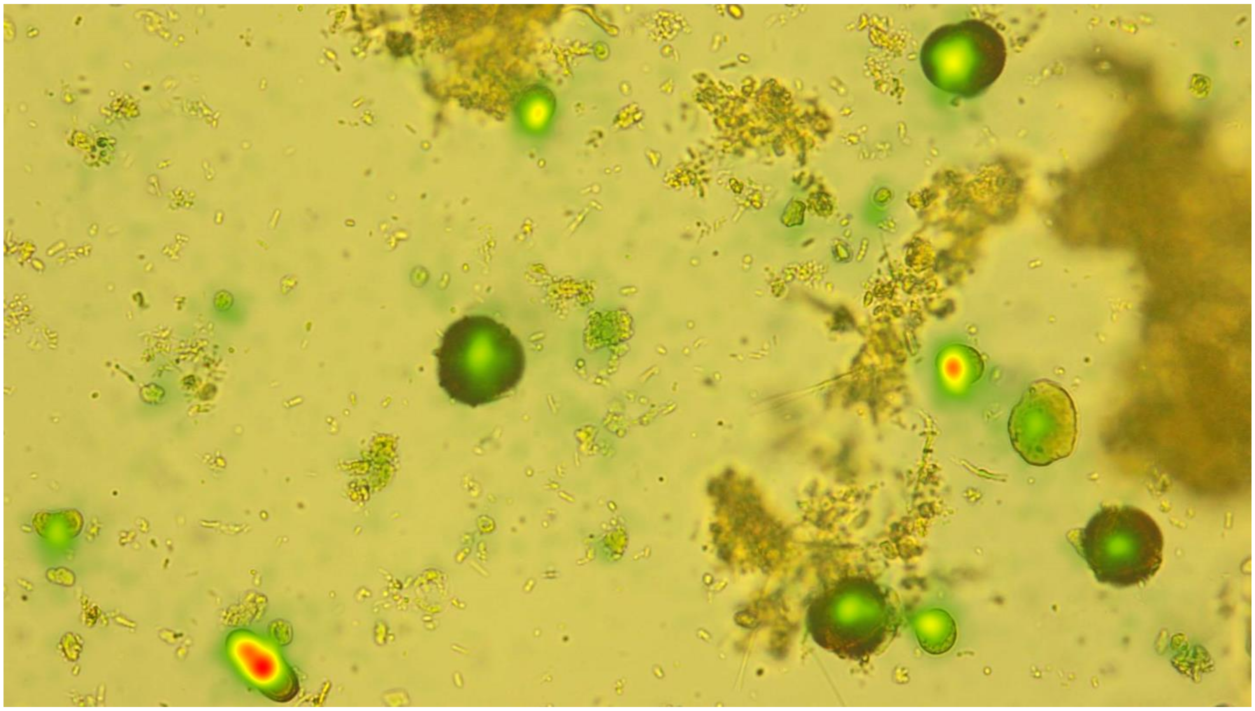

(Figure 22S, B)

Figure S22. Heat map based on the analysis of preparation F - *Iodamoeba bütschlii* and artefacts. (A - correct diagnosis of all participants, B - incorrect diagnosis of all participants. A heat map is a type of information presented, showing the number of gazes (fixations), i.e. how many times the respondents looked at a given area.
