# Supplementary material for: Eye Tracking—An Innovative Tool in Medical Parasitology
Source: J Clin Med. 2021 Jul 4;10(13):2989. doi: 10.3390/jcm10132989 (PMC8268455; doi:10.3390/jcm10132989)
Supplement: Supplementary file 1 [file jcm-10-02989-s001.zip › Supplementary materials - Quantitative analysis.pdf]

Journal of
Clinical Medicine

MDPI

Supplementary Materials: Eye tracking – an innovative tool in medical parasitology

Przemysław Kołodziej [1,*], Wioletta Tuszyńska-Bogucka [2], Mariusz Dzieńkowski [3], Jacek Bogucki [4], Janusz Kocki [5], Marek Milosz [3], Marcin Kocki [6], Patrycja Reszka [6], Wojciech Kocki [7] and Anna Bogucka-Kocka [1]

Quantitative analysis

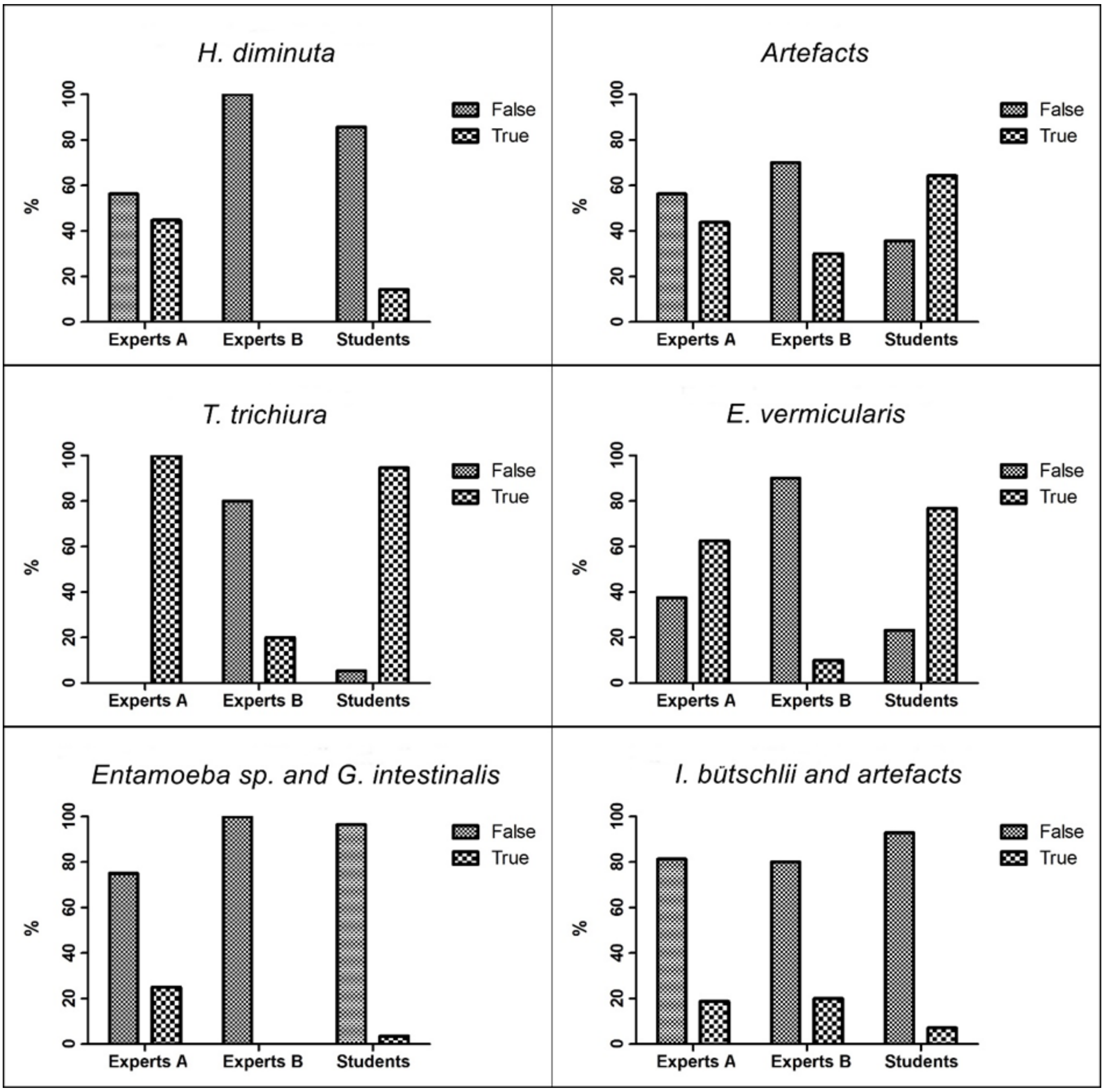

Figure S1. Distribution of correct and incorrect diagnoses in groups.

Journal of
Clinical Medicine

Table S1. The results of the analysis of the significance of differences in the values of the analyzed parameters in the study groups with extreme survey results (U Mann-Whitney test).

Variables	Sum.of rang Group l Lowest scores	Sum.of rang Group II Highest scores	U	Z	p
x Ratio	256,0000	179,0000	85,00000	-0,62925	0,529
Time of display	154,0000	171,0000	49,00000	-1,53286	0,125
Time of questionnaire display	193,0000	132,0000	66,00000	0,60219	0,547
Number of fixations	158,5000	166,5000	53,50000	-1,28650	0,198
Mean time of fixation	182,0000	143,0000	77,00000	0,00000	---
Number of sacades	158,5000	166,5000	53,50000	-1,28650	0,198
Mean time of sacedes	195,0000	130,0000	64,00000	0,71168	0,476
Amplitude of sacades	196,0000	129,0000	63,00000	0,76643	0,443
Horizontal sacades	145,5000	179,5000	40,50000	-1,99819	0,045
Vertical sacades	170,5000	154,5000	65,50000	-0,62957	0,528
Diagonal sacades	151,0000	149,0000	46,00000	-1,40530	0,159

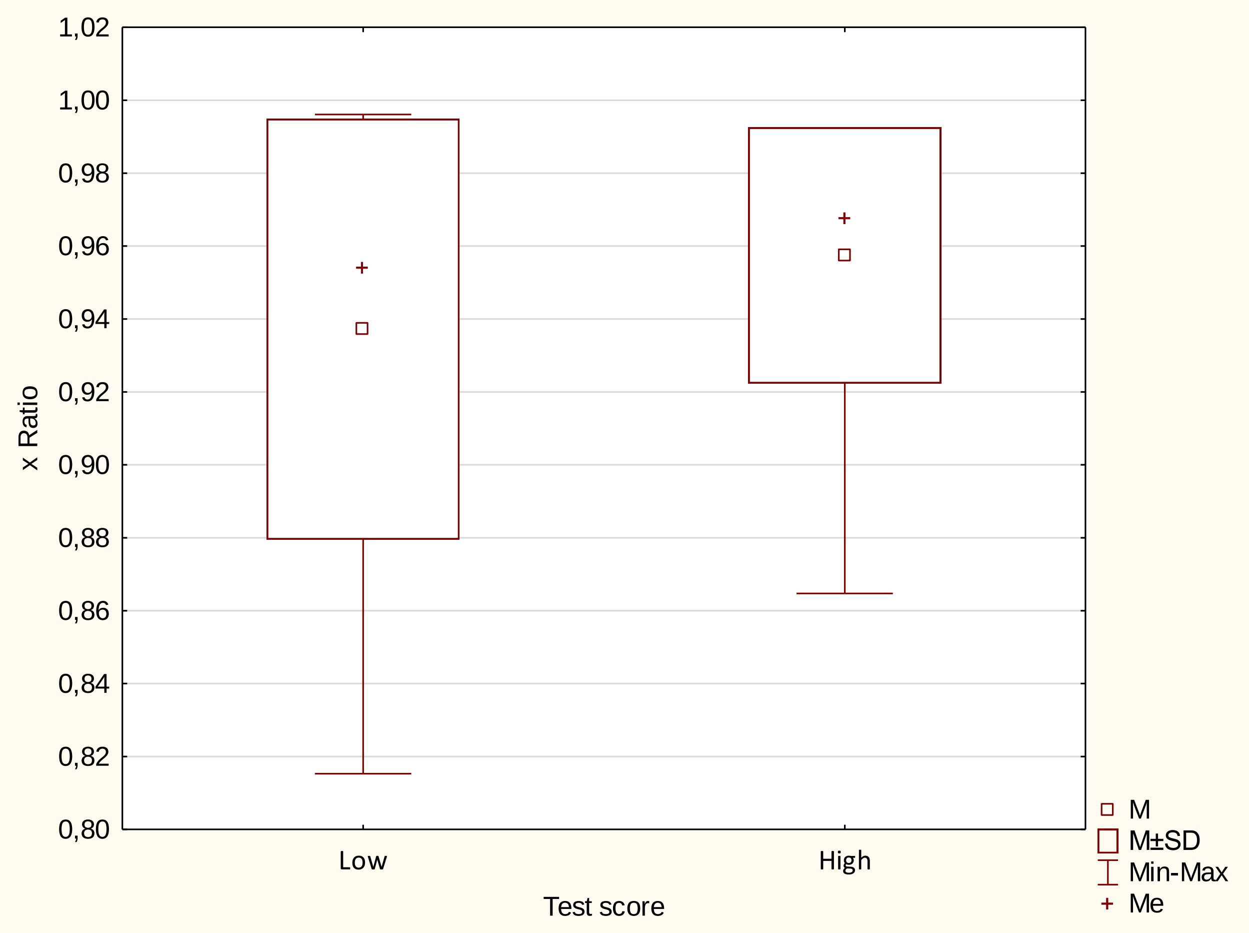

Figure S2. Descriptive statistics of the “x Ratio” variable in groups with extreme test results (Low - lowest scores, High - highest scores).

Journal of
Clinical Medicine

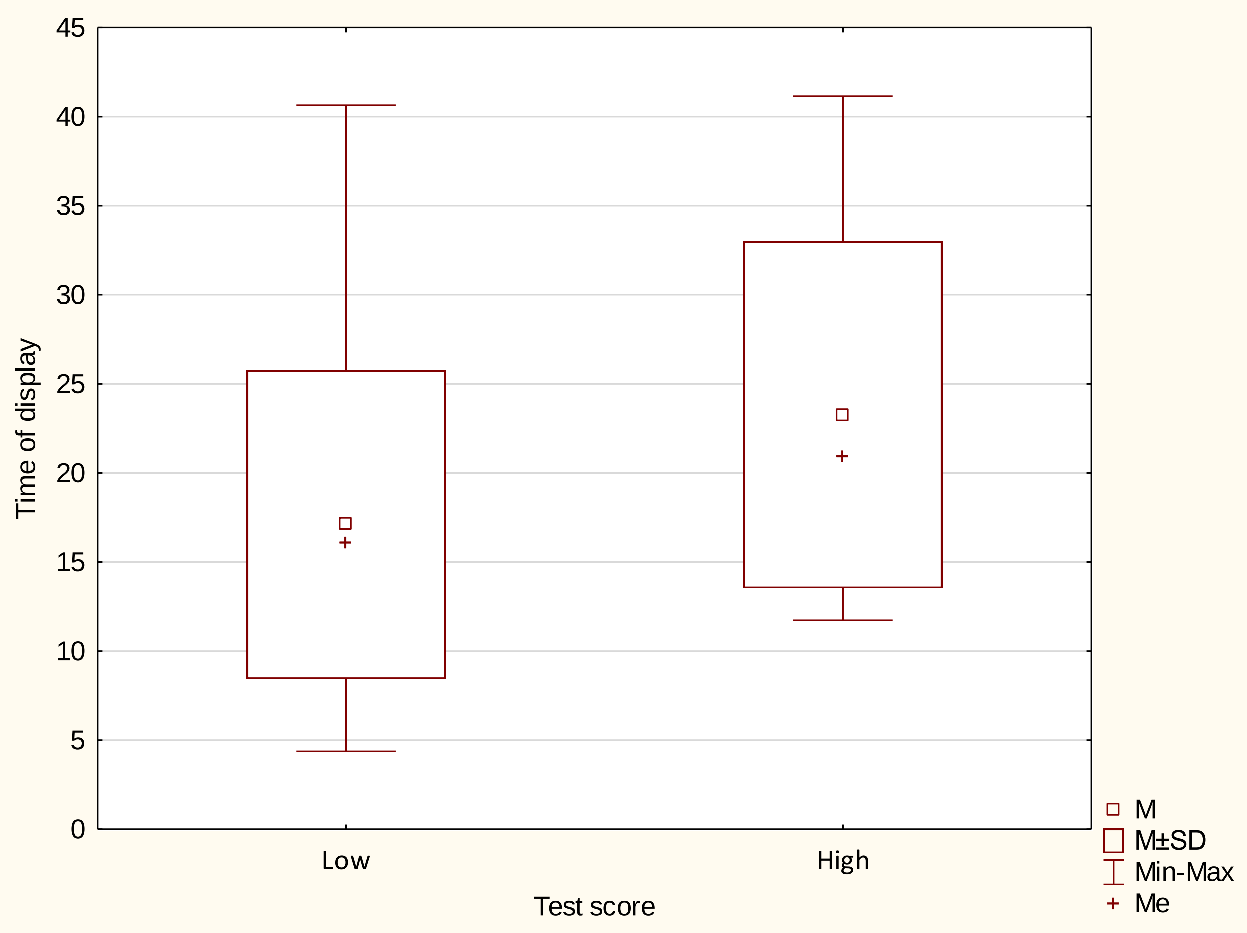

Figure S3. Descriptive statistics of the “Time of display” variable in groups with extreme test results (Low - lowest scores, High - highest scores).

Journal of
Clinical Medicine

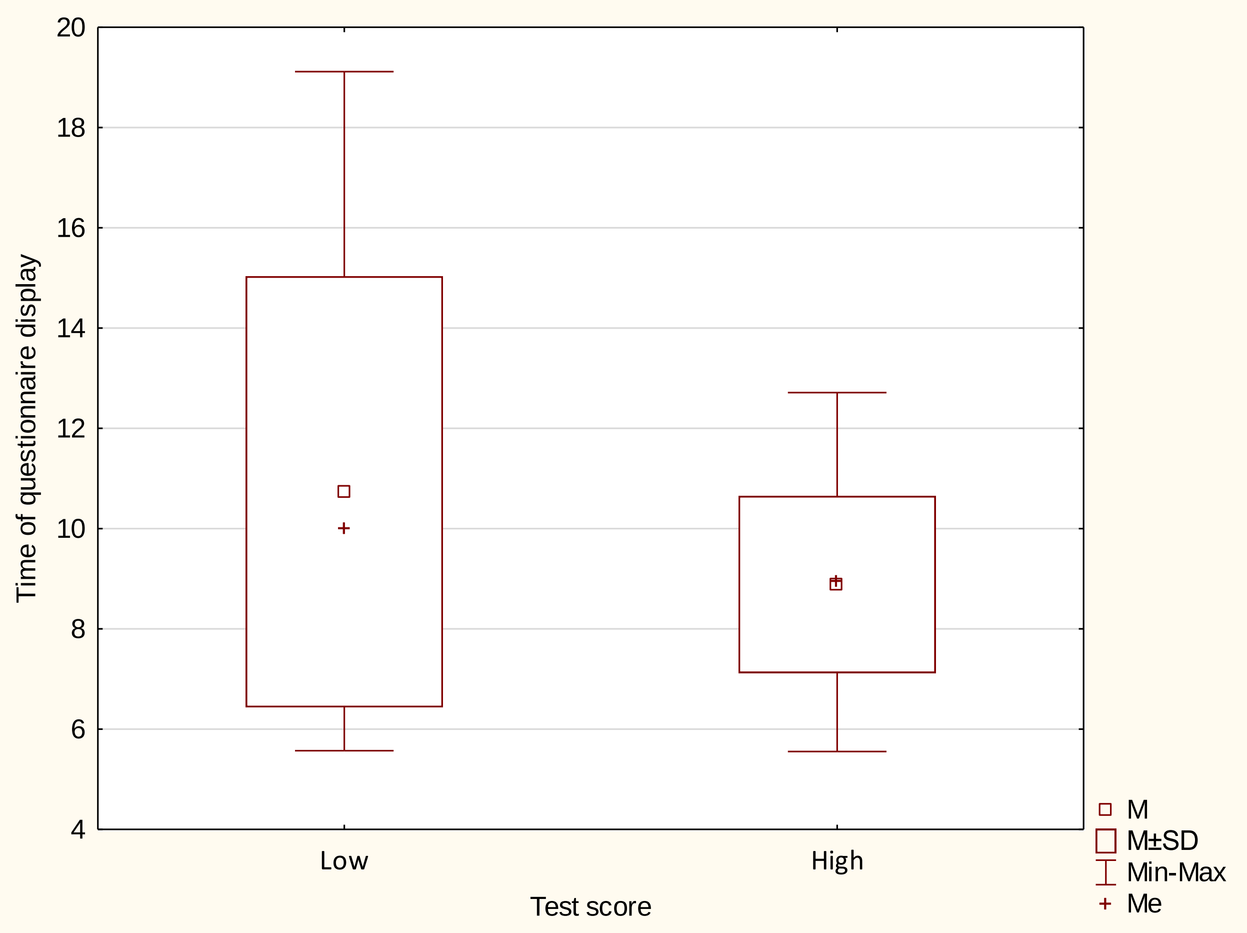

Figure S4. Descriptive statistics of the "Time of questionnaire display" variable in groups with extreme test results (Low - lowest scores, High - highest scores).

Journal of
Clinical Medicine

MDPI

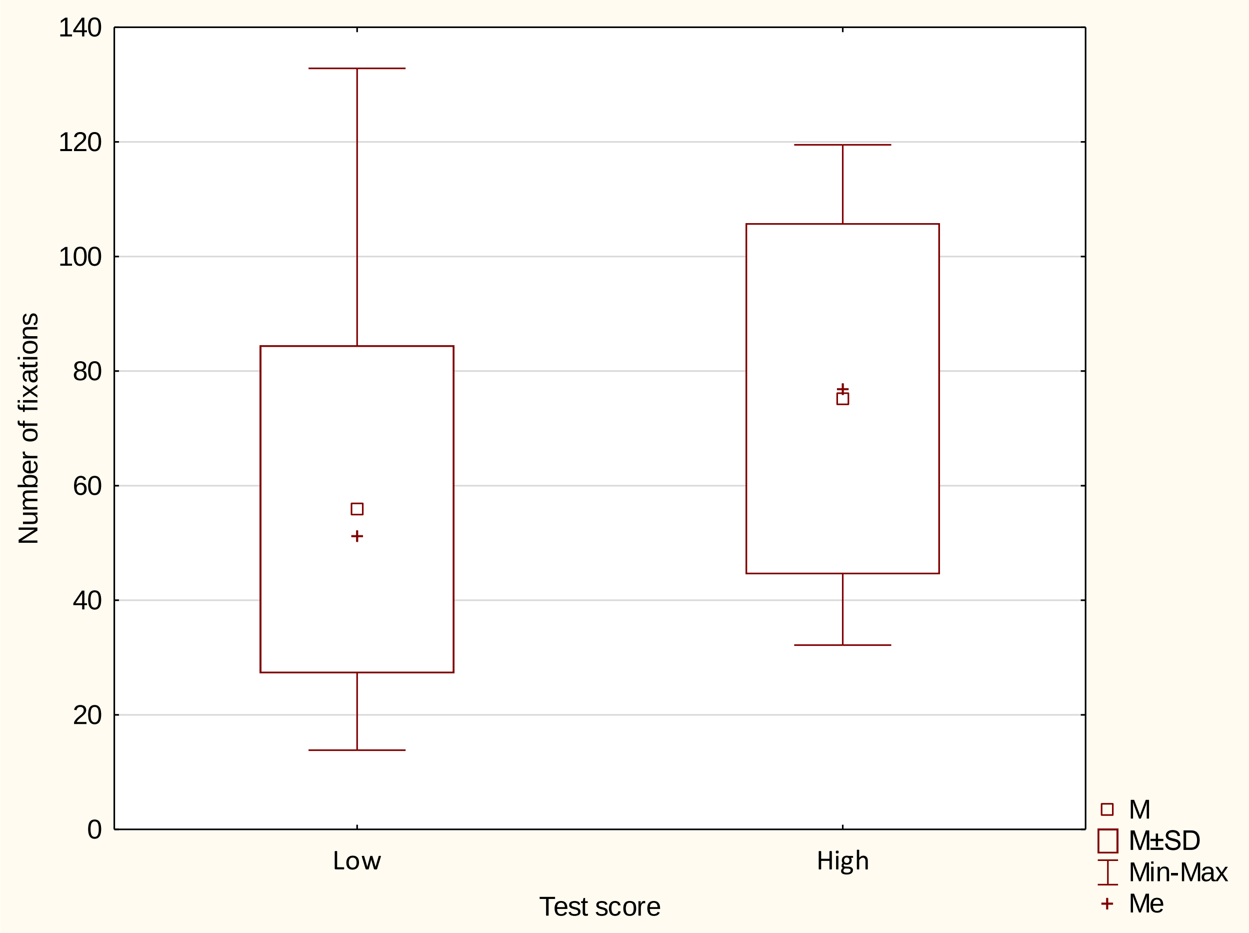

Figure S5. Descriptive statistics of the "Number of fixation" variable in groups with extreme test results (Low - lowest scores, High - highest scores).

Journal of
Clinical Medicine

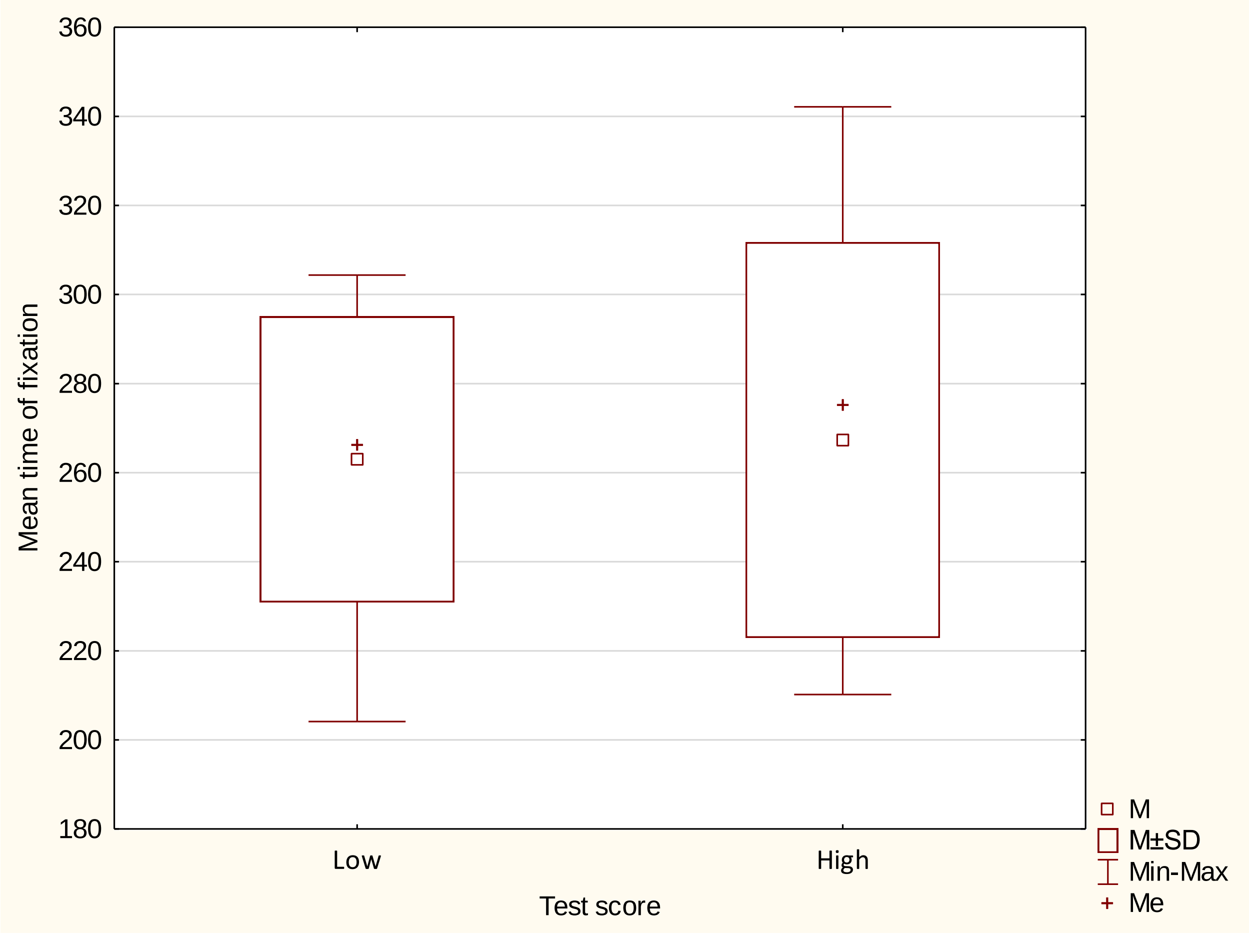

Figure S6. Descriptive statistics of the "Mean time of fixation" variable in groups with extreme test results (Low - lowest scores, High - highest scores).

Journal of
Clinical Medicine

MDPI

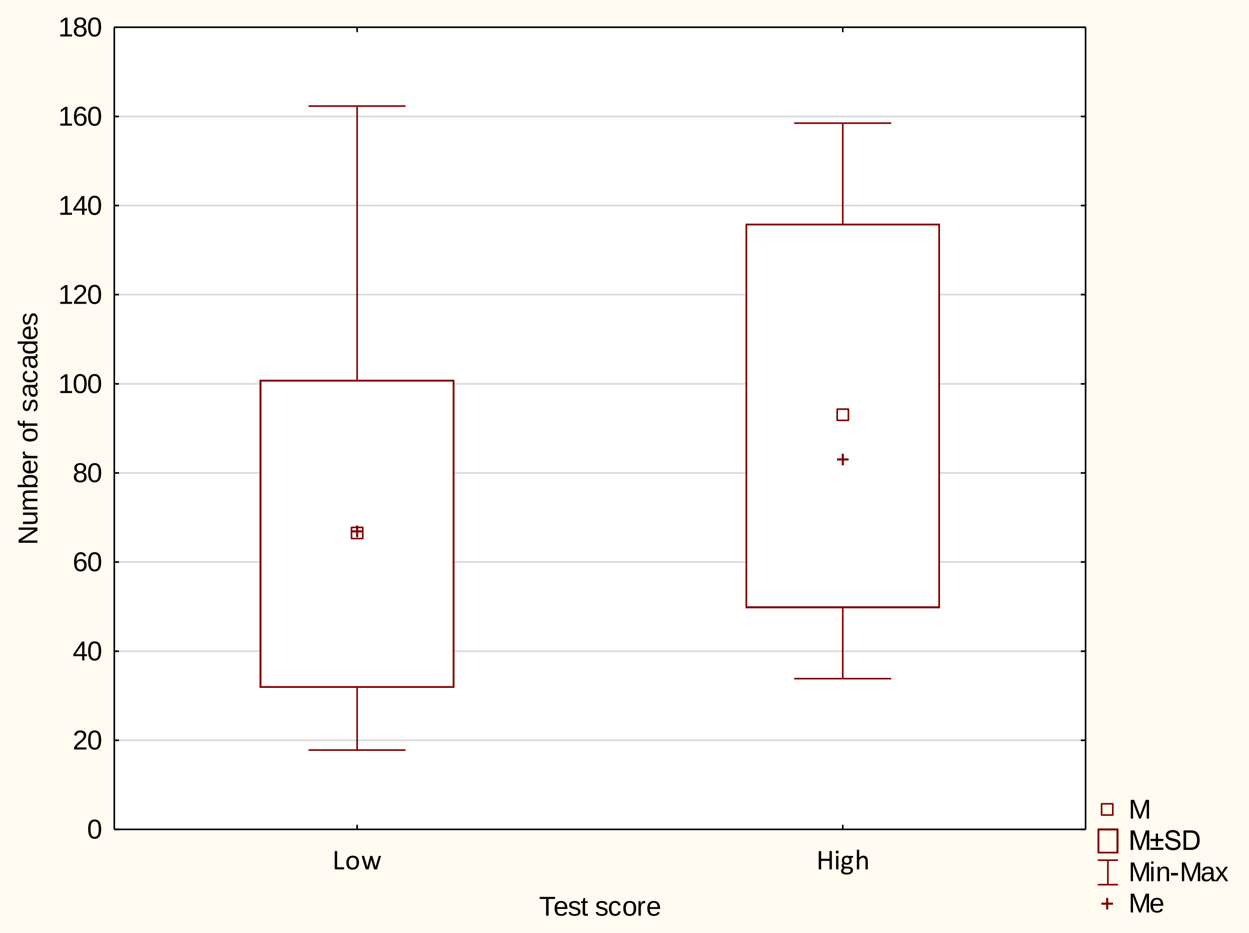

Figure S7. Descriptive statistics of the "Number of sacades" variable in groups with extreme test results (Low - lowest scores, High - highest scores).

Journal of
Clinical Medicine

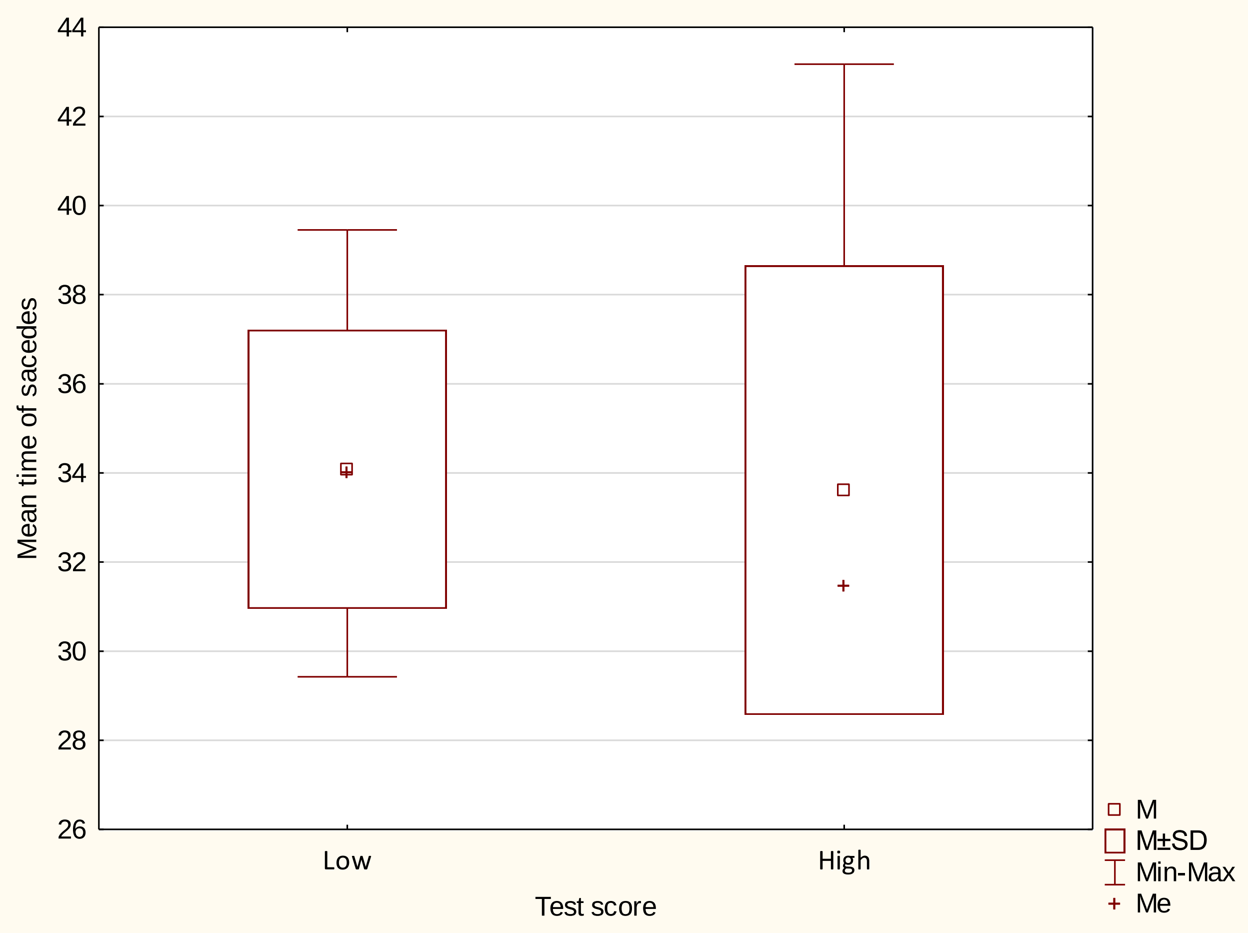

Figure S8. Descriptive statistics of the "Mean time of sacades" variable in groups with extreme test results (Low - lowest scores, High - highest scores).

Journal of
Clinical Medicine

MDPI

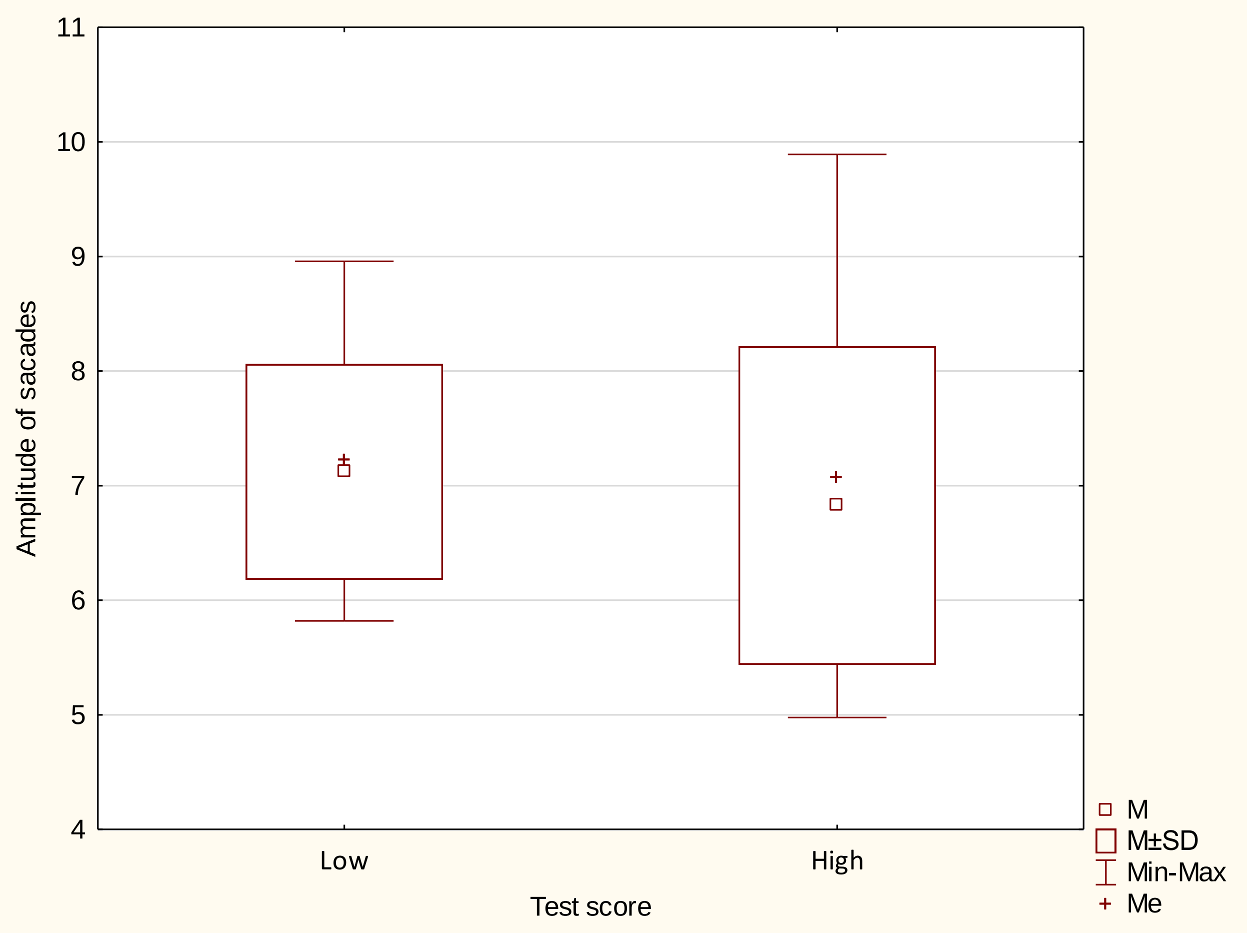

Figure S9. Descriptive statistics of the "Amplitude of sacades" variable in groups with extreme test results (Low - lowest scores, High - highest scores).

Journal of
Clinical Medicine

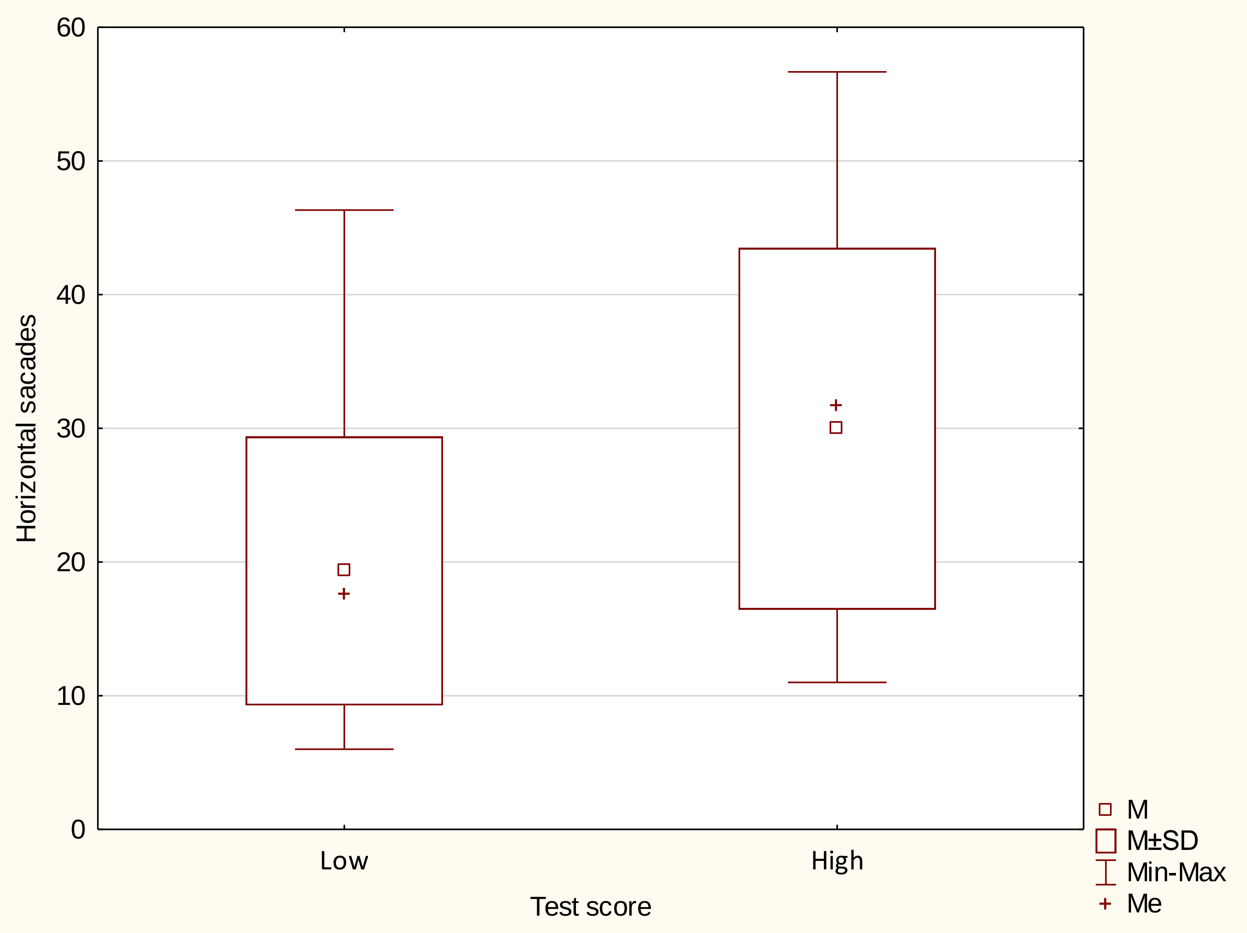

Figure S10. Descriptive statistics of the "Horizontal sacades" variable in groups with extreme test results (Low - lowest scores, High - highest scores).

Journal of
Clinical Medicine
MDPI

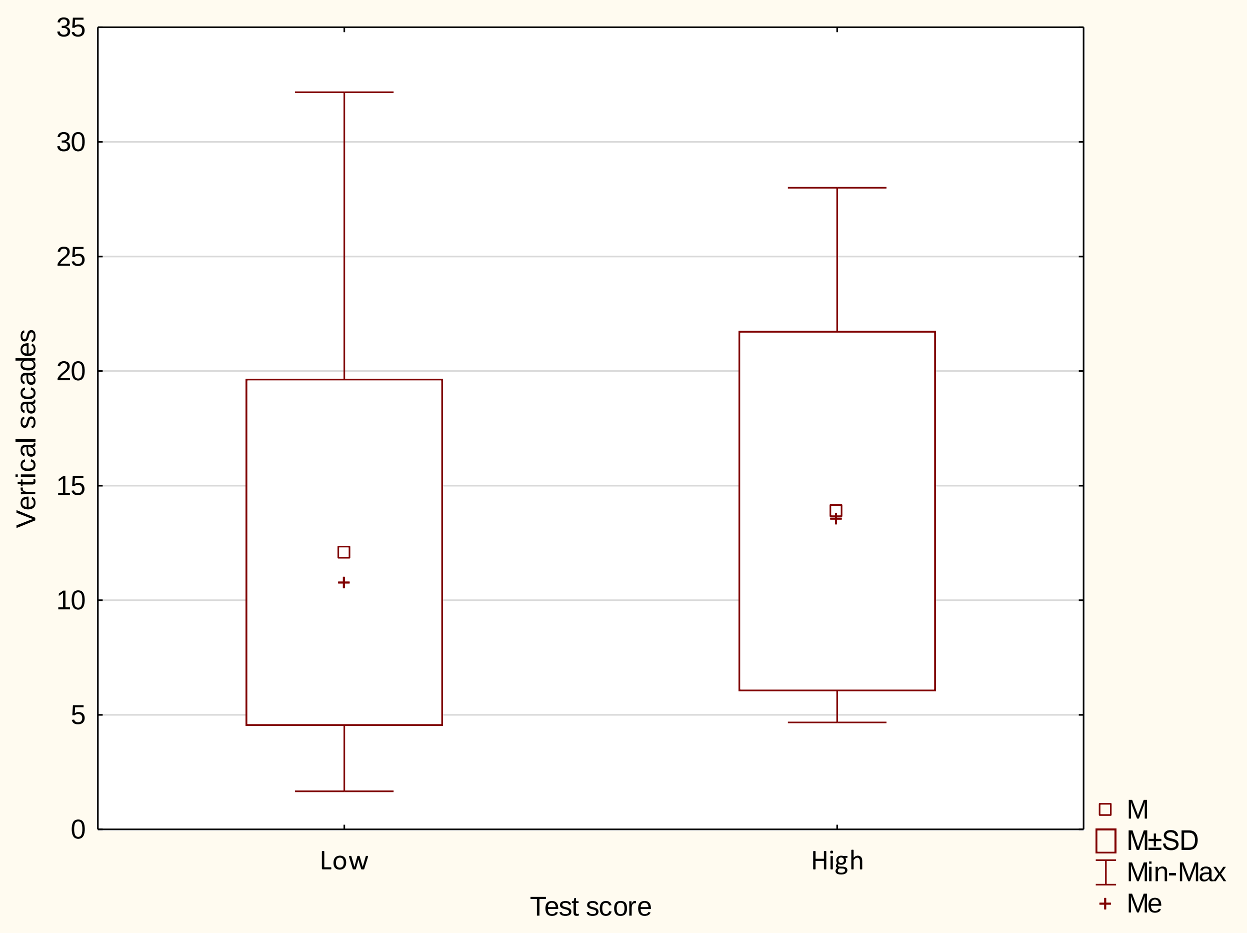

Figure S11. Descriptive statistics of the "Vertical sacades" variable in groups with extreme test results (Low - lowest scores, High - highest scores).

Journal of Clinical Medicine

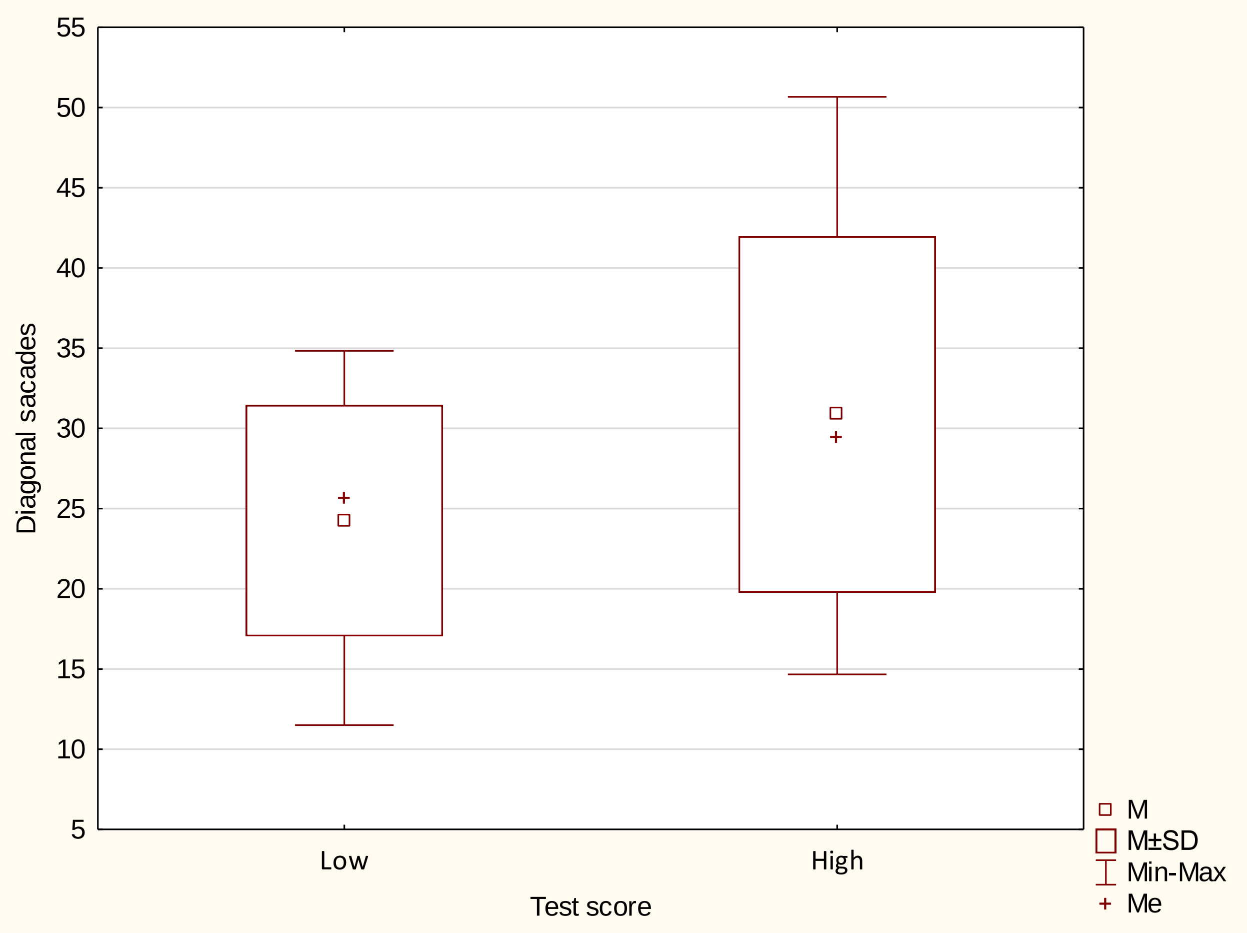

Figure S12. Descriptive statistics of the "Diagonal sacades" variable in groups with extreme test results (Low - lowest scores, High - highest scores).
